# Human-Altered Landscapes and Climate to Predict Human Infectious Disease Hotspots

**DOI:** 10.3390/tropicalmed7070124

**Published:** 2022-07-01

**Authors:** Soushieta Jagadesh, Marine Combe, Rodolphe Elie Gozlan

**Affiliations:** 1 Heath Geography and Policy, ETH Zurich, Sonneggstrasse 33, 8092 Zurich, Switzerland; 2 ISEM, Université de Montpellier, CNRS, IRD, 34090 Montpellier, France; marine.combe@ird.fr (M.C.); rudy.gozlan@ird.fr (R.E.G.)

**Keywords:** emerging infectious diseases, human-altered landscapes, biogeography, topography, climate, land-use

## Abstract

Background: Zoonotic diseases account for more than 70% of emerging infectious diseases (EIDs). Due to their increasing incidence and impact on global health and the economy, the emergence of zoonoses is a major public health challenge. Here, we use a biogeographic approach to predict future hotspots and determine the factors influencing disease emergence. We have focused on the following three viral disease groups of concern: Filoviridae, Coronaviridae, and Henipaviruses. Methods: We modelled presence–absence data in spatially explicit binomial and zero-inflation binomial logistic regressions with and without autoregression. Presence data were extracted from published studies for the three EID groups. Various environmental and demographical rasters were used to explain the distribution of the EIDs. True Skill Statistic and deviance parameters were used to compare the accuracy of the different models. Results: For each group of viruses, we were able to identify and map areas at high risk of disease emergence based on the spatial distribution of the disease reservoirs and hosts of the three viral groups. Common influencing factors of disease emergence were climatic covariates (minimum temperature and rainfall) and human-induced land modifications. Conclusions: Using topographical, climatic, and previous disease outbreak reports, we can identify and predict future high-risk areas for disease emergence and their specific underlying human and environmental drivers. We suggest that such a predictive approach to EIDs should be carefully considered in the development of active surveillance systems for pathogen emergence and epidemics at local and global scales.

## 1. Introduction

Shifting geographical footprints of pathogens and/or infected hosts due to ecosystem disruption can lead to emerging infectious diseases (EIDs) [1], of which COVID-19 is a current example at the center of international attention. Infectious diseases of animal origins (or zoonoses) account for more than 70% of the emerging infectious diseases in recent decades [2,3]. With the onset of SARS-CoV-2, anticipating the emergence of new pathogens has become the major public health challenge of our time. The spatial dynamics of zoonotic diseases make it difficult to study and detect hotspots as they depend on the spatial distribution of mammalian hosts and reservoirs and their interactions with humans [4]. Studies show that disease emergence is closely linked to human-modified landscapes, such as fragmented peri-urban forests, which disrupt the human–animal–environment interface [5,6,7]. Therefore, ecological processes, landscape alterations, especially agricultural development, changes in water ecosystems, deforestation and reforestation, [4,8] and climate change [9] are the main drivers of EIDs.

Natural landscape attributes, such as elevation, and human-modified landscape factors, such as deforestation and agricultural expansions, influence the spatial extent of the hosts and reservoirs. For example, high elevation and water bodies could function as geographical barriers by preventing host movement [10], while rapid landscape changes can increase the likelihood of contact with a reservoir host and, thus, promote the emergence of micro-organisms previously unknown to humans [11]. Implementing a biogeographic approach to detect EID risks requires the integration of a spatial complexity using geographic information systems (GISs) the distribution of EIDs and its immediate environment [12]. Mathematical models help to integrate spatial data to measure the predictive risk of disease emergence. Recent work has shown that using a spatial Bayesian framework on species distribution modeling (SDM) produces more accurate results when dealing with limited and clumped data, as well as it takes into account random effects, thus, providing better results on landscape factors influencing risks [13,14]. Hierarchical Bayesian SDM allows for the observations to be interpreted as the result of ecological processes, such as climate change and human-altered landscapes.

Here, we have mapped and compared the predictive risk of the following three viral epidemics of infectious diseases transmissible to humans that are under surveillance: Filoviridae, including Ebola and Marburg viral diseases (EVD & MVD), Coronaviridae, such as SARS, MERS, and COVID-19, and Henipaviruses (Nipah & Hendra diseases) of the Paramyoviridae family. We are also identifying potential hotspots and quantifying the importance of environmental factors, such as climate and human-induced landscape, on the emergence of viral infectious diseases.

## 2. Methods

Using a Bayesian framework, we modeled the presence–absence data using a two-stage spatially explicit hierarchical logistic regression [15]. First, we modeled the potential presence of EID occurrence in each cell grid (local) of bioclimatic and population density variables using disease-level coefficients and a spatial random effect. Once the models were fitted, we compared the different models based on parameter summaries and model deviance.

### 2.1. Data

When considering the occurrence data for SDM, the most common biases arise from the assumption of perfect detection and stationary hosts. Disease occurrence depends on the spatial distribution of the disease reservoir and intermediate hosts. We used zero-inflation binomial models [16] to recognize the imperfect detection of the occurrence. Autocorrelation and non-stationarity of mammalian hosts were accounted for using intrinsic conditional autoregressive models (iCAR) to avoid overestimations of the spatial inference and prediction in the models. We extracted occurrence data on the global occurrences of Filoviridae, Coronaviridae, and Henipavirus human disease outbreaks over time from WHO archives and published studies (Supplementary Table S1). In cases where the origin of the outbreaks was unclear, we restricted the analysis down to the region or district of origin. Laboratory outbreaks, outbreaks resulting in asymptomatic diseases (Reston Ebola disease in the Philippines), and domestic (Hendra outbreaks in horses) and wildlife (Ebola in gorilla populations) outbreaks were also excluded. We excluded the recent SARS-CoV-2 outbreak, as the origin of the infection remains controversial. The analysis and coordinates of the suspected origin of SARS-CoV-2 are included in the Supplementary Materials. We geo-referenced the sites of origin and constructed spatial buffers of 10 km around the geographical coordinates. For each group of viruses, we generated 500 random spatial points in the spatial buffers to constitute presence points. Pseudo-absences were randomly generated in the spatial extents of the reservoirs and intermediate hosts of each virus group in a 1:2 ratio, leading to 1000 absence points.

### 2.2. Bioclimatic and Population Predictors

We extracted climatic and elevation covariates, such as monthly maximum and minimum temperatures, rainfall, and elevation, from global Bioclim data [17] at a spatial resolution of 2.5 min or about 4.5 km at the equator. We used Moderate Resolution Imaging Spectroradiometer (MODIS) Land Cover Type (MCD12Q1) data [18] and land-use changes included in human activities from the Global Human Modification of Terrestrial Systems dataset [19]. Finally, we used the Gridded Population of the World, (GPWv4) for the human population density raster [20]. The raster layers were resampled to a fixed resolution of 4.5 km and stacked to a raster brick. We obtained the geographical distribution and spatial extent of the primary hosts and reservoir mammals from the IUCN red list [21]; the list of mammals is included in Supplementary Materials.

### 2.3. Model Fitting and Model Prediction

The models were fitted using the R package “hsdm”, which uses a hierarchical Bayesian approach incorporating spatial dependency into the analysis by accounting for geographical clumping, which can be explained by biological (reservoir and host movement) or bioclimatic variables. In our study, we analyzed the data using hierarchical binomial and ZIB hierarchal SDM models with and without spatial autoregression. The ZIB models combine a binomial process for observability and a Bernoulli process for habitat suitability [22,23]. To model the spatial autocorrelation, we used a SDM with an intrinsic conditional autoregressive model (iCAR) [24]. The model is fitted using a Bayesian framework that allows the use of pre-validated predictors and the generation of parameter uncertainties. A mixture of topographical, climatic, landscape, and human-dependent predictors was used. The effect of a predictor was considered significant if it fell within a 95% confidence interval of the posterior distribution parameter. We used non-informative priors with a large variance of 10e6 (mean = 0), except for the spatial random effects, for which a weak informative prior: Uniform (min = 0, max = 10) was used. Two parallel MCMCs were run for each parameter and the convergence of the chains was checked visually using traceplots (Supplementary Information) and the Gelman and Ruben’s convergence diagnostics. High-risk areas or hotspots for each viral EID group are predicted using a maximum sensitivity + specificity threshold selection and the accuracy of the model was determined by True Skill Statistic (TSS) [25].

## 3. Results

### 3.1. Model Comparison

We used hierarchical SDM binomial, ZIB, binomial iCAR, and ZIB iCAR models to map the predictive risks of viral EIDs. To compare the models with respect to deviance, we constructed a geographical null model and later calculated the percentage of deviance explained by the null model. The spatial autoregression models performed better than their counterparts in the three groups (Table 1). In the Filoviridae prediction model, we found that 69% of the null deviance could be explained by the bioclimatic and population predictors using the ZIB model, which does not allow for an accurate identification of hotspots. In contrast, the inclusion of the random effects through iCAR allowed us to explain 100% of the null deviance, resulting in a perfect or saturated model. Similarly, ZIB models with an imperfect detection performed slightly better with Coronaviridae, with 74% and 100% of the null deviance explained without and with iCAR, respectively. However, with the Henipavirus EID models, the binomial iCAR model was slightly better, but we chose to summarize the ZIB with iCAR as the possibility of an imperfect detection of outbreak events remains a concern. In addition, the ZIB model was able to explain 71% of the null deviance using predictors for Henipavirus events, which is superior to the binomial model and facilitates model standardization and comparison. Spatial autocorrelation, MCMC traceplots, and TSS evolution for each model are available in the Supplementary Information.

### 3.2. Detection of EID Hotspots

**Figure 1:**
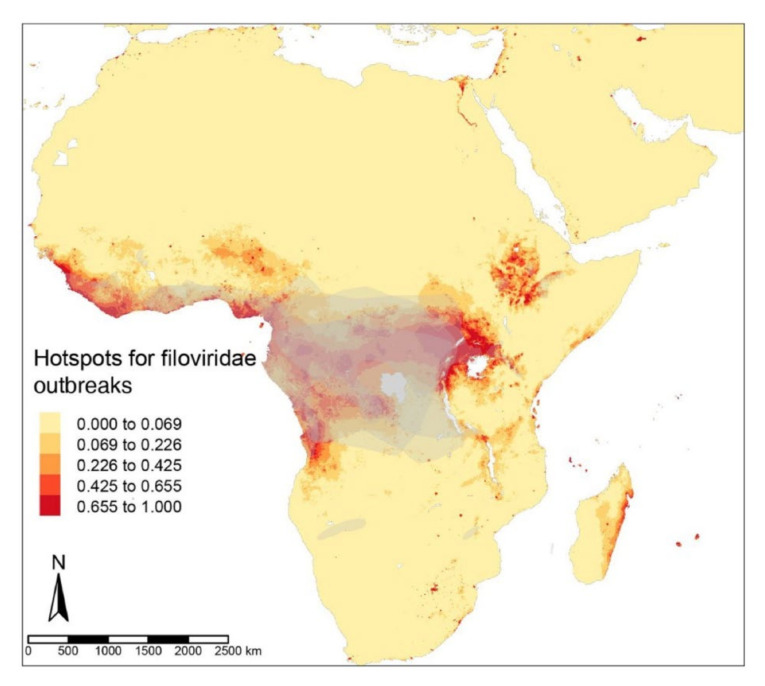
Predictive risks of Filoviridae disease emergence modeled from the ZIB iCAR model with a spatial distribution of the mammalian reservoirs in grey.

**Figure 2:**
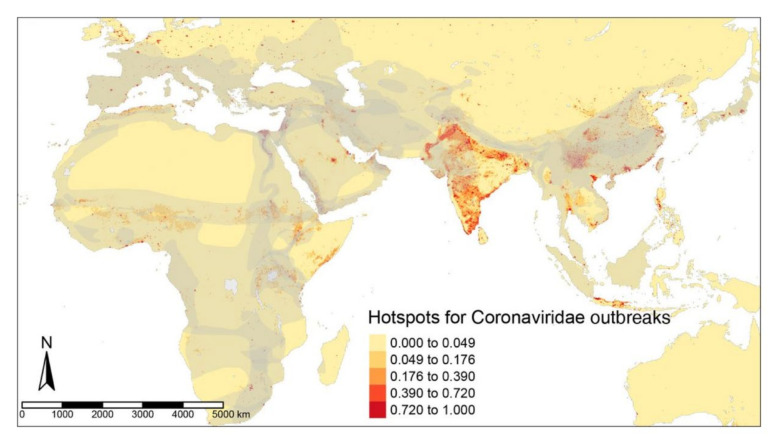
Predictive risks of Coronaviridae disease emergence modeled from the ZIB iCAR model with a spatial distribution of the mammalian reservoirs in grey.

**Figure 3:**
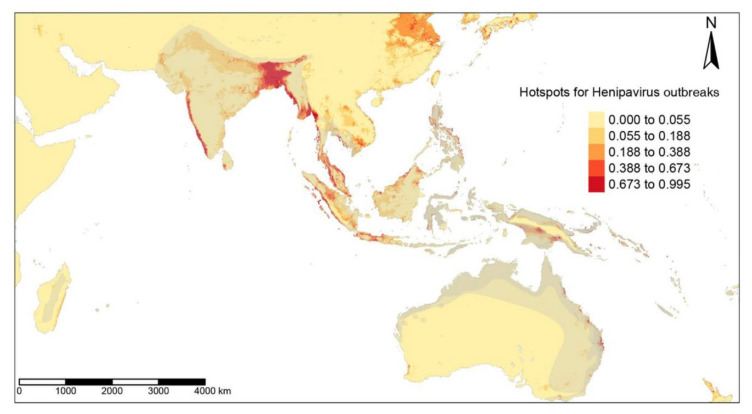
Predictive risks of Henipavirus disease emergence modeled by the ZIB iCAR model with a spatial distribution of the mammalian reservoirs in grey.

The hotspots for filovirus diseases, EVDs and MVDs, were found in the forest regions of Uganda, Southern Sudan, and eastern parts of the Democratic Republic of Congo, with smaller areas in West and Central Africa, as far as Angola (Figure 1). The ZIB iCAR model had a high TSS of 0.99 due to the addition of a spatial autoregulation. High-risk regions for EIDs caused by Coronaviridae predominate across the Indian subcontinent, with some areas in China and Southeast Asia (Figure 2) detected by the ZIB iCAR model with a high TTS of 1. Henipavirus disease hotspots are scattered along the west coast of India, in Bangladesh, along the coast in Malaysia, and in the smaller areas of the Indonesian Archipelago (Figure 3). The model had a high TSS of 0.99 and a probability threshold of 0.5.

### 3.3. Significant Environmental Predictors

We observed that minimum temperature [2.49; 95% CI 1.41 to 3.63] and rainfall [1.49; 95% CI 1.01 to 2.05] were the significant predictors of Filoviridae outbreaks in the ZIB iCAR model. As there were sufficient observations for MVD and EVD outbreak events, we ran separate the ZIB models with iCAR for each group of diseases to identify the significant predictors. The MVD model was found to have positive correlation with the minimum temperature [3.11; 2.03 to 4.40] and negative correlation with the maximum temperature [−2.21; −3.28 to −1.34], while the significant predictors of EVDs were found to be increasing minimum temperature [2.66; 1.14 to 3.91] and human-induced land cover changes. [1.50; 1.02 to 1.99]. We observed a significant influence of population densities [3.75; 2.15 to 5.43] and land cover changes [2.55; 1.48 to 3.69] on the distribution of hotspots for Coronaviridae events. The Nipah and Hendra virus outbreaks were negatively influenced by elevation [−8.84; −16.27 to −4.95] and positively influenced by increases in human-induced land changes [2.13; 1.41 to 2.88] and mean rainfall [2.43; 1.62 to 3.12]. The estimated quantiles of the MCMC chains for each variable are detailed in Table 2.

## 4. Discussion

Here, we show that ecological, climatic, and landscape factors could predict future hotspots of human viral disease emergence on a global scale and could, thus, serve as a basis for surveillance and early warning systems. For the three groups of viral diseases studied, we were able to map areas at high risk of disease emergence based on the spatial distribution of disease reservoirs and hosts, as well as WHO data on the distribution of each disease. We found that human-related factors, particularly the impact of population growth on human-modified landscapes, were a common predictor of disease emergence. Filoviridae and Henipavirus outbreaks were also linked to rainfall, while Filoviridae and Coronaviridae emergences were favored by increases in the minimum nighttime temperatures. In addition, for Filoviridae, we noted the potential involvement of “unknown” variables (variables not used in this study). In Africa, these variables could relate to human behaviors, such as bushmeat consumption, that are often associated with EVD outbreaks [26], biodiversity loss, or even other bioclimatic covariates. Interestingly, coronavirus diseases are the only ones to be positively impacted by human population densities. Similarly, the hotspots of Henipaviruses depended on areas of low elevation and low rainfall.

Recent research has shown that the increased surface temperature and unpredictable seasonal rainfall due to climate change have an indirect effect on disease emergence through sudden ecological changes of their reservoir, loss of biodiversity, and migration of small mammal hosts [27,28]. For example, minimum temperature is the limiting factor for parasite development and vector distribution in malaria transmission [29] and other vector-borne disease epidemics, such as the Crimean Congo Hemorrhagic Fever and Zika [5,18]. Unfortunately, the research outside of vector-borne diseases is limited. However, this direct spatial dependence of disease emergence on minimum temperatures is worrying. Indeed, with climate change increasing the nighttime minimum temperatures and lengthening the frost-free season in most mid- and high latitude regions [30], there could be a potentially increase the latitudinal extent of infectious disease emergences.

We also found that low elevation and high rainfall have a significant influence on the distribution of Henipavirus outbreaks. Consistent with our results, studies have hypothesized that the emergence of Nipah in the lower Gangetic plains and low-lying marshes could be attributed to flooding, which leads to the destruction of mammalian habitats [31]. Rapid changes in ecological habitats due to human land-use changes lead to the starvation and migration of fruit bat species (Family *Pteropodidae*), reservoirs of Nipah virus, with contamination of fruit trees near human habitations and increased exposure to the pathogen [31,32,33]. Our results support this hypothesis of the increased risk of Nipah outbreaks associated with lowland plains, flooding, and rapid human-induced habitat changes.

EVDs and coronaviral diseases have also been found to be associated with human-modified landscapes. EVDs have long been linked to landscape alterations, such as deforestation, mining, population growth, and land fragmentation [34,35,36]. Our results show that EVD outbreaks are not directly related to population densities, contrary to a recent study [36], but rather to the effects of population increases on human-modified landscapes, such as urbanization, deforestation, mining, and hunting. In contrast, population density was significantly related to coronavirus hotspots. Whether high population density leads to observer bias and, thus, to increased reporting of outbreaks needs to be examined in detail. The report of a SARS-like pneumonia in 2012 in miners in Tongguan, Mojiang [37] raises the issue of potentially unreported sporadic outbreaks in regions with limited populations. Studies show that the emergence of coronaviral diseases, such as SARS [38] and MERS [39], is directly related to exposure to body fluids from mammals raised in confined spaces for bushmeat and recreational activities, respectively. “Wild flavor” bushmeat restaurants and markets are often located in densely populated cites, where the demand for exotic proteins is high [26,40], and cases are, therefore, more likely to be reported in densely populated areas. In the case of MERS, there is an increase in reporting in large cities as camel owners seek treatment for respiratory distress in tertiary hospitals located in large cities and are, therefore, more likely to report cases [39]. The effect of population density is, however, crucial in the spread of epidemics and, therefore, remains an important factor in the detection of hotspots and active surveillance.

We suggest here the urgent need of alternatives to rapid land-use changes, such as deforestation, land fragmentation for agriculture and livestock, and changes in the cultural practices of bushmeat consumption. More importantly, the results highlight the major impact of increasing populations and human activities on land alterations and ecological changes, as well as the dependence of viral disease emergences on bioclimatic changes (minimum temperature, rainfall, low elevation, and flooded areas) at the global scale. We show the potential of using climatic, topographic, and population data to identify and predict areas at high risk of disease emergences. Although our study focused on three viral diseases of concern, we suggest that such biogeographic approach to predicting disease emergences should be considered and tested for other diseases under surveillance in a global active surveillance context.

## 5. Conclusions

Landscape (deforestation, urbanization, elevation, flooded areas, etc.), climatic (rainfall, temperature, etc.), and epidemic data were used in combination to estimate the potential role of human-induced landscape and climate changes on the emergence of Filoviridae, Coronaviridae, and Henipaviruses at a global scale. Such predictive approach for identifying regions at high risk of disease emergences should be considered for monitoring local and global pathogen emergences and for the identifying potential future epidemics. By recognizing the influence of predictive environmental factors on EIDs and adopting a predictive approach to disease emergence, unprecedented EID outbreaks could be made predictable.

## Figures and Tables

**Table 1:** Model deviance with the percentage of deviance explained by a predictor variable.

Model	Deviance	Percentage of Deviance Explained
*Filoviridae*		
NULL	1891.41	0
Binomial	839.24	65
ZIB	764.38	69
Binomial.iCAR	268.52	100
ZIB.iCAR	267.14	100
*Coronaviridae*		
*NULL*	1889.18	0
Binomial	629.11	69
ZIB	548.47	74
Binomial.iCAR	63.09	100
ZIB.iCAR	67.01	100
*Henipavirus*		
NULL	1862.74	0
Binomial	635.28	70
ZIB	627.49	71
Binomial.iCAR	113.44	100
ZIB.iCAR	116.76	100

**Table 2:** Quantiles for each variable contributing to the ZIB iCAR model predictions. The significant variables are shown in bold.

Variable	2.50%	25%	50%	75%	97.50%
**ZIB iCAR Filoviridae outbreak model**					
Min. temperature	**1.41**	**2.14**	**2.49**	**2.87**	**3.62**
Max. temperature	−2.13	−1.55	−1.26	−0.98	−0.48
Mean precipitation	**1.01**	**1.31**	**1.49**	**1.67**	**2.05**
Land cover	−1.04	−0.77	−0.62	−0.45	−0.16
Elevation	0.59	1.06	1.3	1.57	2.14
Land-use changes	0.75	1	1.15	1.33	1.64
Population density	0.48	1	1.33	1.7	2.5
**ZIB iCAR Coronaviridae outbreak model**					
Min. temperature	−1.09	2.75	4.35	5.92	9.36
Max. temperature	−5.07	−2.42	−0.97	0.34	3.56
Mean precipitation	−3.21	−2.33	−1.89	−1.37	−0.38
Land cover	−1.14	−0.56	−0.27	0.02	0.64
Elevation	0.95	1.77	2.2	2.63	3.51
Land-use changes	**1.47**	**2.15**	**2.5**	**2.9**	**3.69**
Population density	**2.15**	**3.19**	**3.75**	**4.31**	**5.43**
**ZIB iCAR Henipavirus outbreak model**					
Min. temperature	−6.26	−4.49	−3.67	−2.88	−0.66
Max. temperature	−0.02	1.67	2.35	3.1	4.53
Mean precipitation	**1.62**	**2.13**	**2.43**	**2.71**	**3.12**
Land cover	−0.23	0.11	0.29	0.47	0.83
Elevation	**−16.27**	**−10.32**	**−8.84**	**−7.6**	**−4.95**
Land-use changes	**1.41**	**1.89**	**2.13**	**2.39**	**2.88**
Population density	−0.31	−0.1	0	0.11	0.3

## Data Availability

All data and R code for the models used in this manuscript can be accessed at https://github.com/soushie13/Bayes_sdm (accessed on 29 June 2022).

## Supplementary Materials

The following supporting information can be downloaded at: https://www.mdpi.com/article/10.3390/tropicalmed7070124/s1, Figure S1: MCMC traceplots; Figure S2: True Skill Statistic (TSS) plots; Figure S3: ZIB iCAR random effects; Figure S4: Significant hotspots based on TSS; Table S1: Reservoirs of diseases analyzed.

## References

[1] Ogden N., AbdelMalik P., Pulliam J. (2017). Emerging Infectious Diseases: Prediction and Detection. Can. Commun. Dis. Rep..

[2] Jones K.E., Patel N.G., Levy M.A., Storeygard A., Balk D., Gittleman J.L., Daszak P. (2008). Global Trends in Emerging Infectious Diseases. Nature.

[3] Taylor L.H., Latham S.M., Woolhouse M.E.J. (2001). Risk Factors for Human Disease Emergence. Philos. Trans. R. Soc. B Biol. Sci..

[4] Meentemeyer R.K., Haas S.E., Václavík T. (2012). Landscape Epidemiology of Emerging Infectious Diseases in Natural and Human-Altered Ecosystems. Annu. Rev. Phytopathol..

[5] Myers S.S., Gaffikin L., Golden C.D., Ostfeld R.S., Redford K.H., Ricketts T.H., Turner W.R., Osofsky S.A. (2013). Human Health Impacts of Ecosystem Alteration. Proc. Natl. Acad. Sci. USA.

[6] Gibb R., Redding D.W., Chin K.Q., Donnelly C.A., Blackburn T.M., Newbold T., Jones K.E. (2020). Zoonotic Host Diversity Increases in Human-Dominated Ecosystems. Nature.

[7] Daszak P., Olival K.J., Li H. (2020). A Strategy to Prevent Future Epidemics Similar to the 2019-NCoV Outbreak. Biosaf. Health.

[8] Patz J.A., Daszak P., Tabor G.M., Aguirre A.A., Pearl M., Epstein J., Wolfe N.D., Kilpatrick A.M., Foufopoulos J., Molyneux D. (2004). Unhealthy Landscapes: Policy Recommendations on Land Use Change and Infectious Disease Emergence. Environ. Health Perspect..

[9] Patz J.A., Olson S.H., Uejio C.K., Gibbs H.K. (2008). Disease Emergence from Global Climate and Land Use Change. Med. Clin. North Am..

[10] Smith D.L., Lucey B., Waller L.A., Childs J.E., Real L.A. (2002). Predicting the Spatial Dynamics of Rabies Epidemics on Heterogeneous Landscapes. Proc. Natl. Acad. Sci. USA.

[11] Fenollar F., Mediannikov O. (2018). Emerging Infectious Diseases in Africa in the 21st Century. New Microbes New Infect..

[12] Biek R., Real L.A. (2010). The Landscape Genetics of Infectious Disease Emergence and Spread. Mol. Ecol..

[13] Jagadesh S., Combe M., Couppié P., Le Turnier P., Epelboin L., Nacher M., Gozlan R.E. (2019). Emerging Human Infectious Diseases of Aquatic Origin: A Comparative Biogeographic Approach Using Bayesian Spatial Modelling. Int. J. Health Geogr..

[14] Redding D.W., Lucas T.C.D., Blackburn T.M., Jones K.E. (2017). Evaluating Bayesian Spatial Methods for Modelling Species Distributions with Clumped and Restricted Occurrence Data. PLoS ONE.

[15] Gelfand A.E., Holder M., Latimer A., Lewis P.O., Rebelo A.G., Silander J.A., Wu S. (2006). Explaining Species Distribution Patterns through Hierarchical Modeling. Bayesian Anal..

[16] Latimer A.M., Wu S., Gelfand A.E., Silander J.A. (2006). Building Statistical Models To Analyze Species Distributions. Ecol. Appl..

[17] Fick S.E., Hijmans R.J. (2017). WorldClim 2: New 1-Km Spatial Resolution Climate Surfaces for Global Land Areas. Int. J. Climatol..

[18] Broxton P.D., Zeng X., Sulla-Menashe D., Troch P.A. (2014). A Global Land Cover Climatology Using MODIS Data. J. Appl. Meteorol. Climatol..

[19] Kennedy C.M., Oakleaf J.R., Theobald D.M., Baruch-Mordo S., Kiesecker J. (2020). Global Human Modification of Terrestrial Systems.

[20] Center for International Earth Science Information Network (2018). Gridded Population of the World, Version 4 (GPWv4): Population Density, Revision 11.

[21] Isberg S., Balaguera-Reina S.A., Ross J.P. (2017). The IUCN Red List of Threatened Species.

[22] Plumptre A.J., Nixon S., Kujirakwinja D.K., Vieilledent G., Critchlow R., Williamson E.A., Nishuli R., Kirkby A.E., Hall J.S. (2016). Catastrophic Decline of World’s Largest Primate: 80% Loss of Grauer’s Gorilla (Gorilla Beringei Graueri) Population Justifies Critically Endangered Status. PLoS ONE.

[23] Wilson A.M., Jetz W. (2016). Remotely Sensed High-Resolution Global Cloud Dynamics for Predicting Ecosystem and Biodiversity Distributions. PLoS Biol..

[24] Besag J. (1974). Spatial Interaction and the Statistical Analysis of Lattice Systems. J. R. Stat. Soc. Ser. B Methodol..

[25] Liu C., White M., Newell G. (2011). Measuring and Comparing the Accuracy of Species Distribution Models with Presence-Absence Data. Ecography.

[26] Wolfe N.D., Daszak P., Kilpatrick A.M., Burke D.S. (2005). Bushmeat Hunting, Deforestation, and Prediction of Zoonotic Disease Emergence. Emerging Infectious Diseases.

[27] García F.C., Bestiona E., Warfielda R., Yvon-Durochera G. (2018). Changes in Temperature Alter the Relationship between Biodiversity and Ecosystem Functioning. Proc. Natl. Acad. Sci. USA.

[28] El-Sayed A., Kamel M. (2020). Climatic Changes and Their Role in Emergence and Re-Emergence of Diseases. Environ. Sci. Pollut. Res. Int..

[29] Patz J.A., Olson S.H. (2006). Malaria Risk and Temperature: Influences from Global Climate Change and Local Land Use Practices. Proc. Natl. Acad. Sci. USA..

[30] Folland C.K., Karl T.R., Salinger M.J. (2002). Observed Climate Variability and Change. Weather.

[31] Ambat A.S., Zubair S.M., Prasad N., Pundir P., Rajwar E., Patil D.S., Mangad P. (2019). Nipah Virus: A Review on Epidemiological Characteristics and Outbreaks to Inform Public Health Decision Making. J. Infect. Public Health.

[32] Royce K., Fu F. (2020). Mathematically Modeling Spillovers of an Emerging Infectious Zoonosis with an Intermediate Host. PLoS ONE.

[33] Epstein J.H., Field H.E., Luby S., Pulliam J.R., Daszak P. (2006). Nipah Virus: Impact, Origins, and Causes of Emergence. Curr. Infect. Dis. Rep..

[34] Olivero J., Fa J.E., Real R., Márquez A.L., Farfán M.A., Vargas J.M., Gaveau D., Salim M.A., Park D., Suter J. (2017). Recent Loss of Closed Forests Is Associated with Ebola Virus Disease Outbreaks. Sci. Rep..

[35] Castillo-Chavez C., Curtiss R., Daszak P., Levin S.A., Patterson-Lomba O., Perrings C., Poste G., Towers S. (2015). Beyond Ebola: Lessons to Mitigate Future Pandemics. Lancet Glob. Health.

[36] Redding D.W., Atkinson P.M., Cunningham A.A., Lo Iacono G., Moses L.M., Wood J.L.N., Jones K.E. (2019). Impacts of Environmental and Socio-Economic Factors on Emergence and Epidemic Potential of Ebola in Africa. Nat. Commun..

[37] Rahalkar M.C., Bahulikar R.A. (2020). Lethal Pneumonia Cases in Mojiang Miners (2012) and the Mineshaft Could Provide Important Clues to the Origin of SARS-CoV-2. Front. Public Health.

[38] Shi Z., Hu Z. (2008). A Review of Studies on Animal Reservoirs of the SARS Coronavirus. Virus Res..

[39] (2015). Emhj. An Outbreak of Middle East Respiratory Syndrome (MERS) due to Coronavirus in Al-Ahssa Region, Saudi Arabia. http://www.emro.who.int/emhj-volume-22-2016/volume-22-issue-7/an-outbreak-of-middle-east-respiratory-syndrome-mers-due-to-coronavirus-in-al-ahssa-region-saudi-arabia-2015.html.

[40] Lee T.M., Sigouin A., Pinedo-Vasquez M., Nasi R. (2020). The Harvest of Tropical Wildlife for Bushmeat and Traditional Medicine. Annu. Rev. Environ. Resour..

