# Supplementary material for: Human-Altered Landscapes and Climate to Predict Human Infectious Disease Hotspots
Source: Trop Med Infect Dis. 2022 Jul 1;7(7):124. doi: 10.3390/tropicalmed7070124 (PMC9325272; doi:10.3390/tropicalmed7070124)

Supplementary information for

Identifying hotspots in the distribution of human infectious diseases using a Bayesian framework: A lead to drivers, prevention, and surveillance of disease emergence

This PDF contains:

- MCMC traceplots: pages 2-46
- MCMC model summaries: pages 47- 63
- True Skill Statistic (TSS) plots: pages 64-66
- ZIB iCAR random effects: pages 67-69
- Significant hotspots based on TSS: pages 70-72
- Table1: Reservoirs of diseases analyzed: pages 73 - 75
- Analysis of Coroviridae outbreak distribution with SARS CoV 2 origins: pages 76 - 84

Figure S1: MCMC traceplots

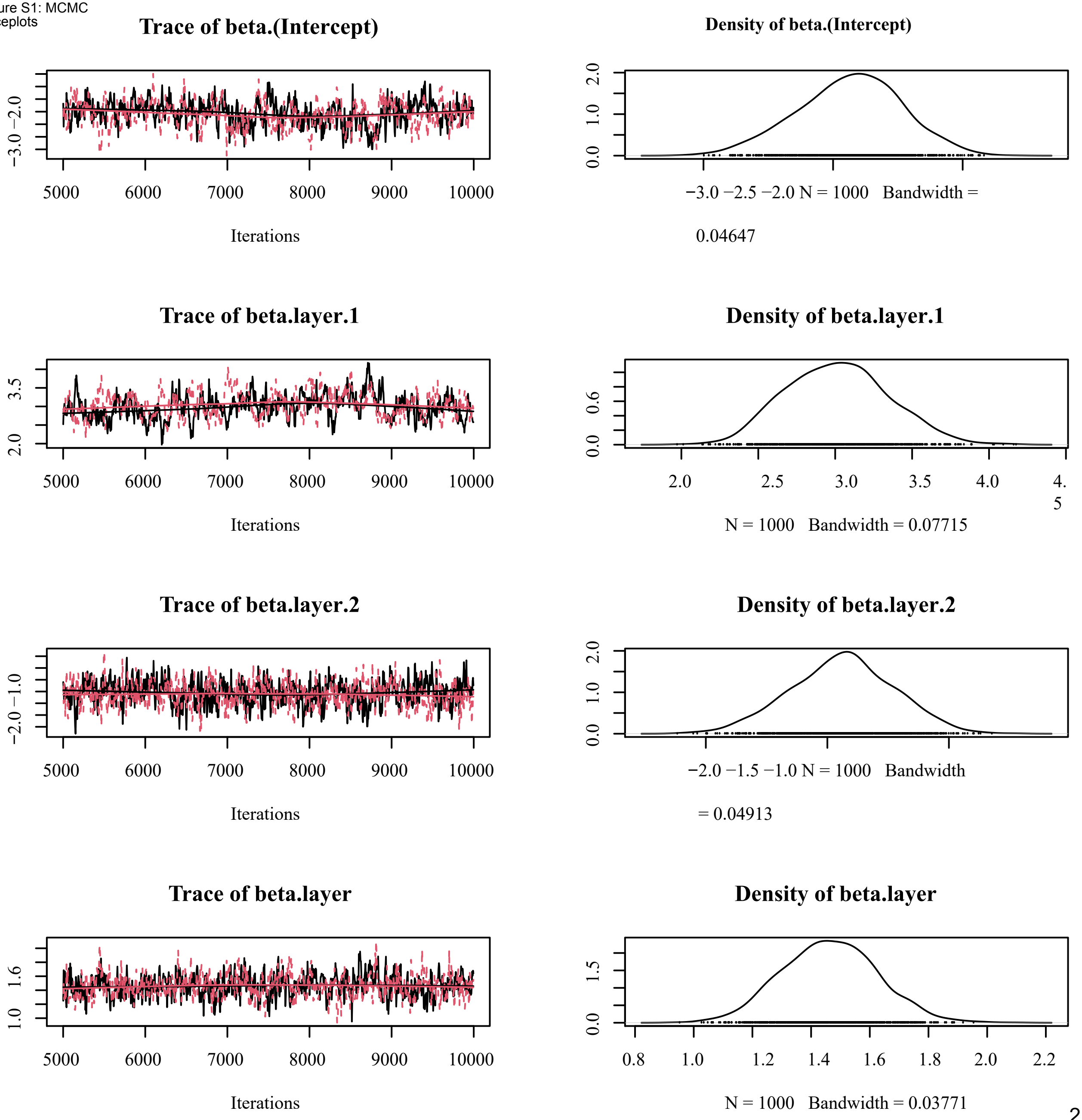

Filoviridae Binomial mcmc traceplot

Figure S1: MCMC traceplots

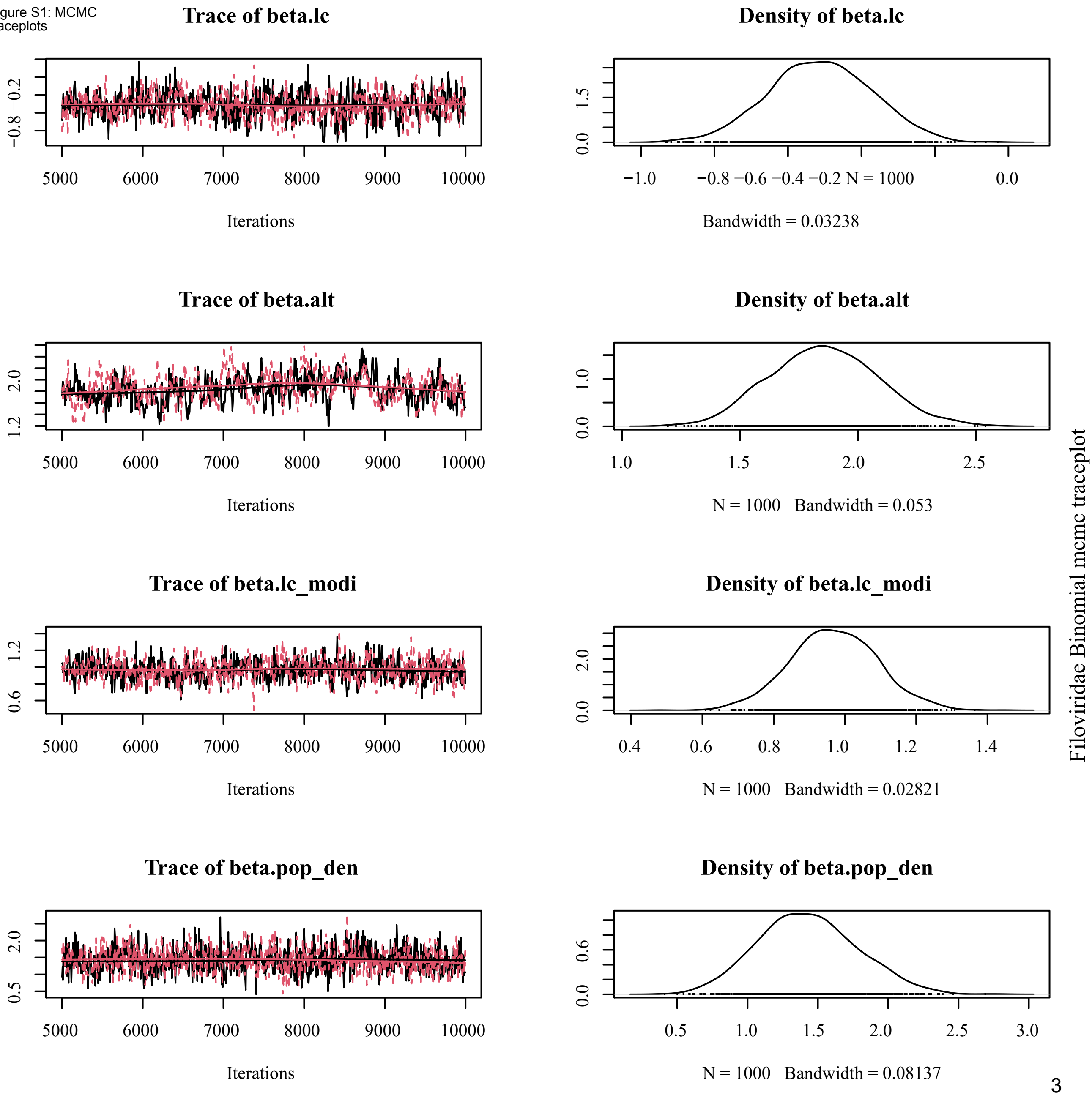

Figure S1: MCMC traceplots

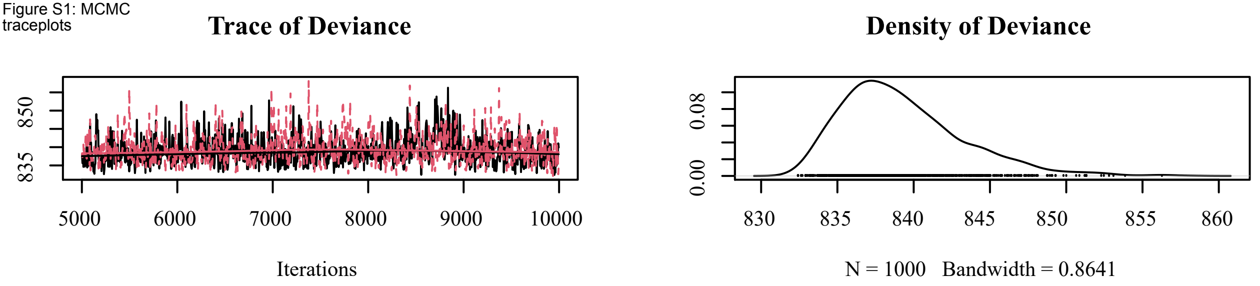

Filoviridae Binomial mcmc traceplot

Figure S1: MCMC traceplots

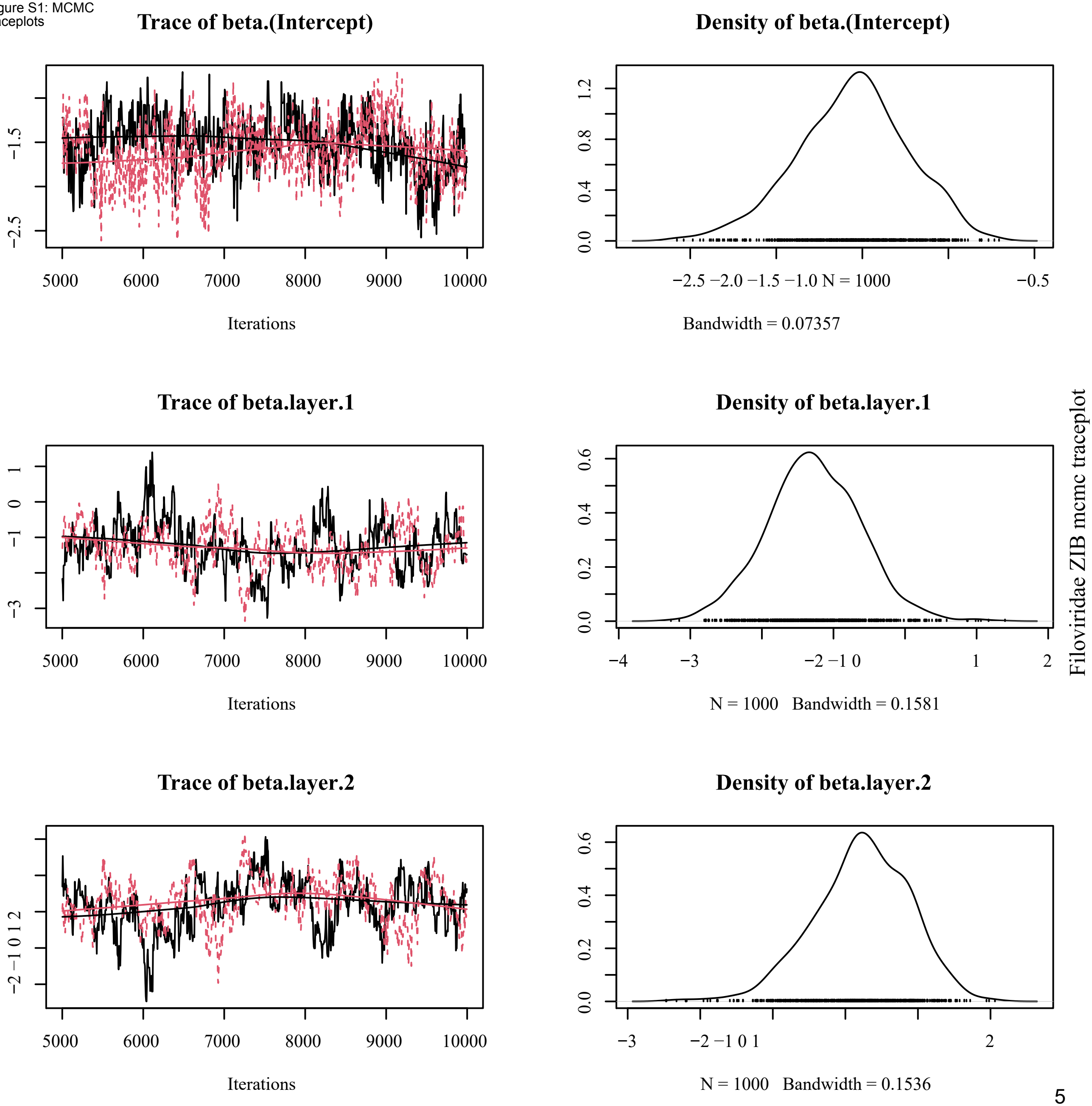

Figure S1: MCMC traceplots

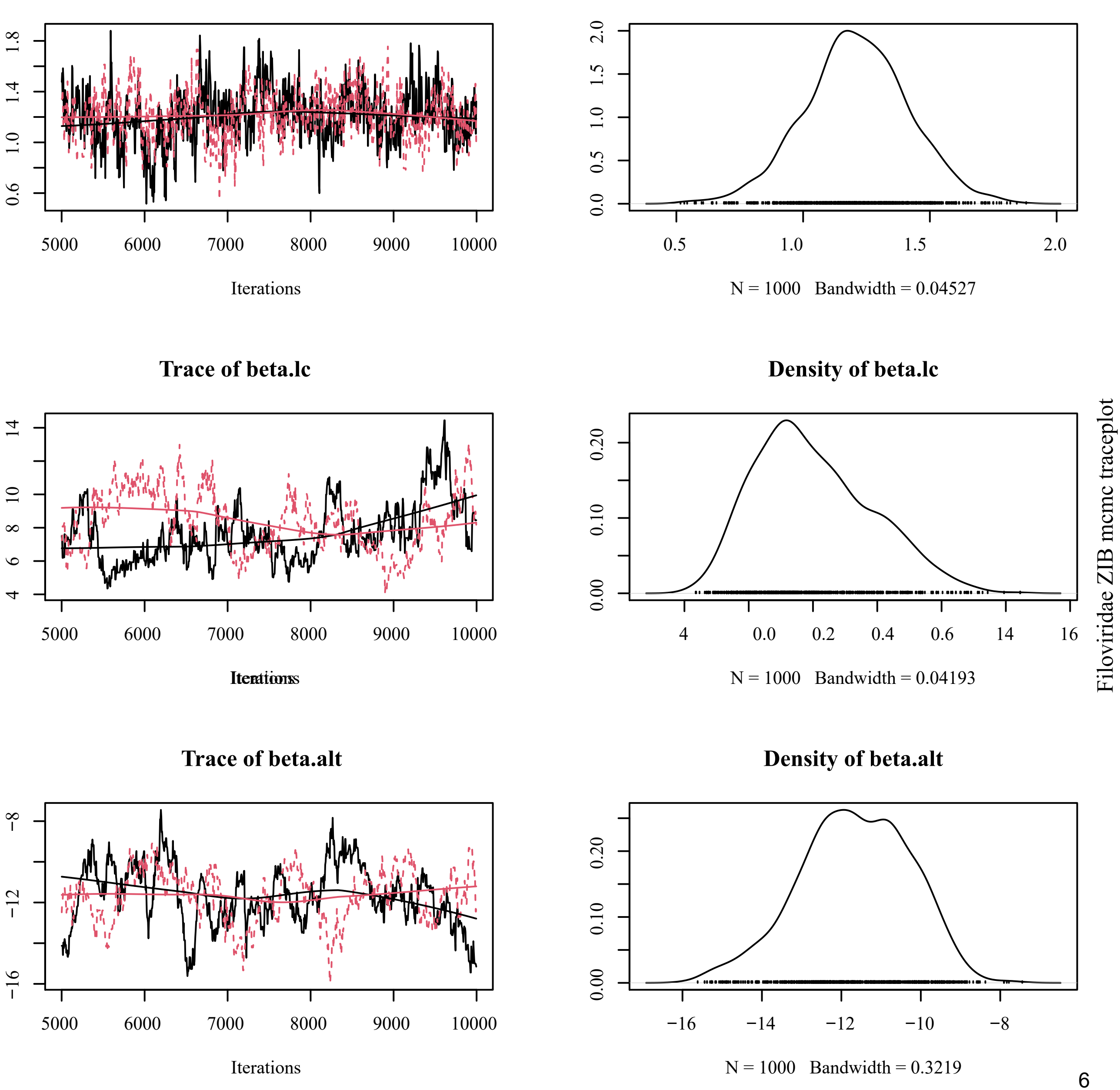

Figure S1: MCMC traceplots

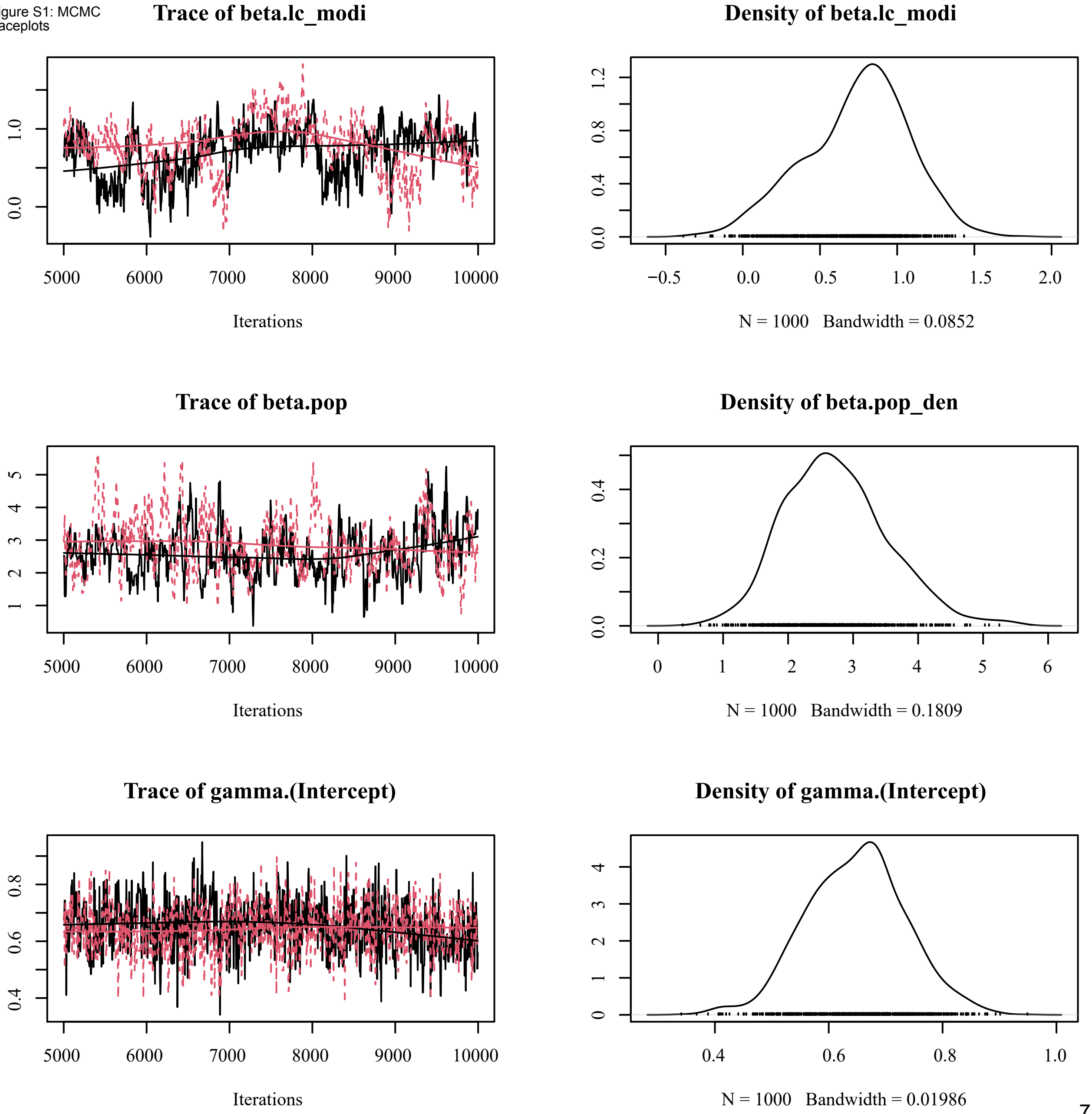

Filoviridae ZIB mcmc traceplot

Figure S1: MCMC traceplots

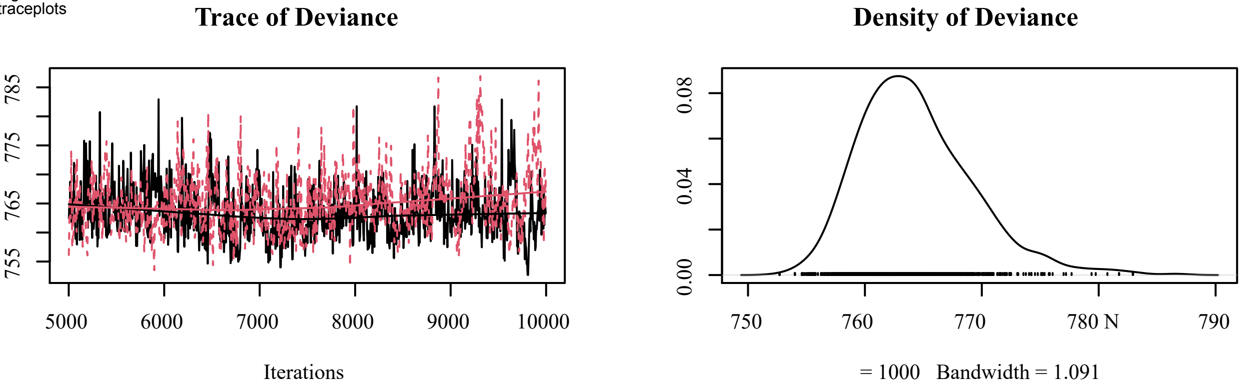

Filoviridae ZIB mcmc traceplot

Figure S1: MCMC traceplots

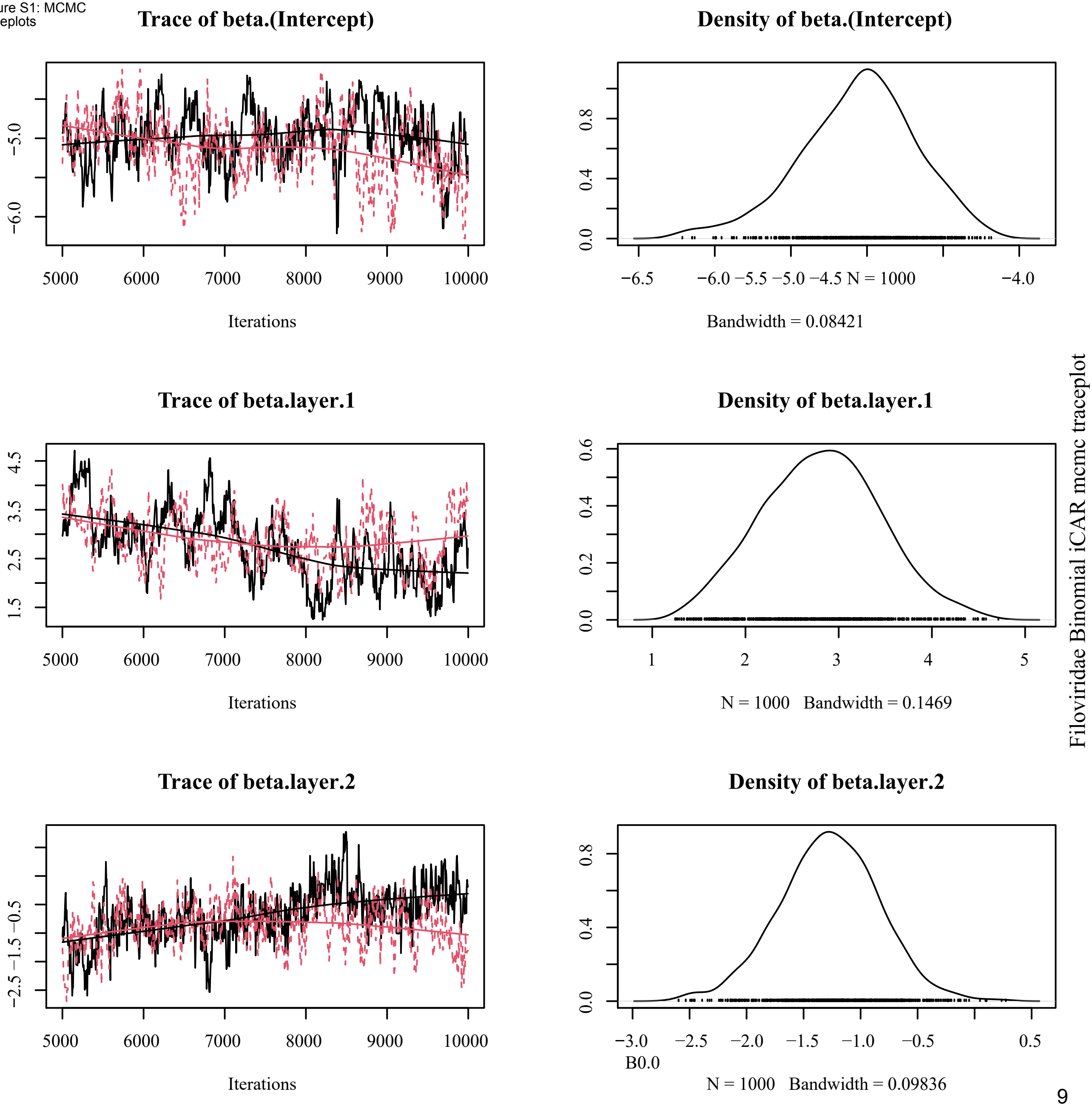

Figure S1: MCMC traceplots

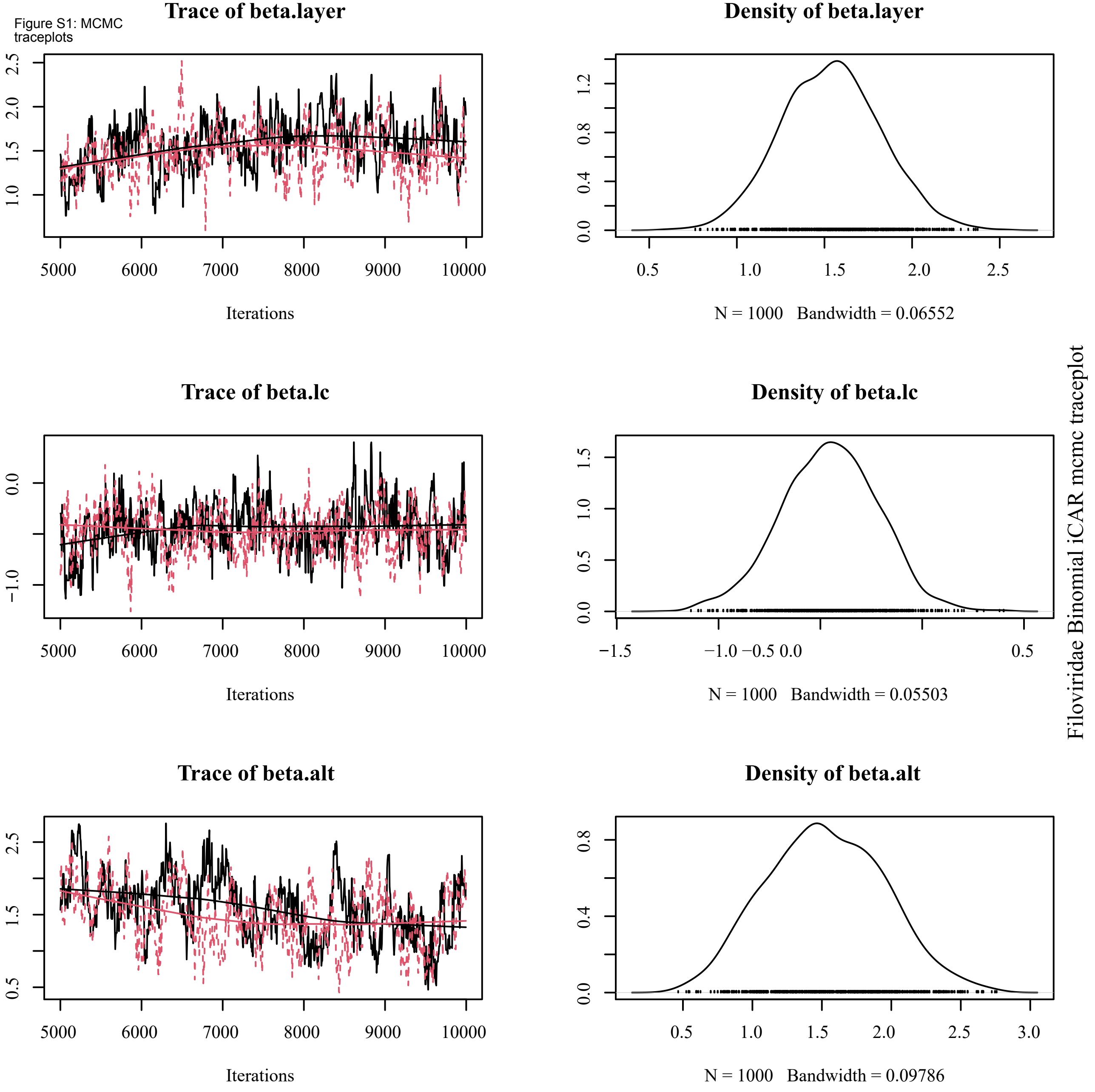

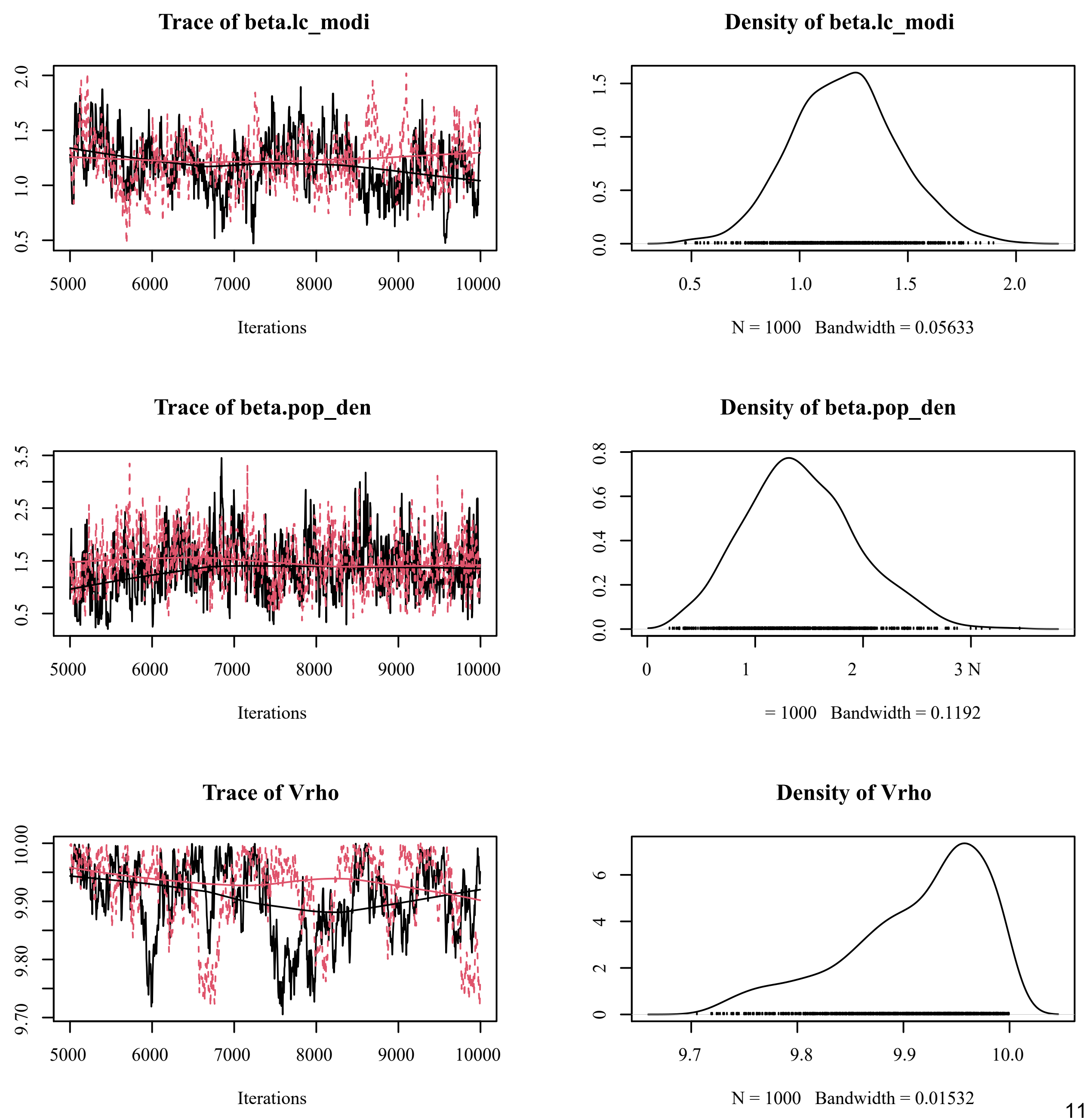

Filoviridae Binomial iCAR mcmc traceplot

Figure S1: MCMC traceplots

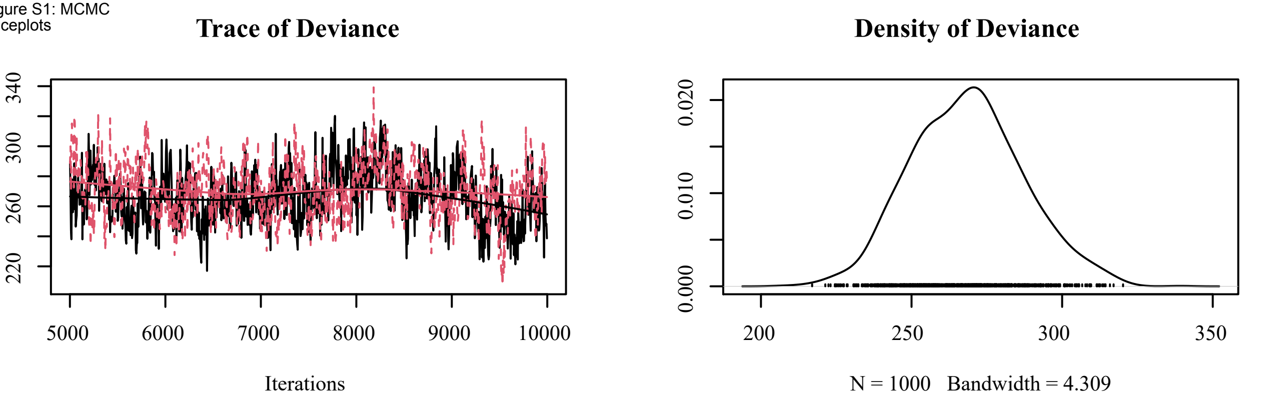

Filoviridae Binomial iCAR mcmc traceplot

Figure S1: MCMC traceplots

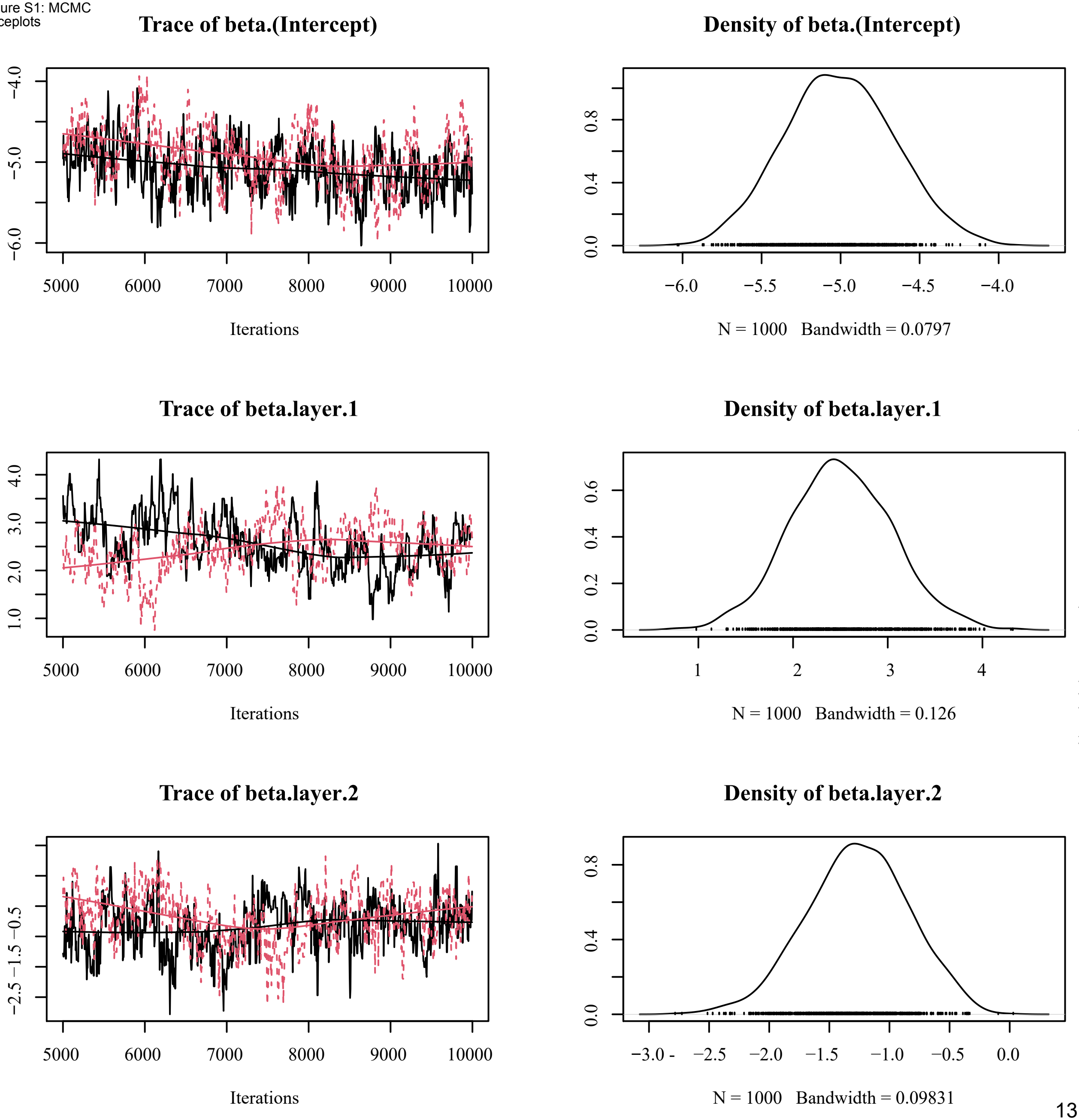

Filoviridae ZIB iCAR mcmc traceplot

Figure S1: MCMC traceplots

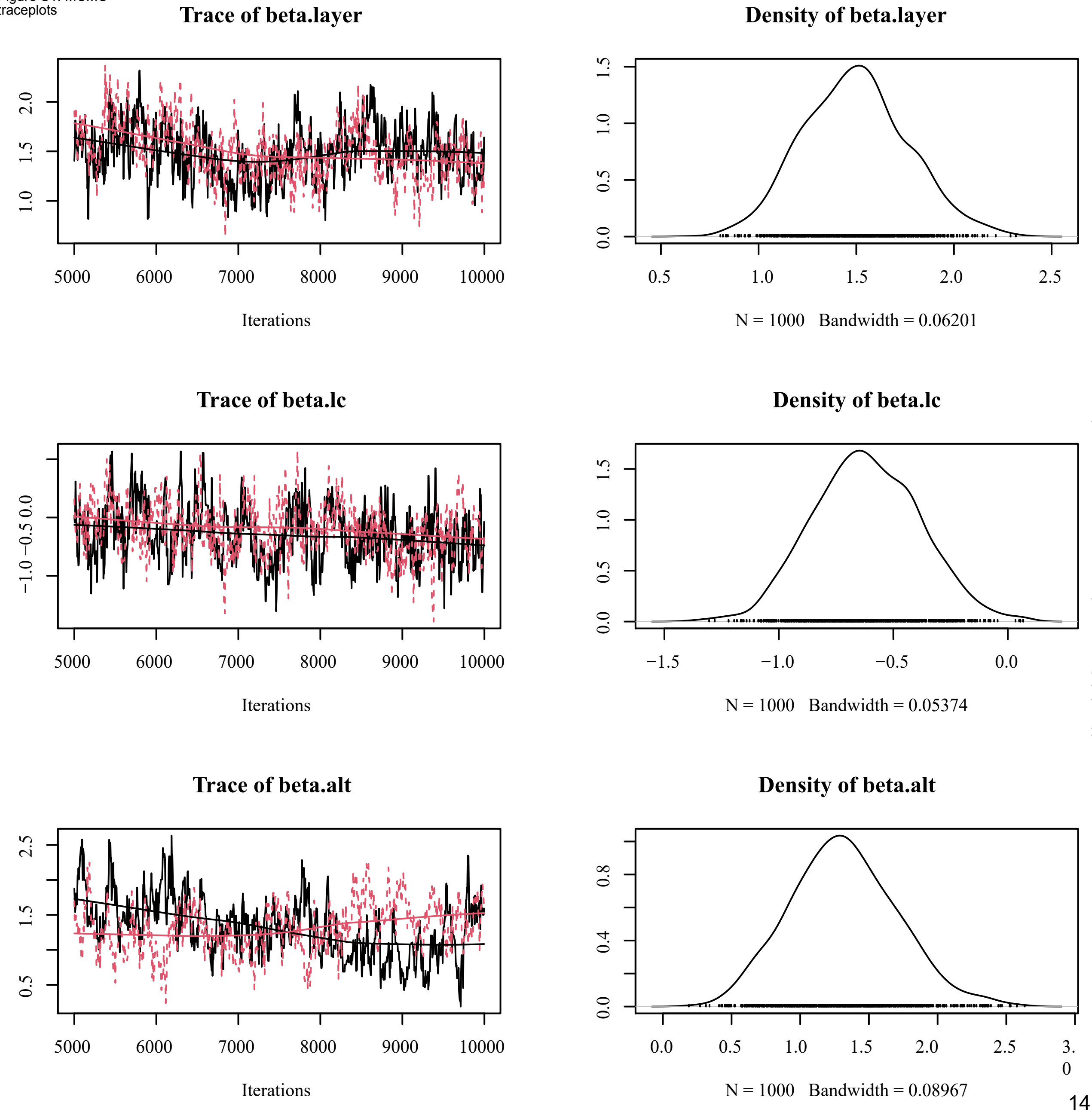

Filoviridae ZIB iCAR mcmc traceplot

Figure S1: MCMC traceplots

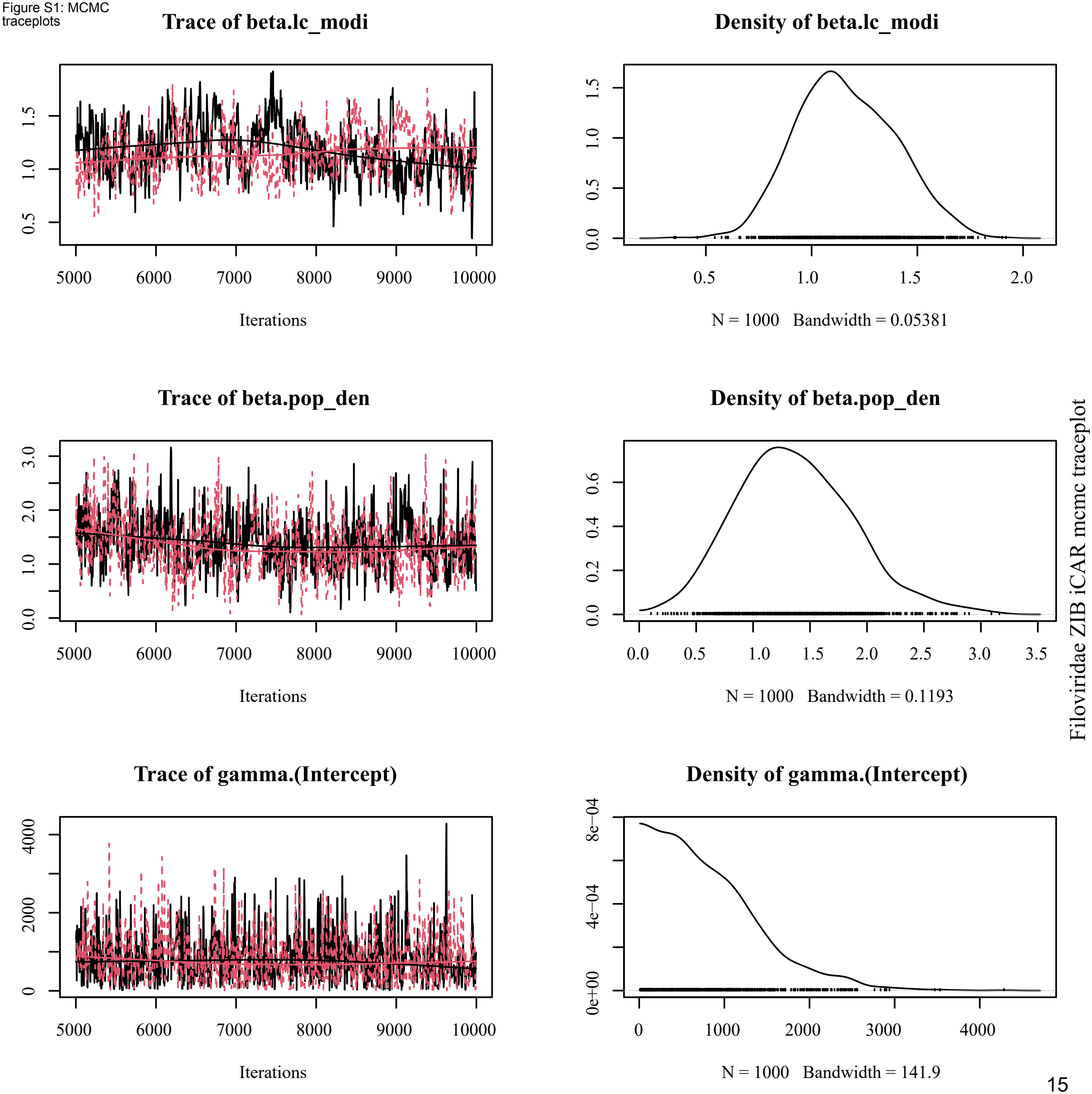

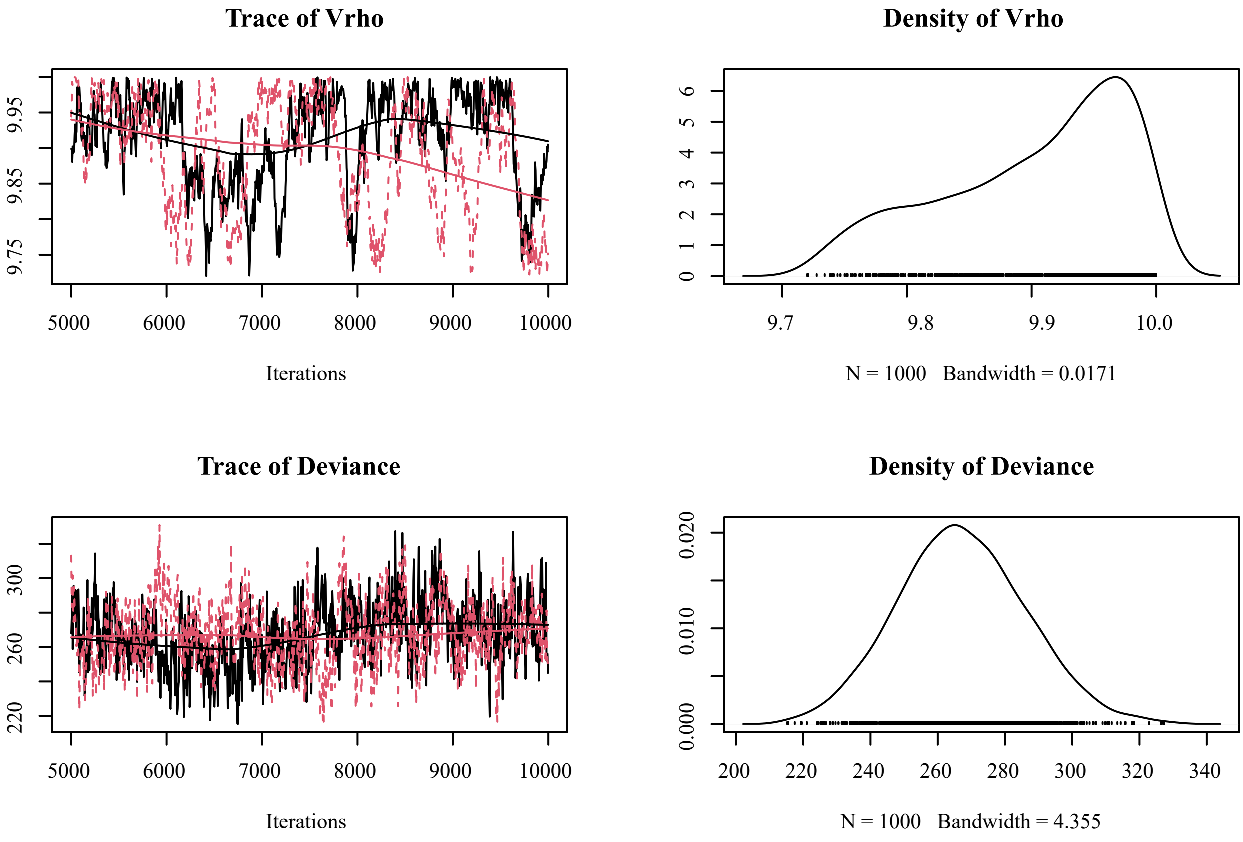

Filoviridae ZIB iCAR mcmc traceplot

Figure S1: MCMC traceplots

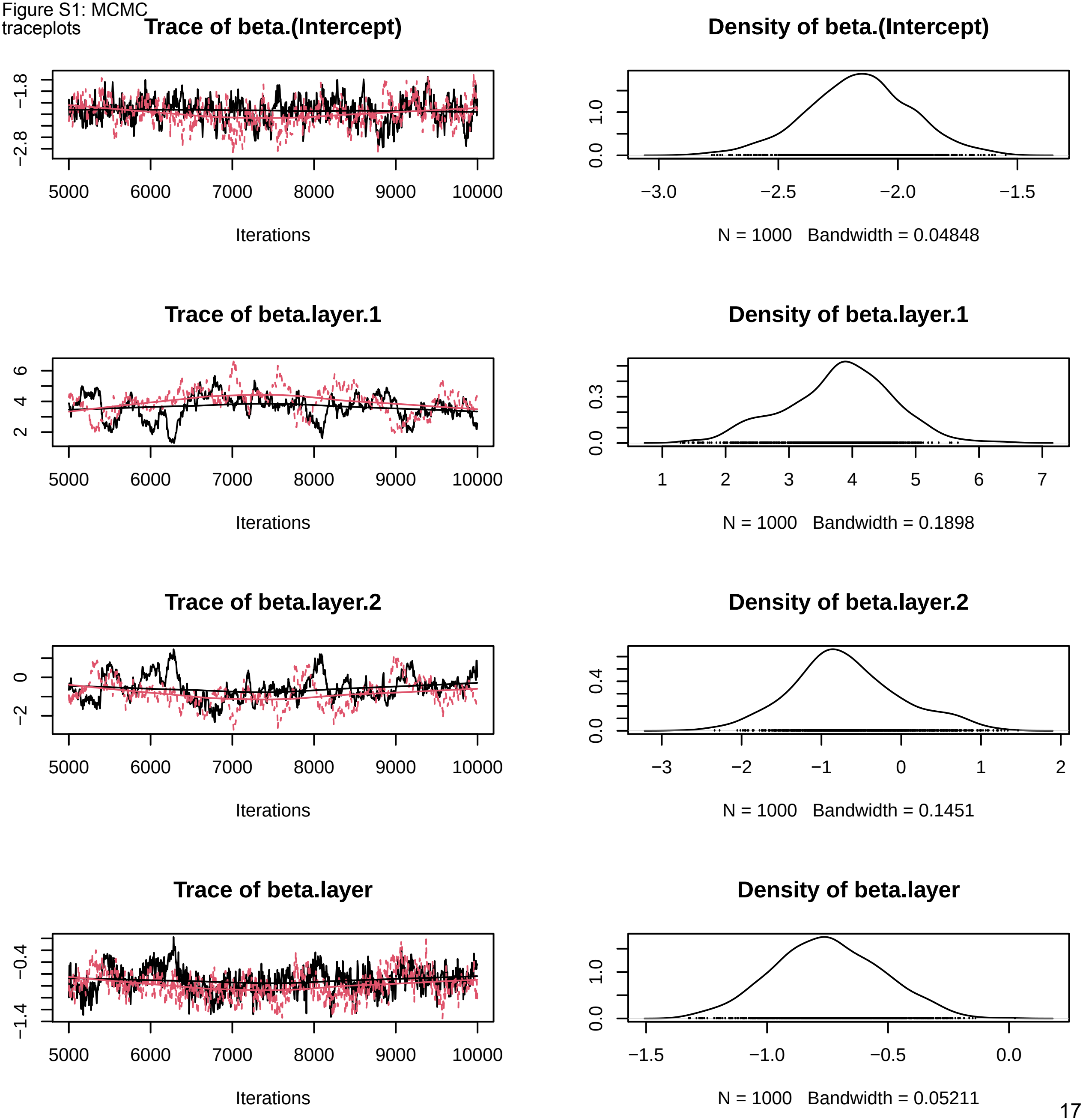

Coronaviridae Binomial mcmc traceplot

Figure S1: MCMC traceplots

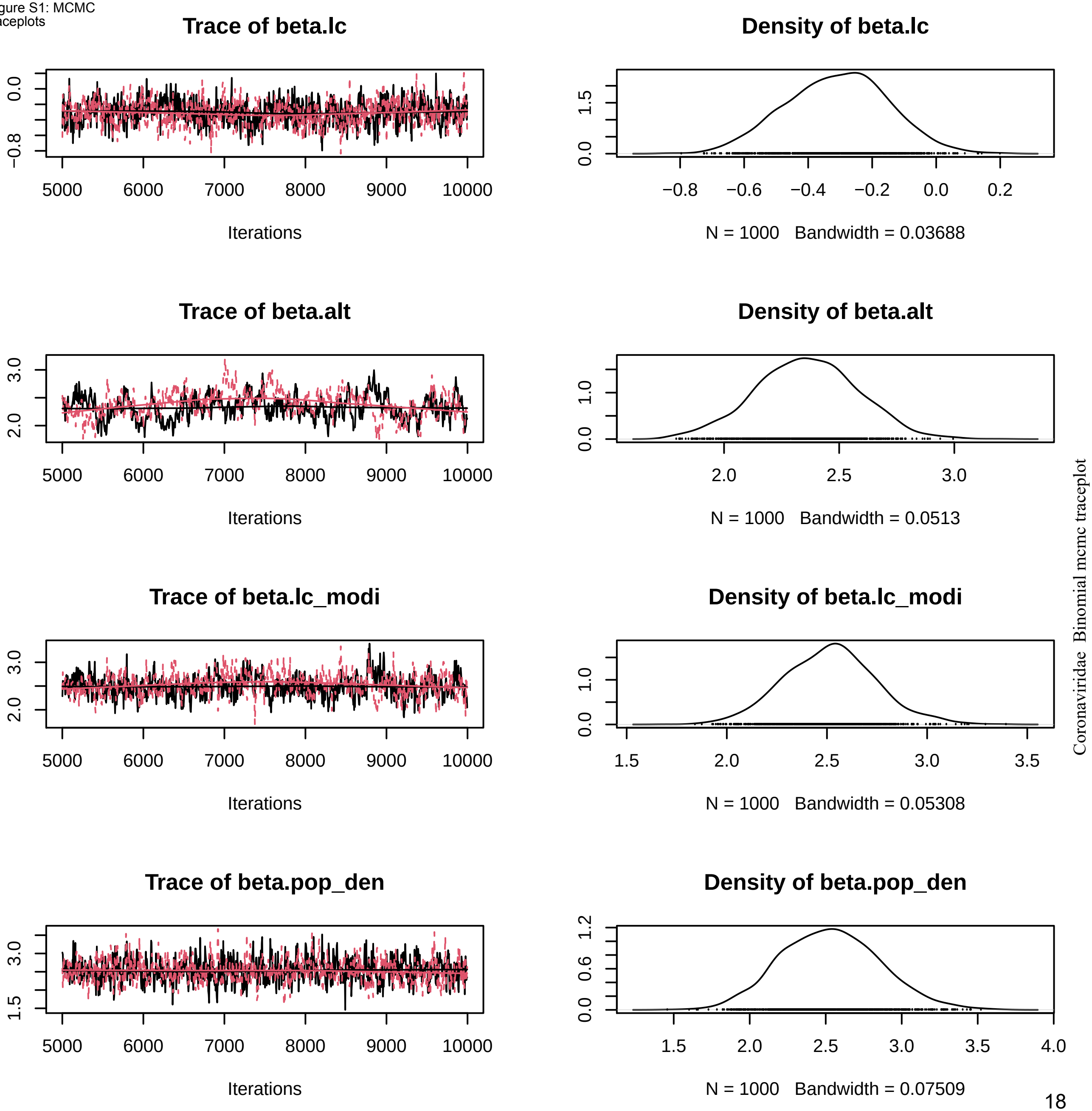

Figure S1: MCMC traceplots

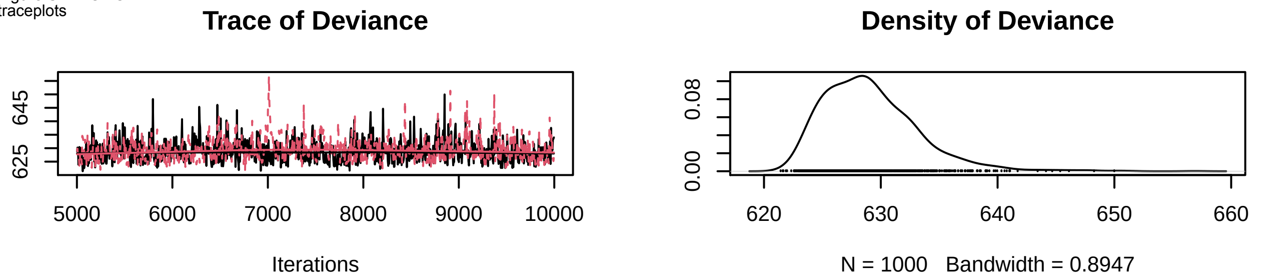

Coronaviridae Binomial mcmc traceplot

Figure S1: MCMC traceplots

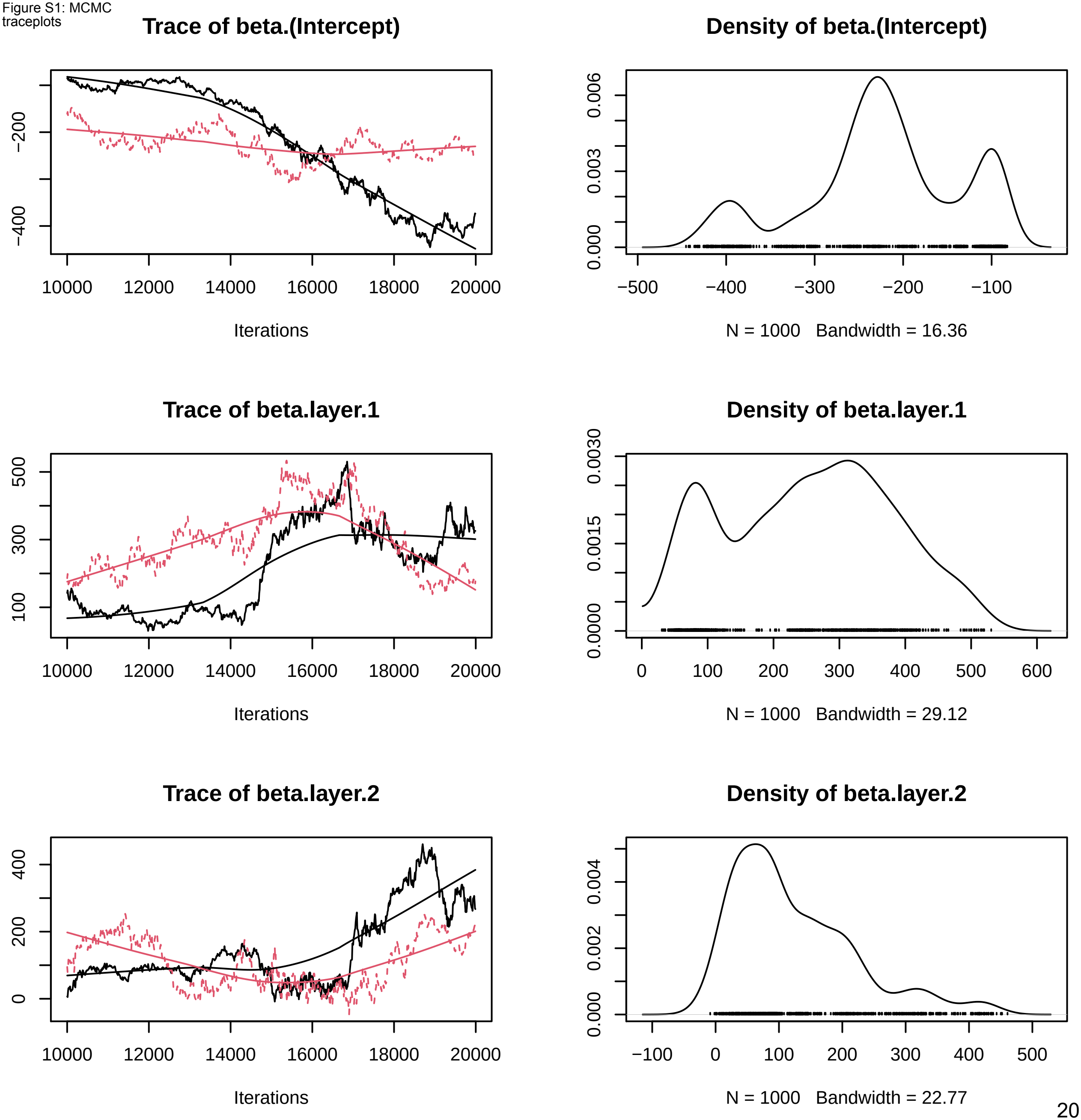

Coronaviridae ZIB mcmc traceplot

Figure S1: MCMC traceplots

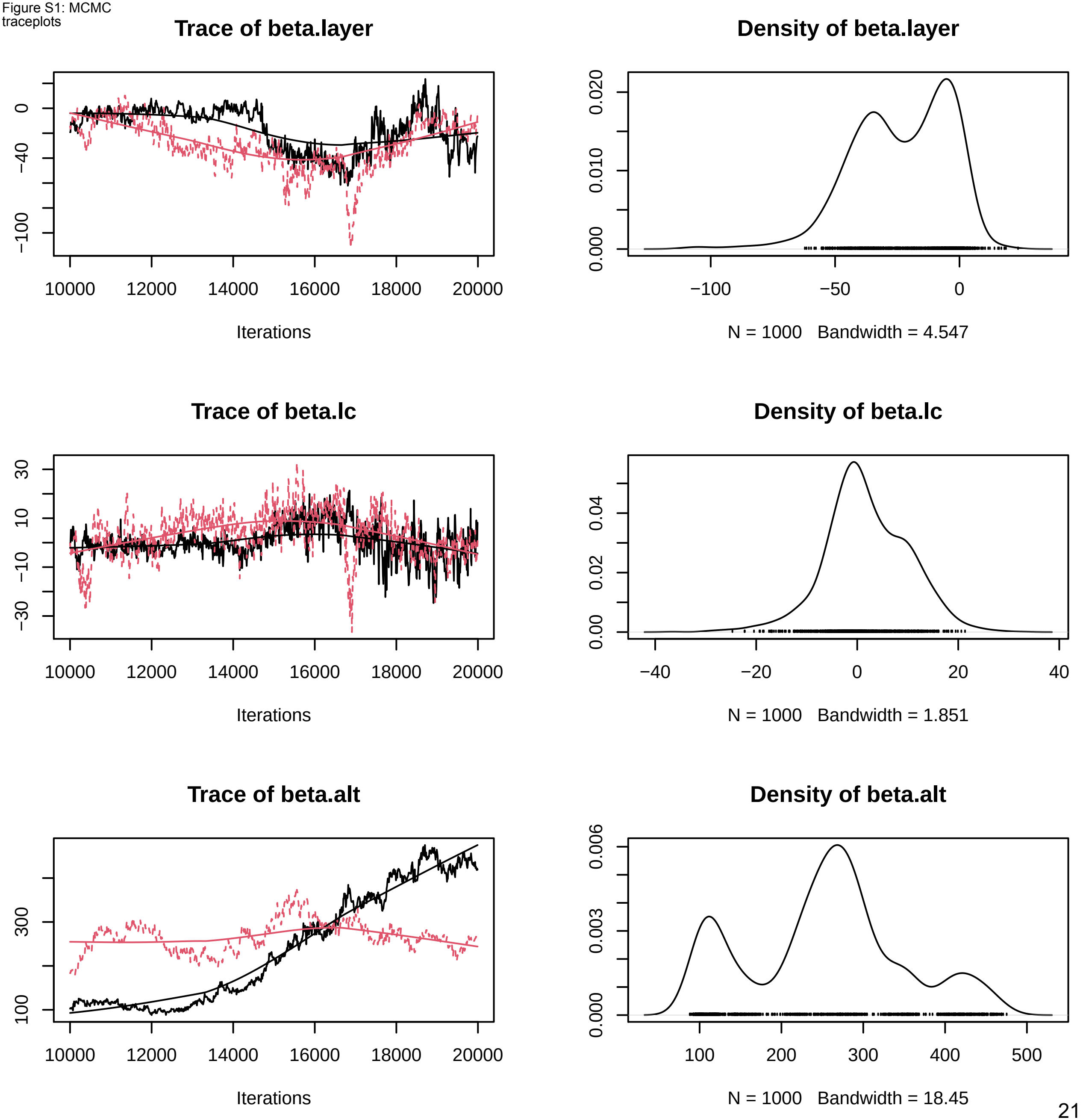

Coronaviridae ZIB mcmc traceplot

Figure S1: MCMC traceplots

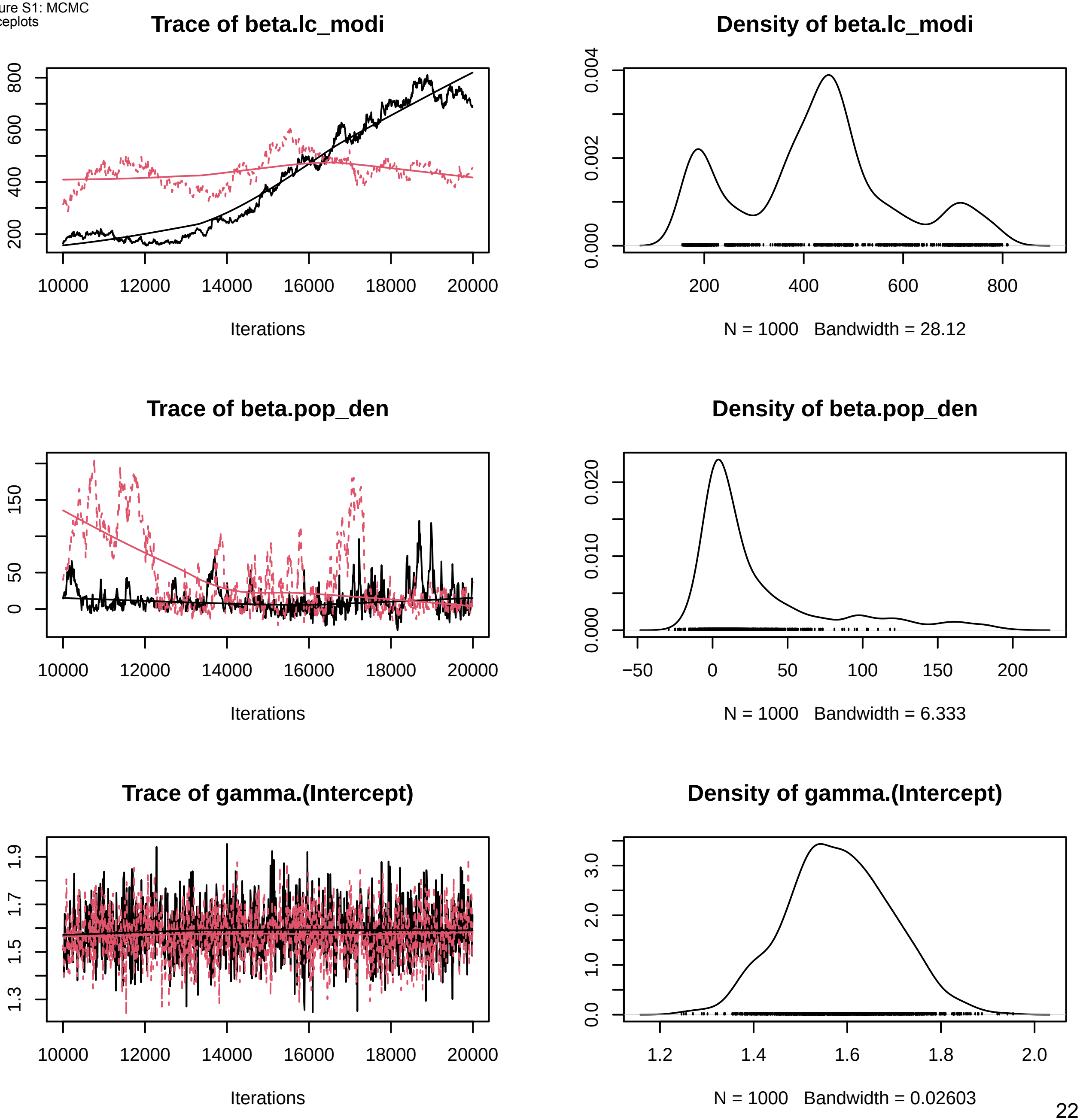

Coronaviridae ZIB mcmc traceplot

Figure S1: MCMC traceplots

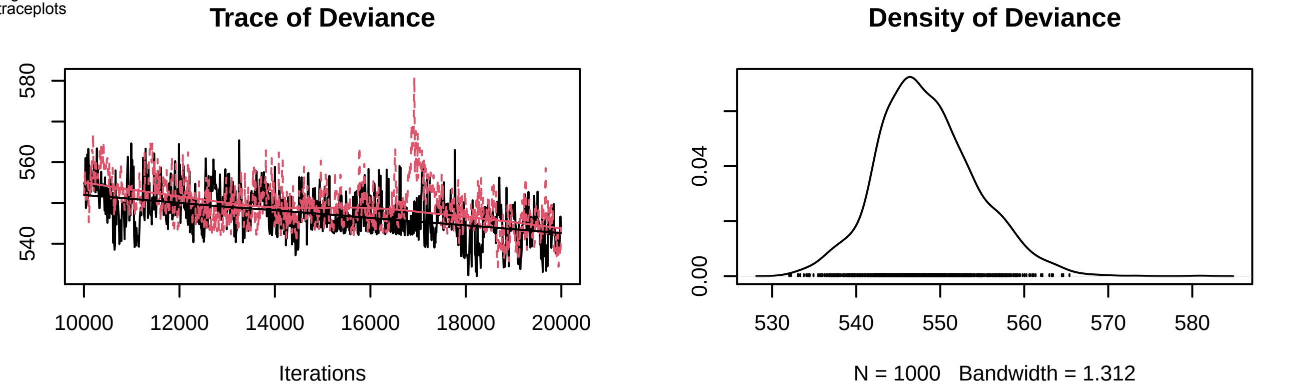

Coronaviridae ZIB mcmc traceplot

Figure S1: MCMC traceplots

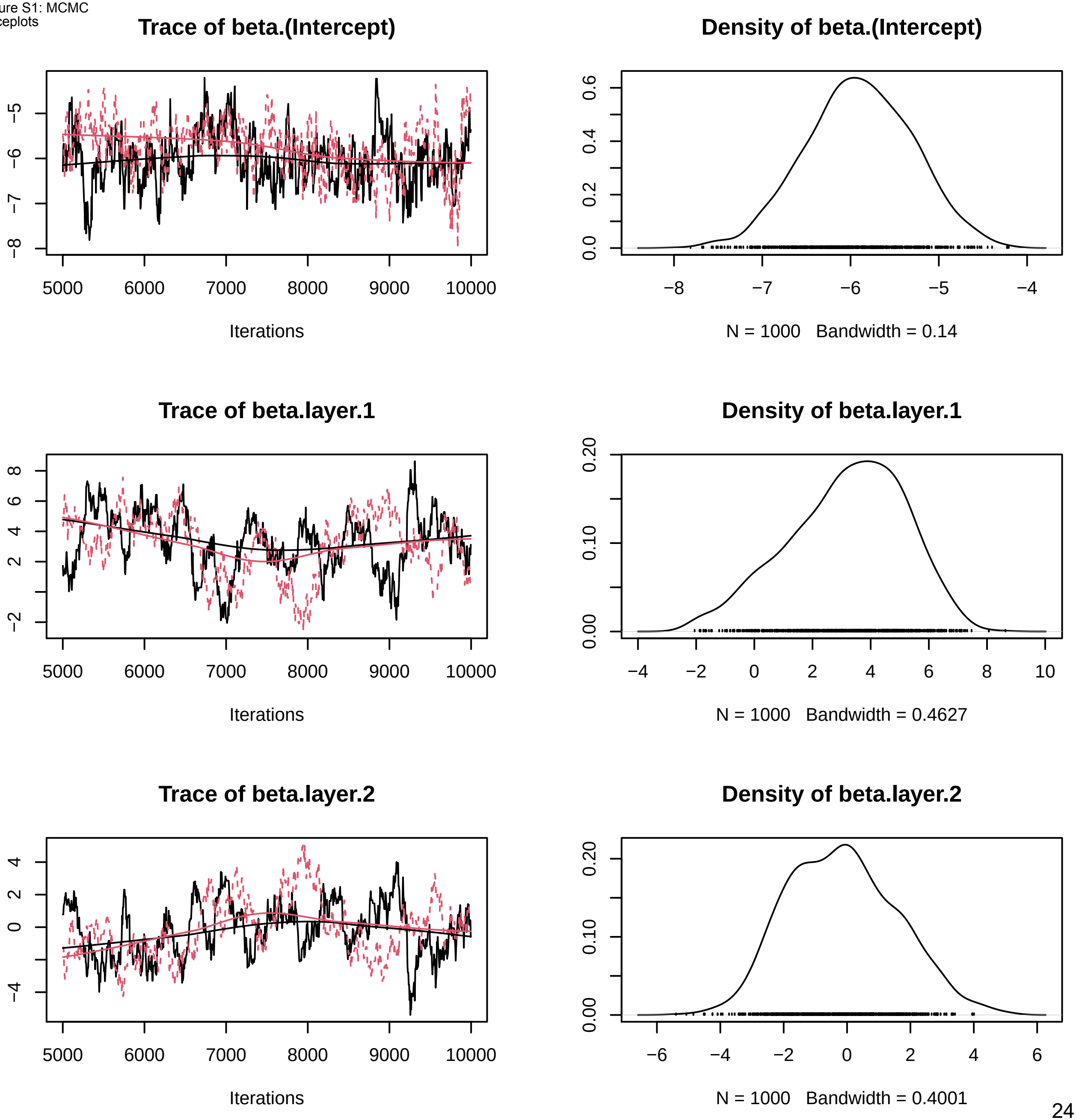

Coronaviridae Binomial iCAR mcmc traceplot

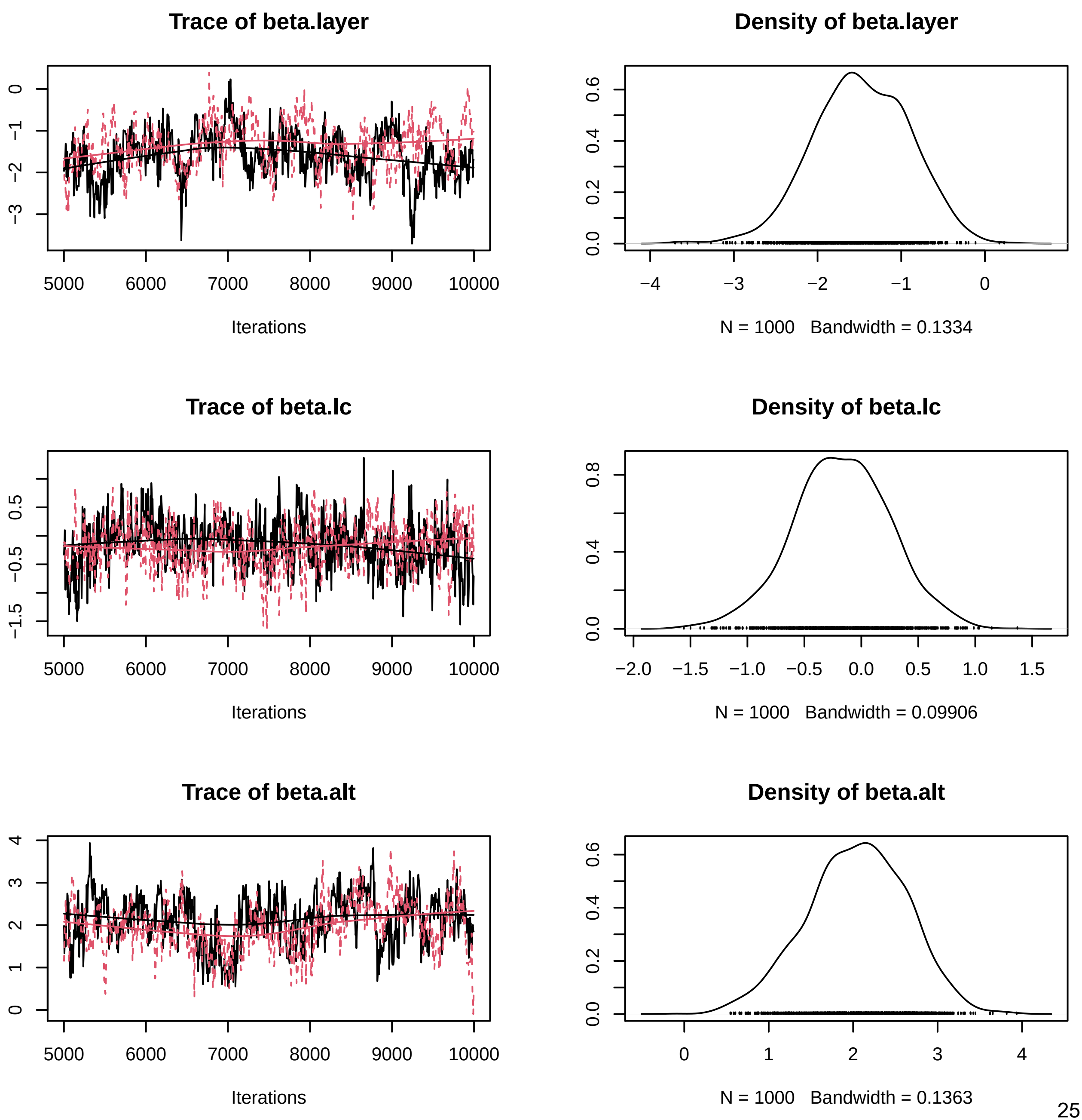

Coronaviridae Binomial iCAR mcmc traceplot

Figure S1: MCMC traceplots

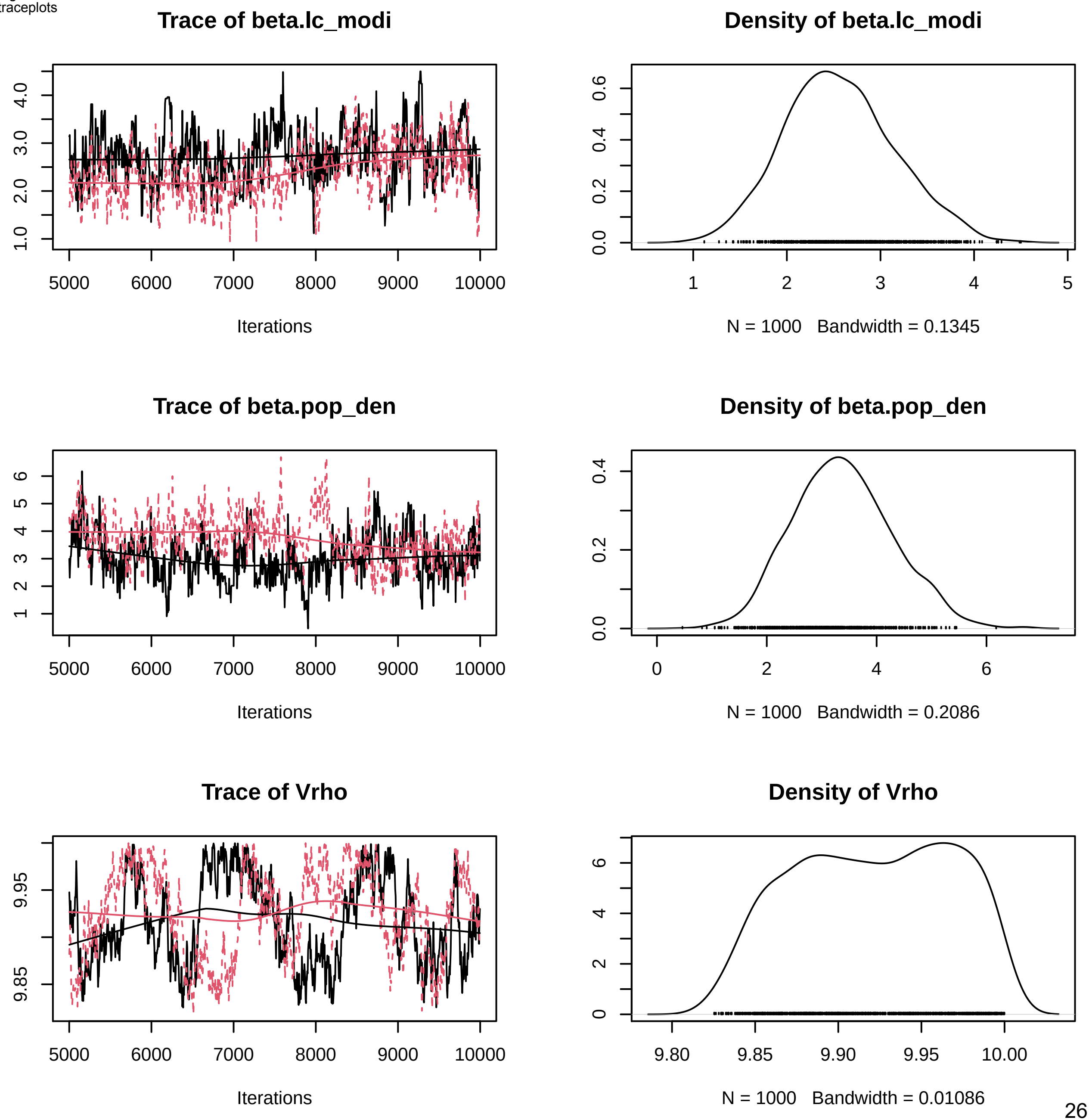

Coronaviridae Binomial iCAR mcmc traceplot

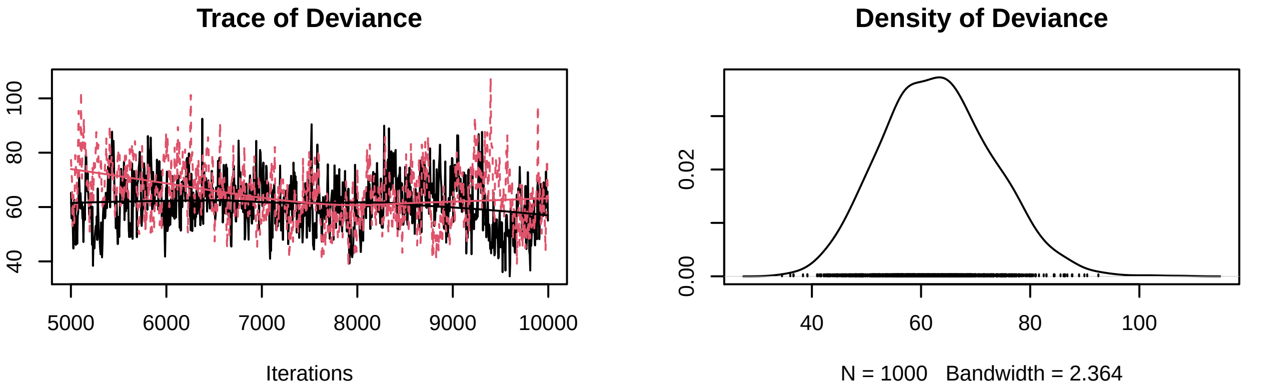

Coronaviridae Binomial iCAR mcmc traceplot

Figure S1: MCMC traceplots

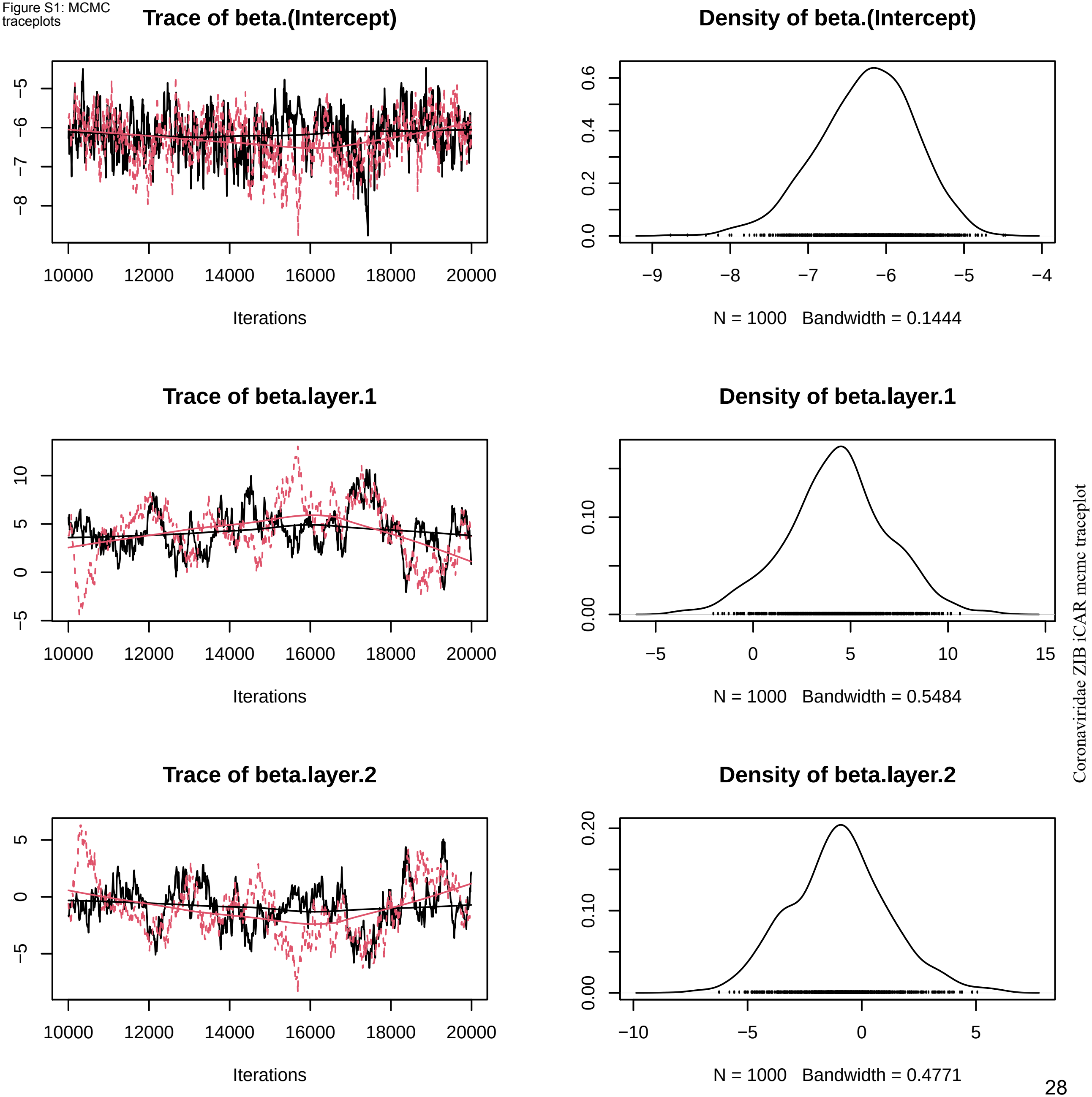

Figure S1: MCMC traceplots

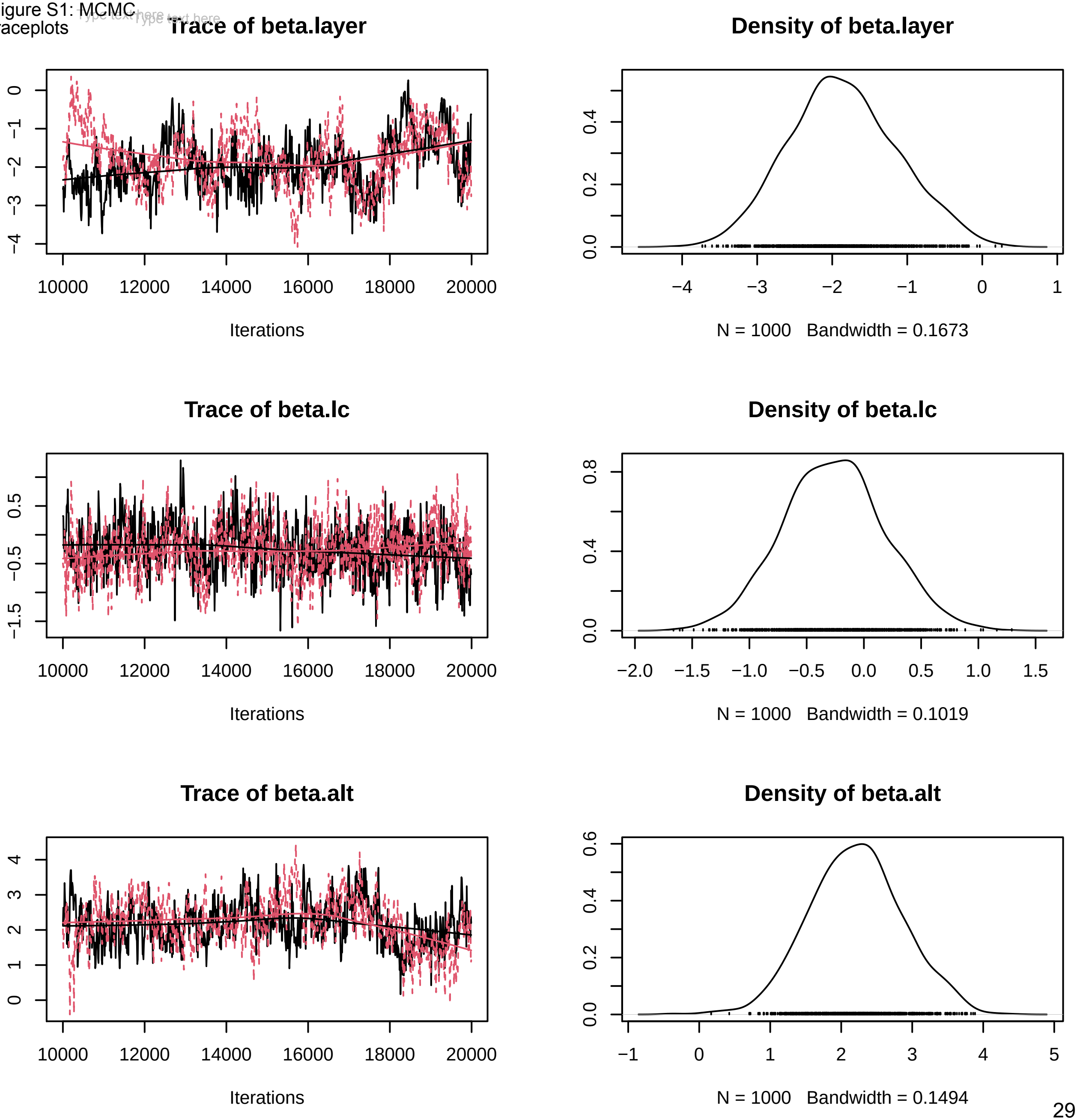

Coronaviridae ZIB iCAR mcmc traceplot

Figure S1: MCMC traceplots

Type text here

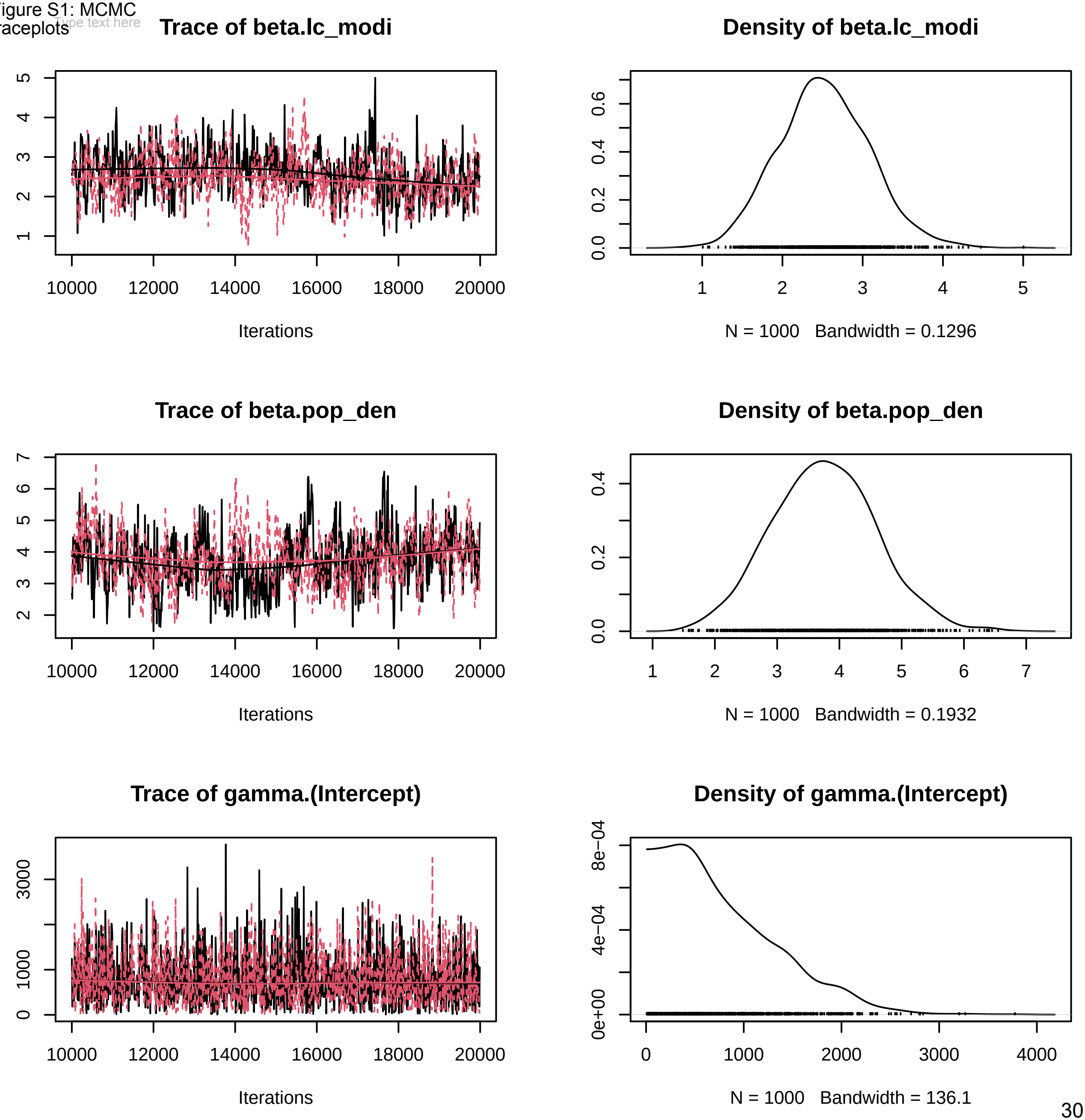

Coronaviridae ZIB iCAR mcmc traceplot

Figure S1: MCMC traceplots

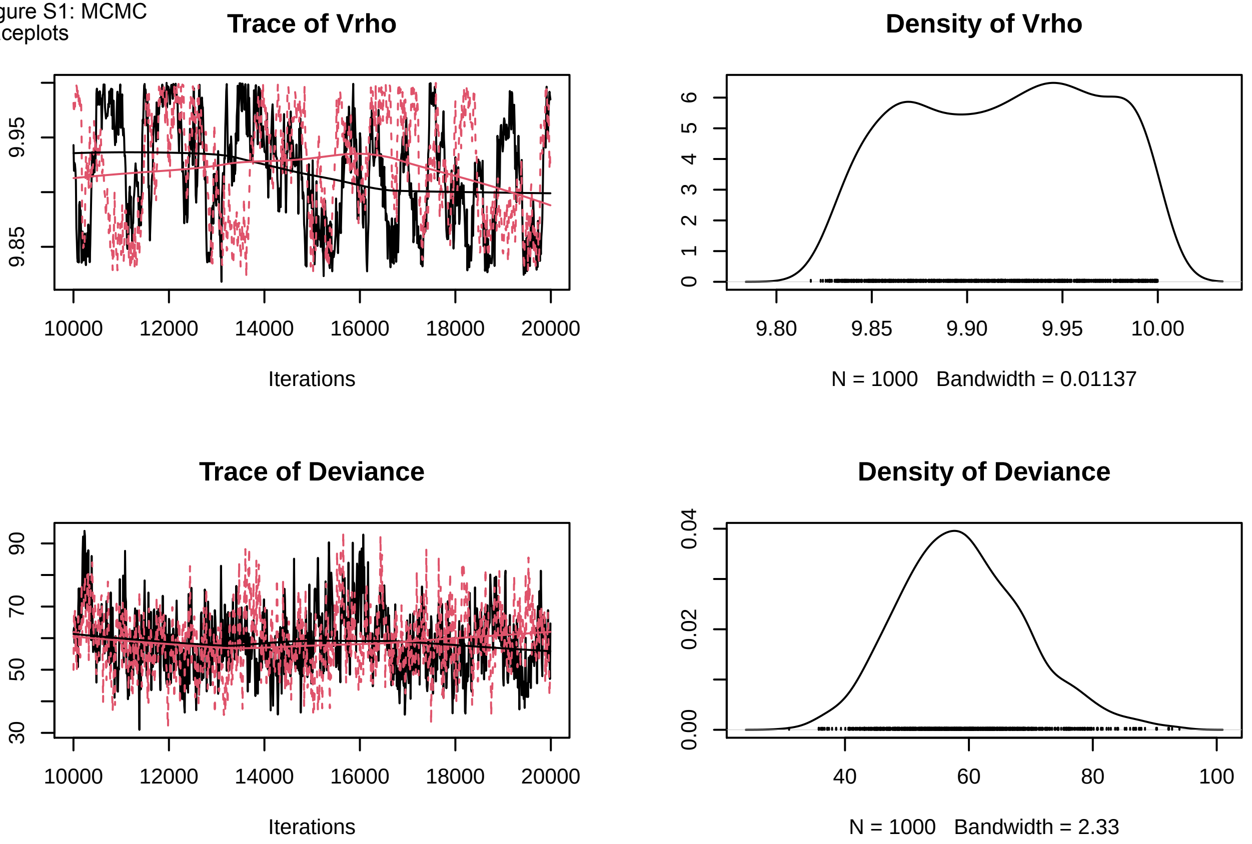

Coronaviridae ZIB iCAR mcmc traceplot

Figure S1: MCMC traceplots

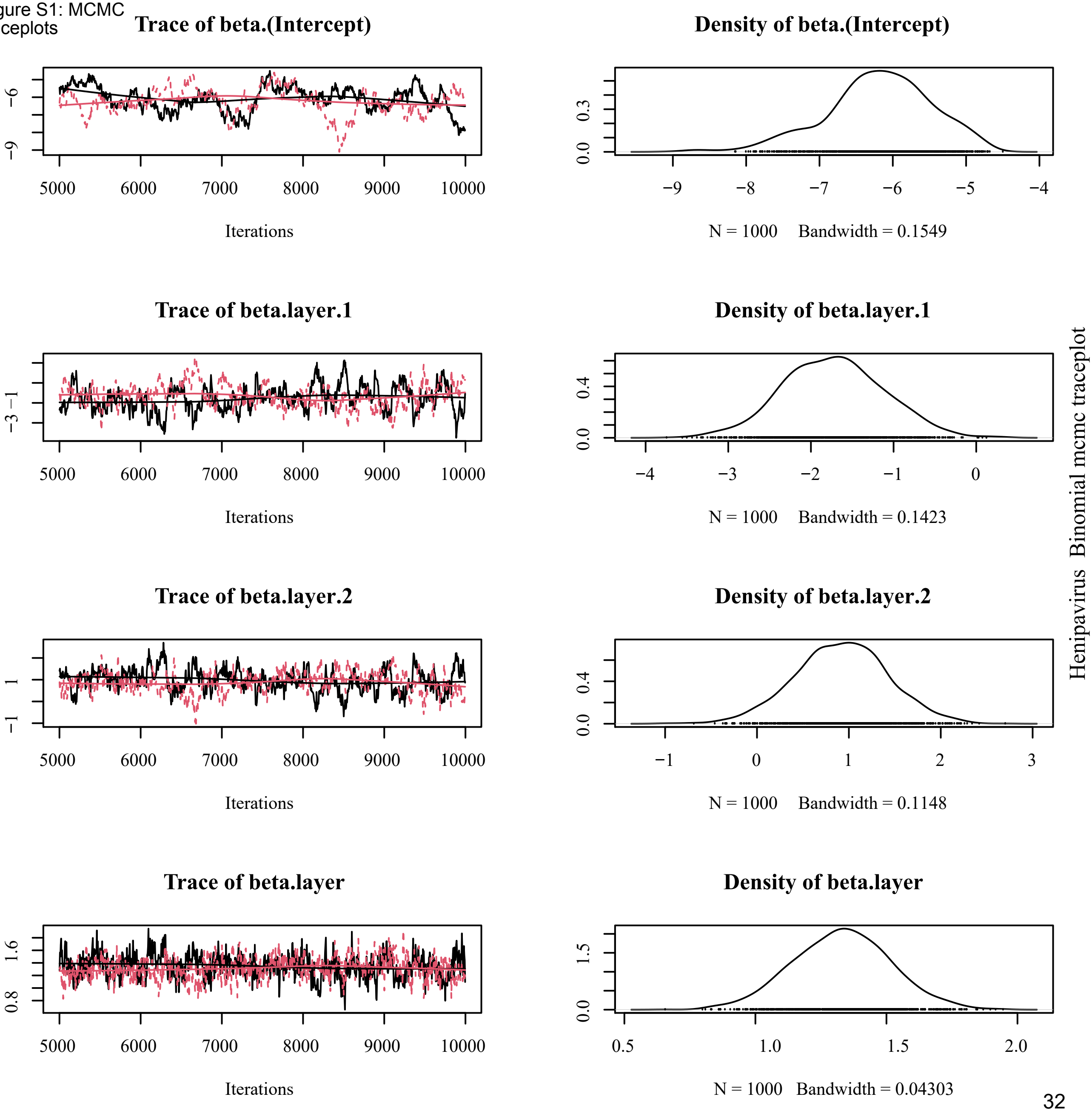

Figure S1: MCMC traceplots

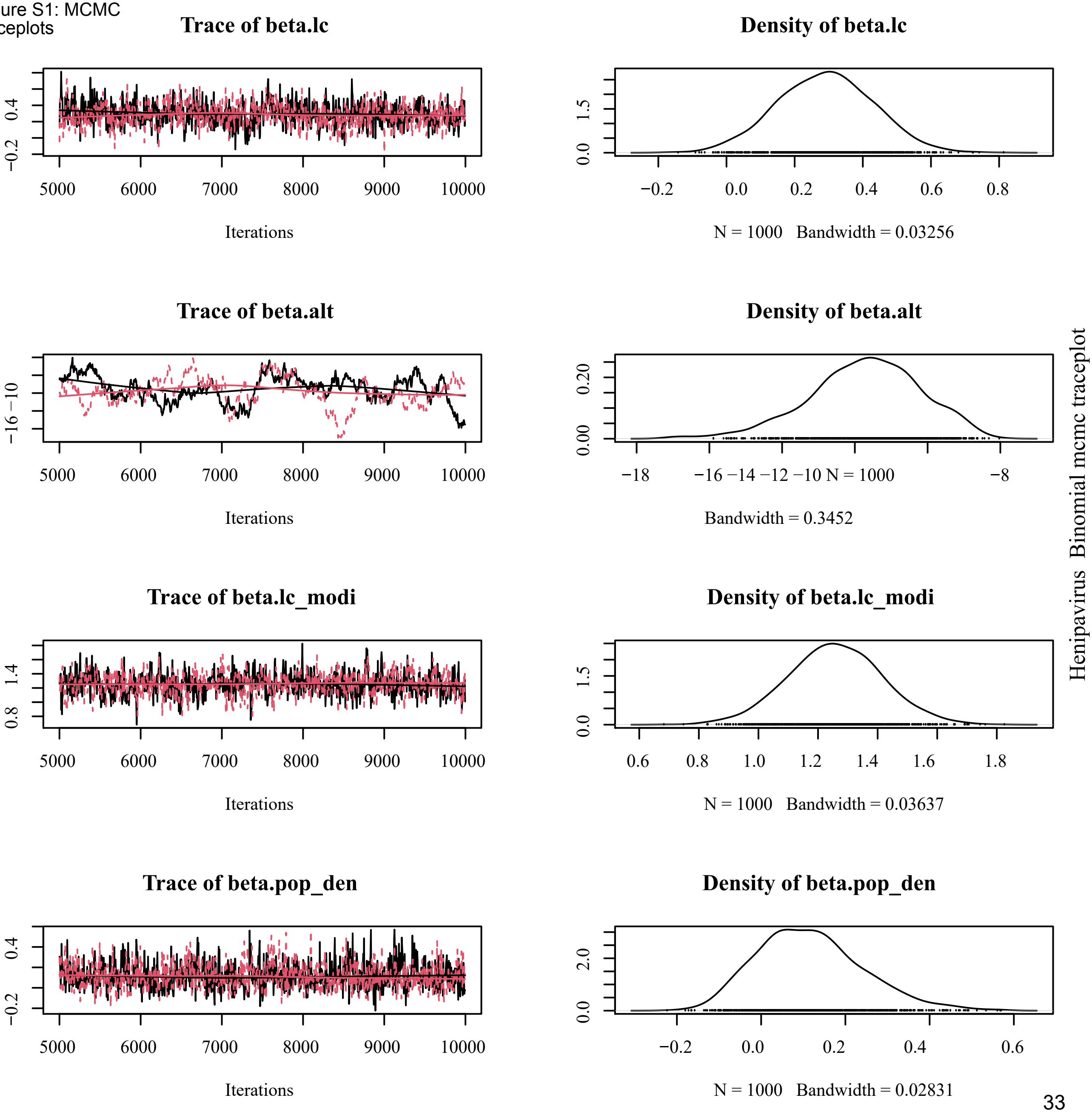

Figure S1: MCMC traceplots

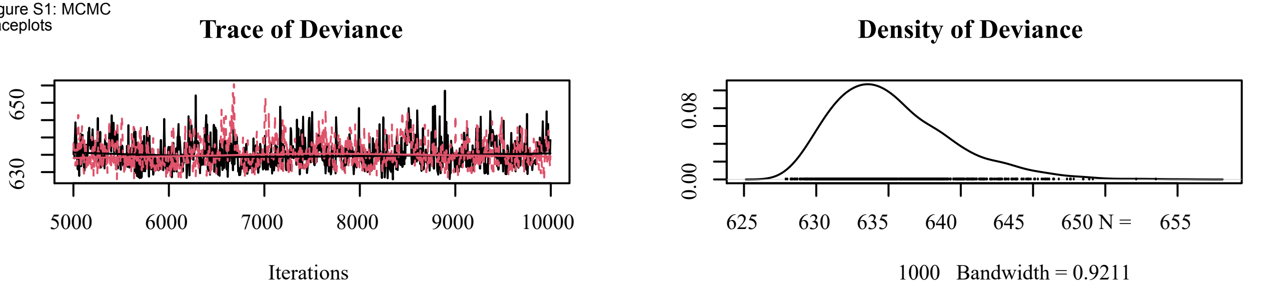

Henipavirus Binomial mcmc traceplot

Figure S1: MCMC traceplots

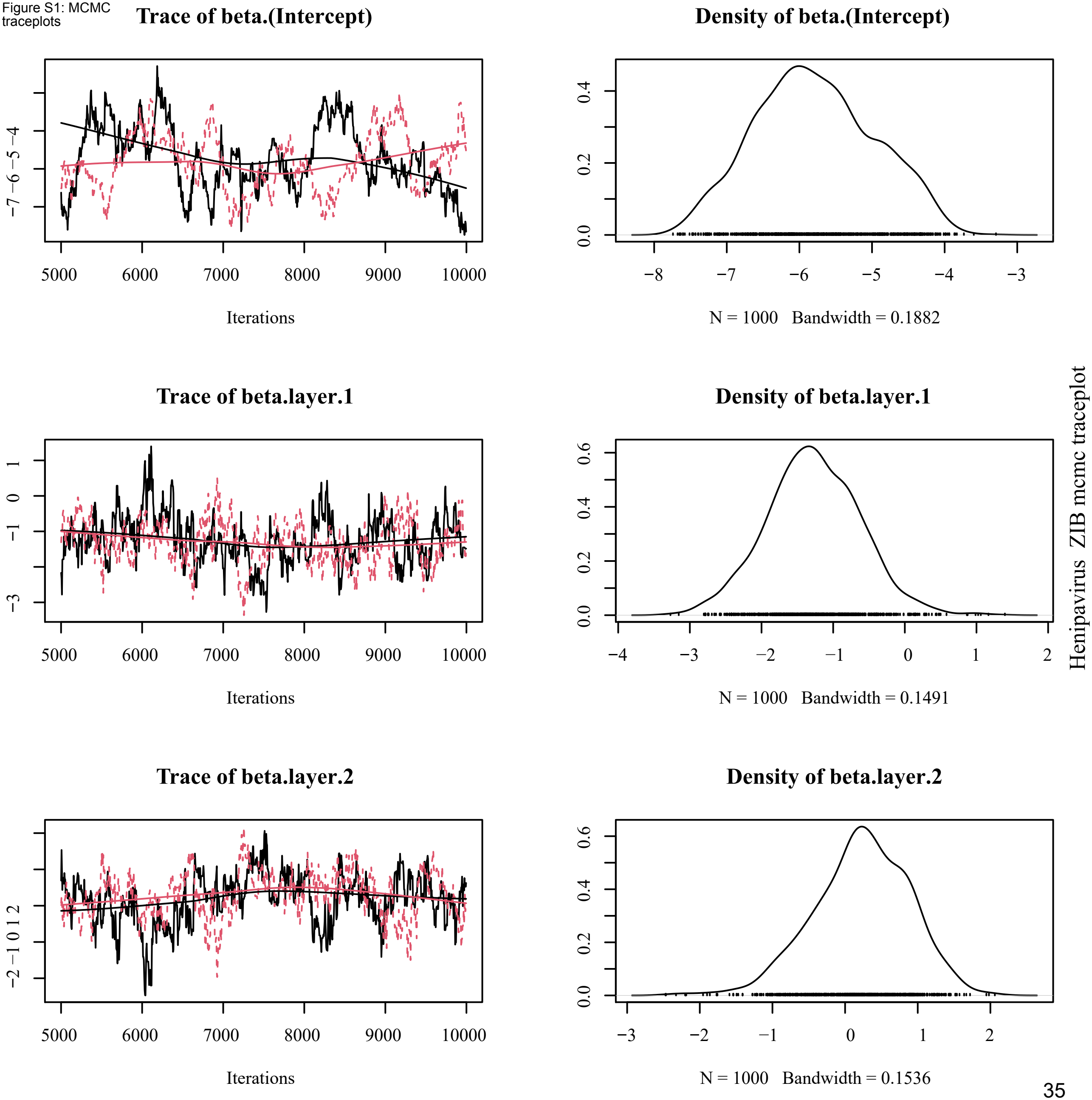

Figure S1: MCMC traceplots

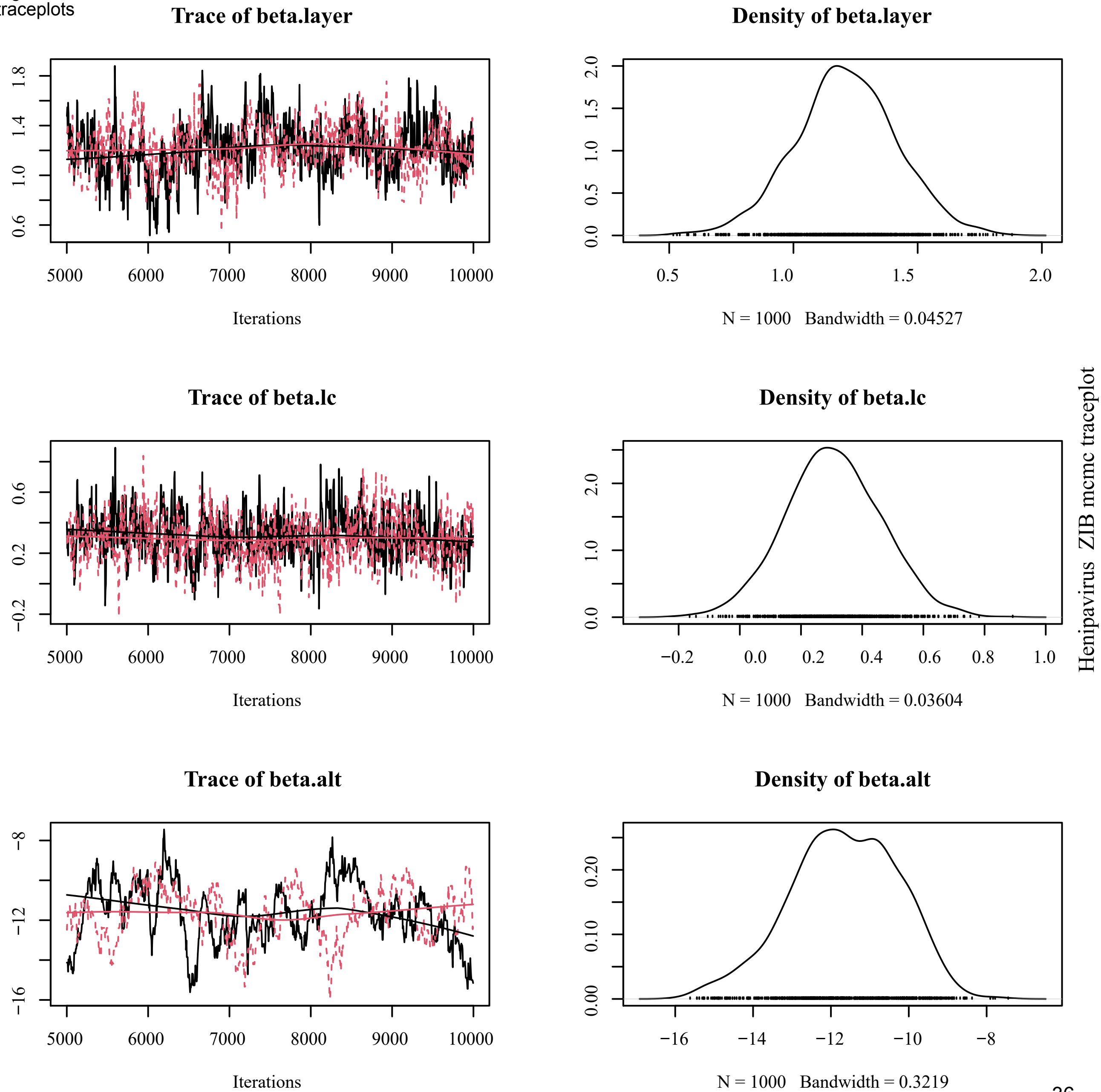

Figure S1: MCMC traceplots

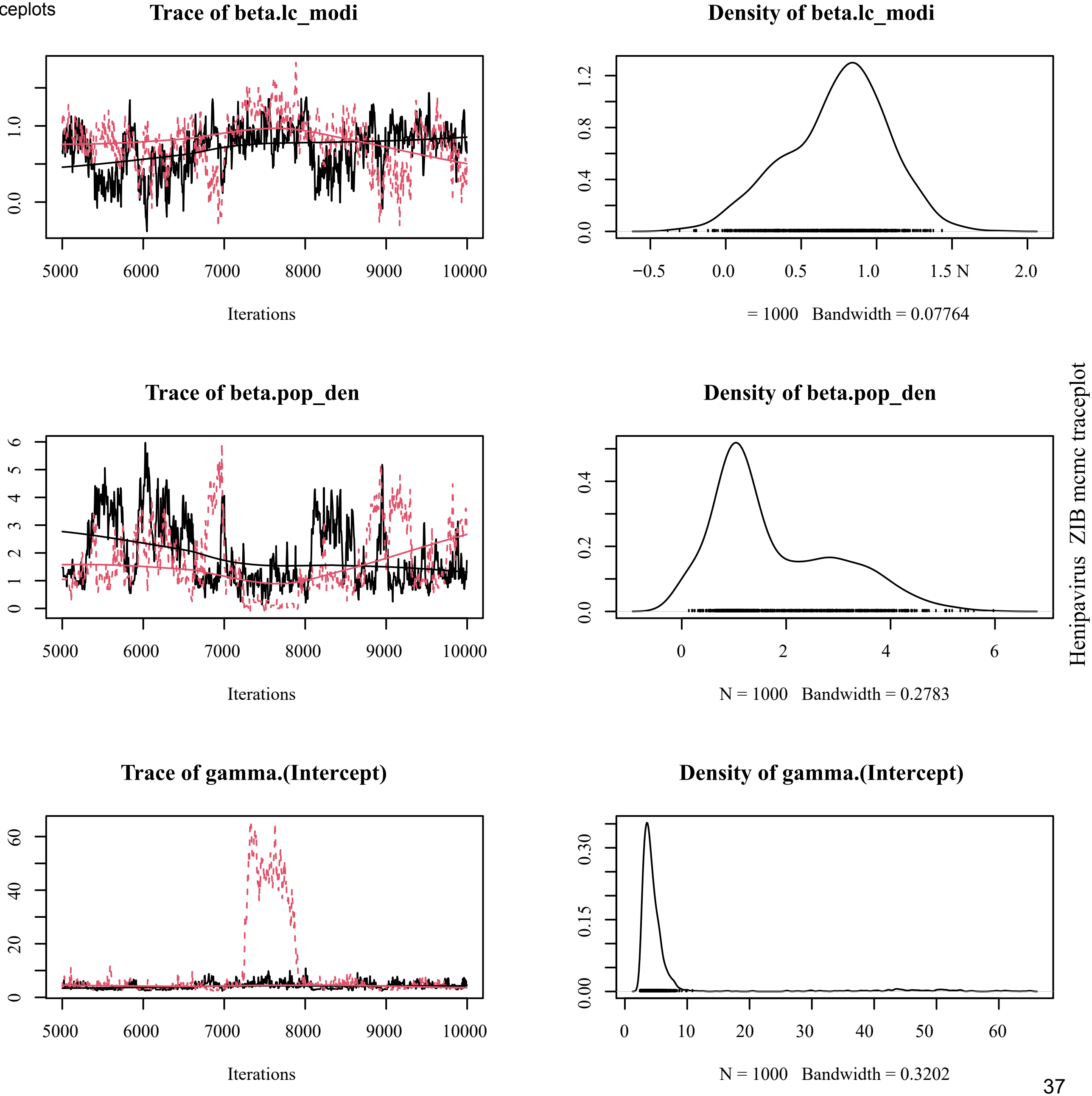

Figure S1: MCMC traceplots

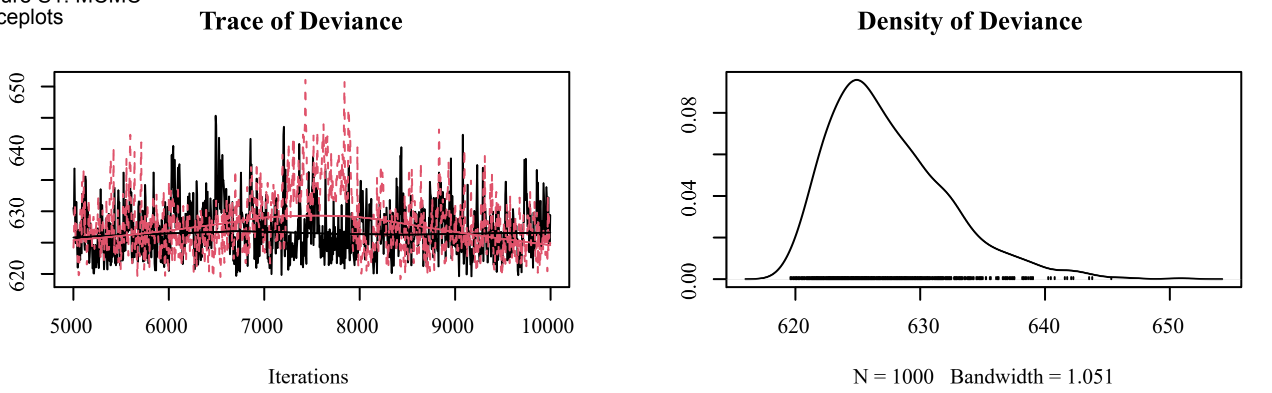

Henipavirus ZIB mcmc traceplot

Figure S1: MCMC traceplots

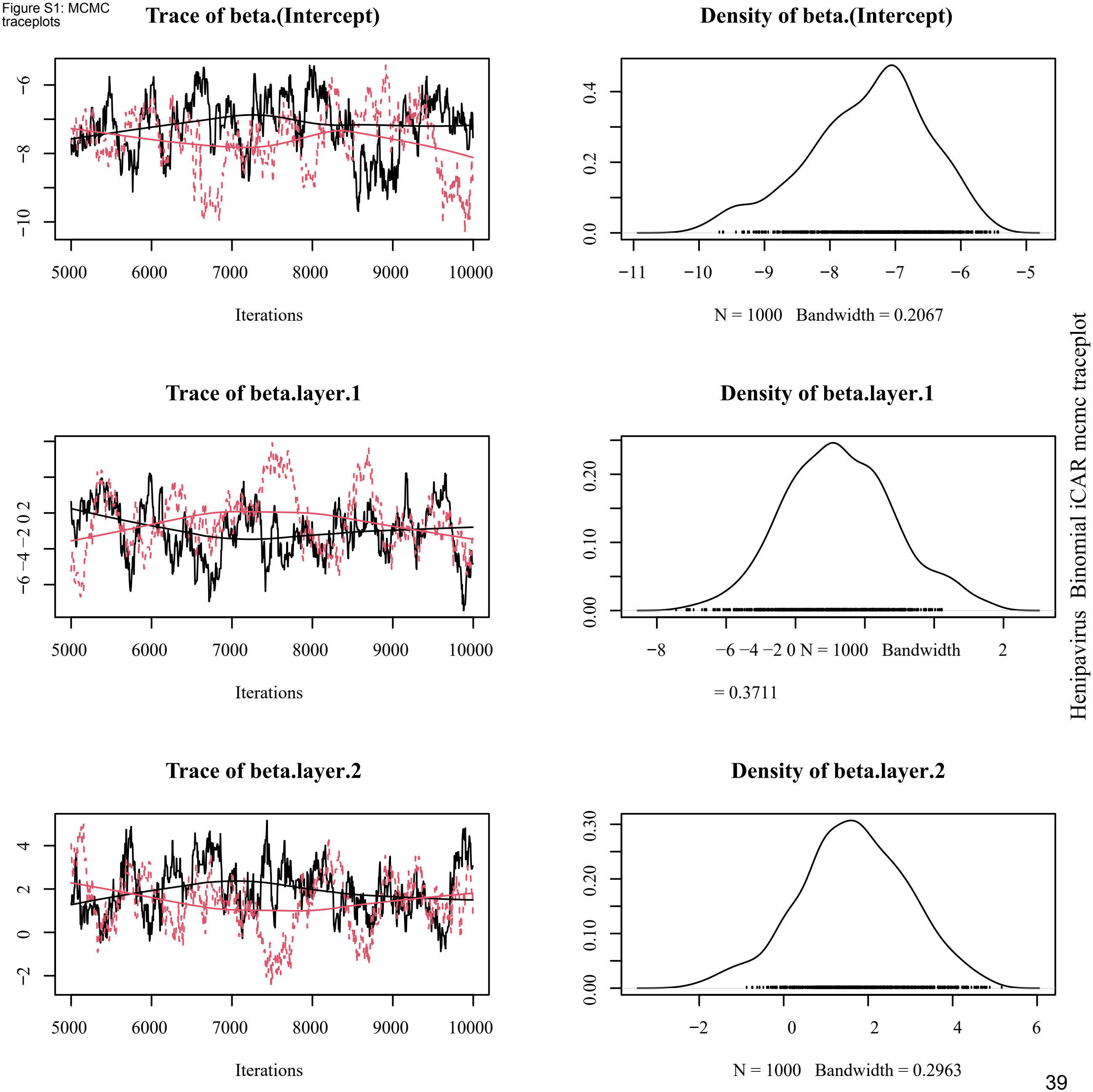

Figure S1: MCMC traceplots

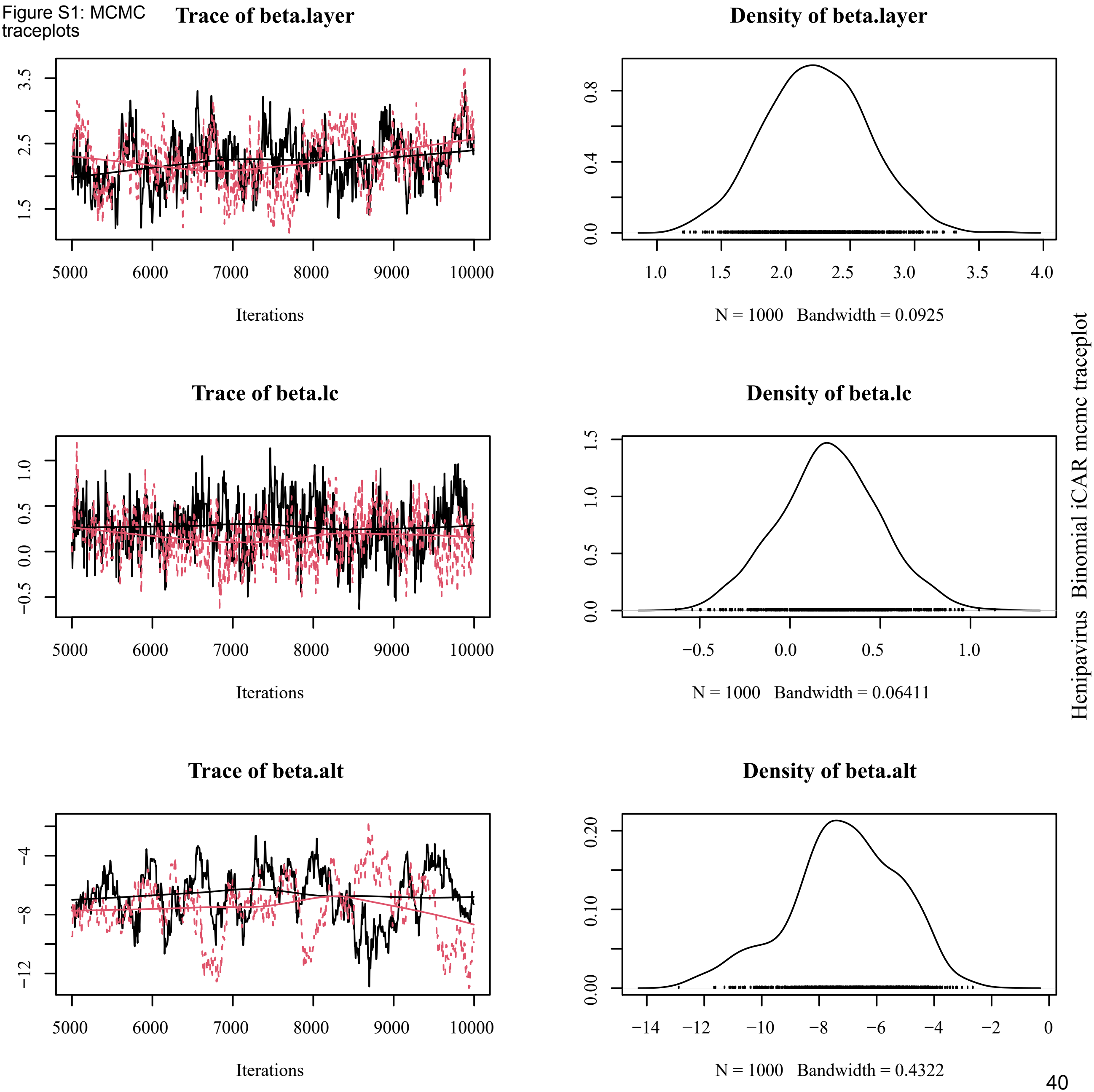

Figure S1: MCMC traceplots

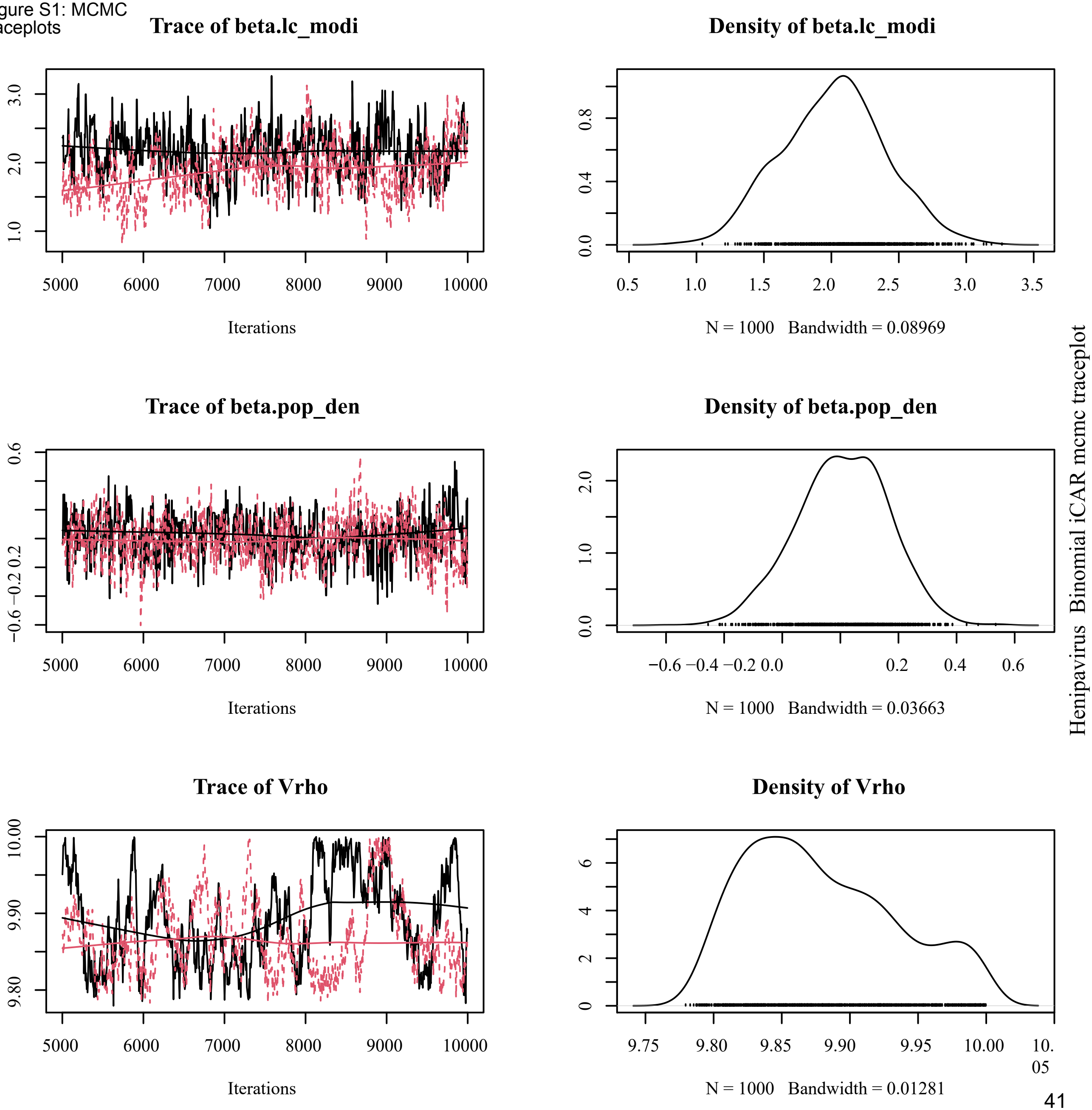

Figure S1: MCMC traceplots

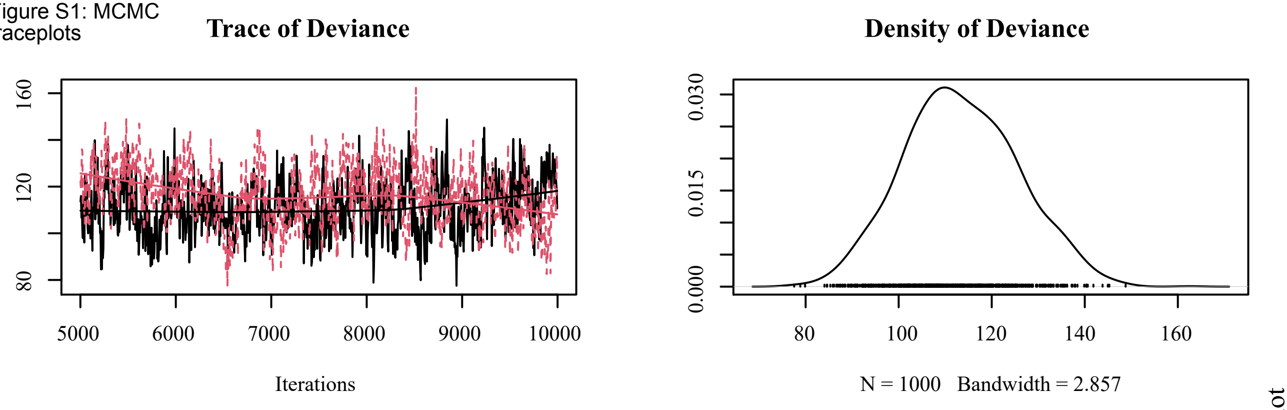

Henipavirus Binomial iCAR mcmc traceplot

Figure S1: MCMC traceplots

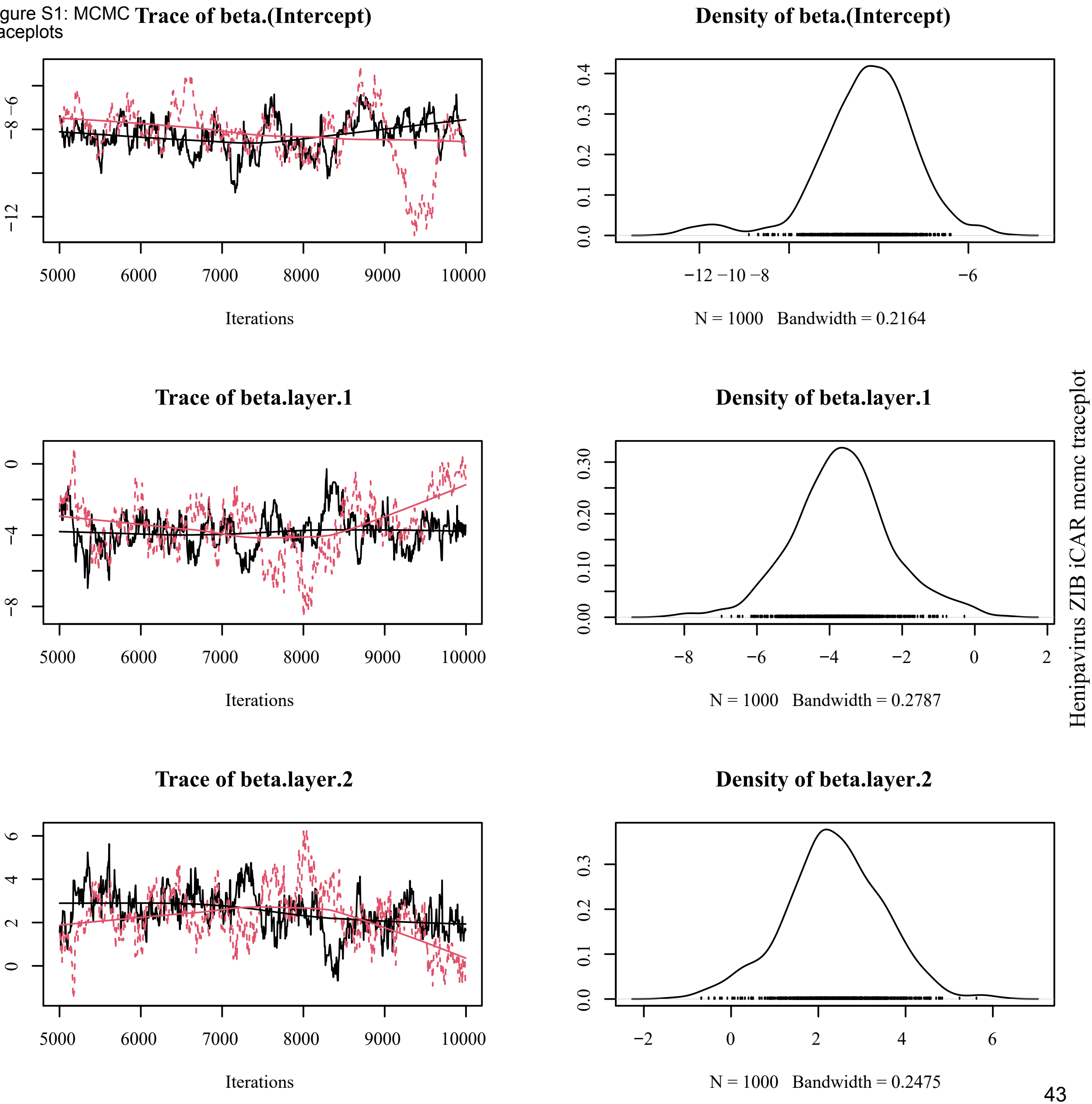

Figure S1: MCMC traceplots

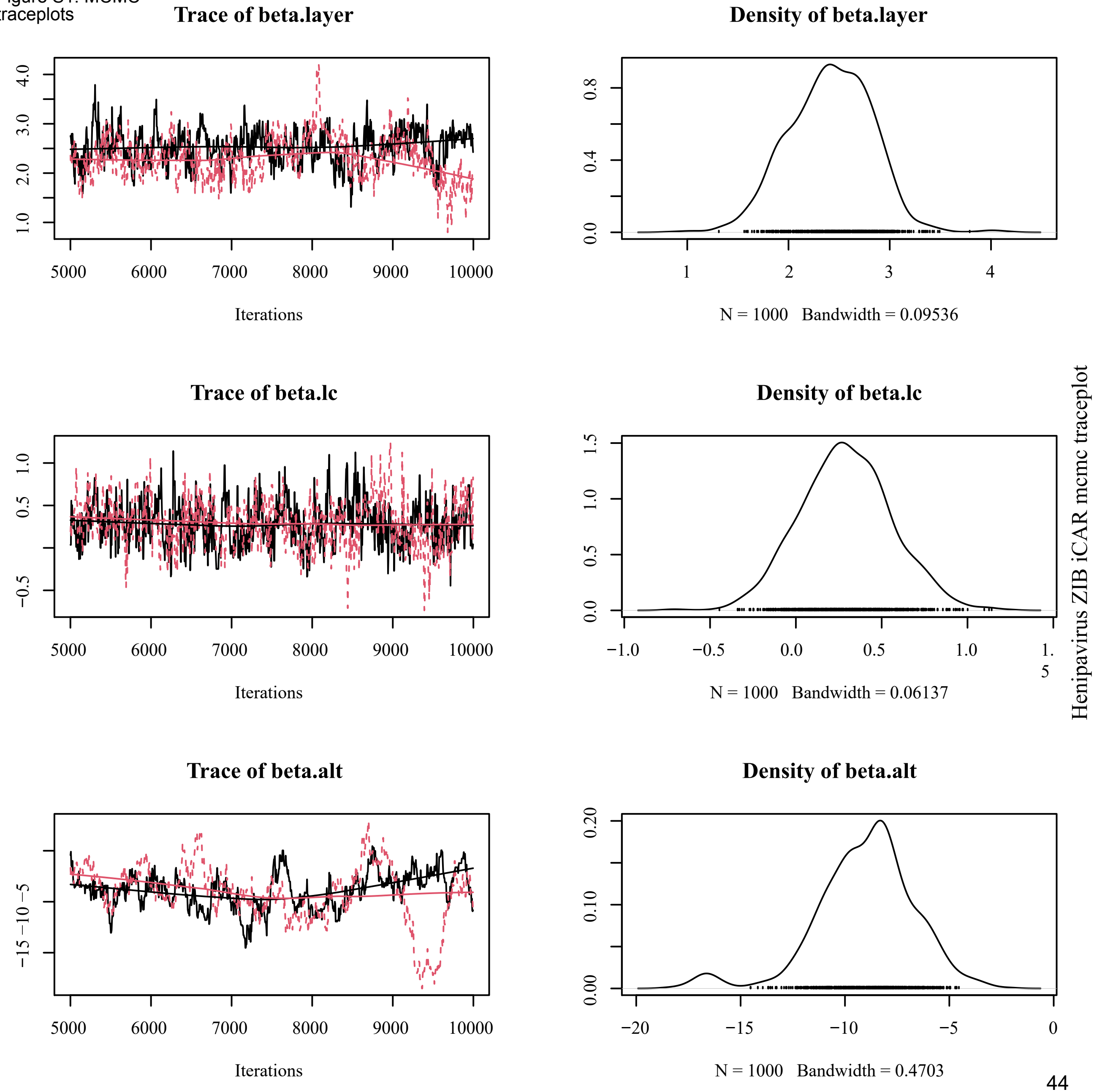

Figure S1: MCMC traceplots

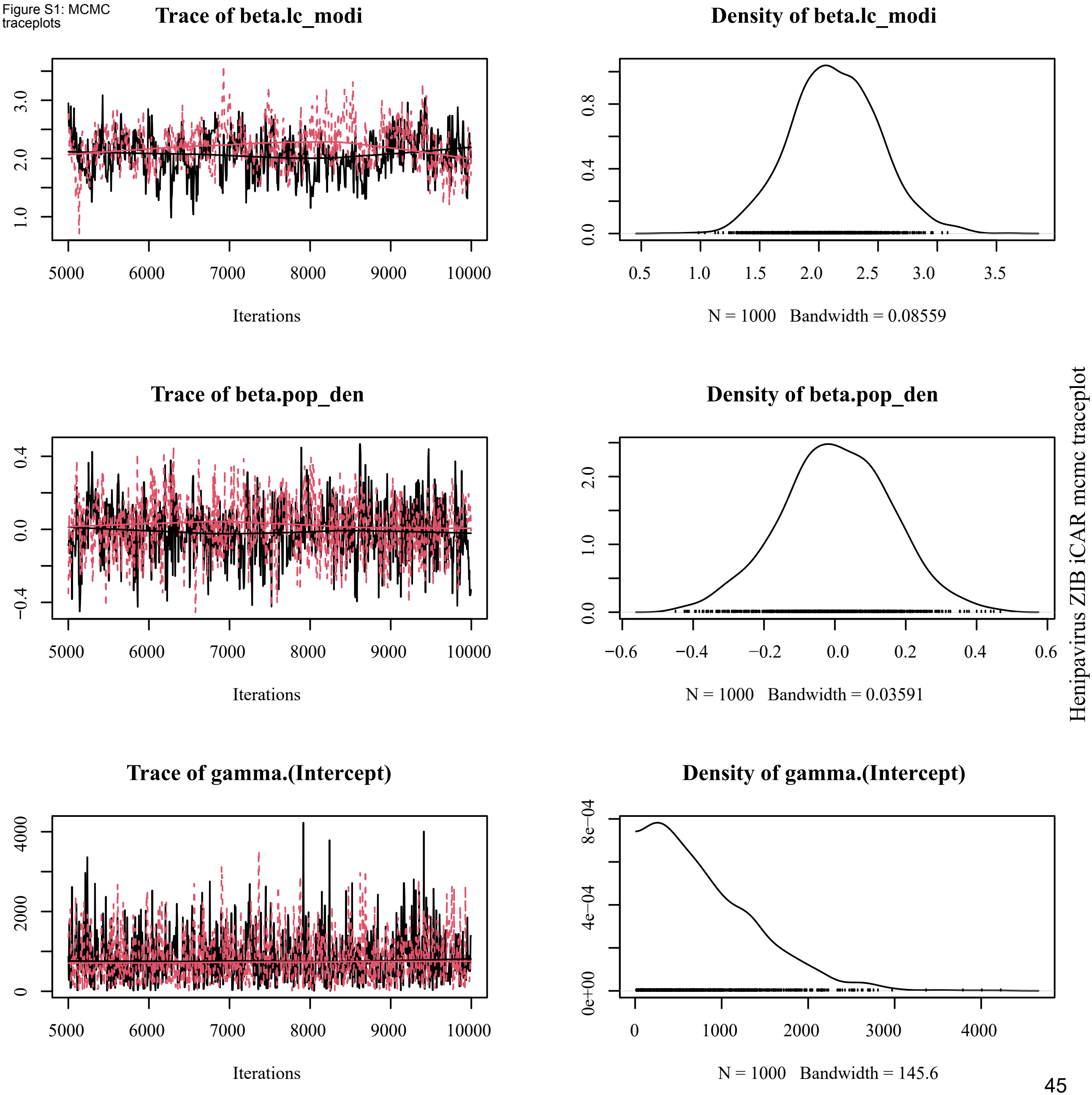

Figure S1: MCMC traceplots

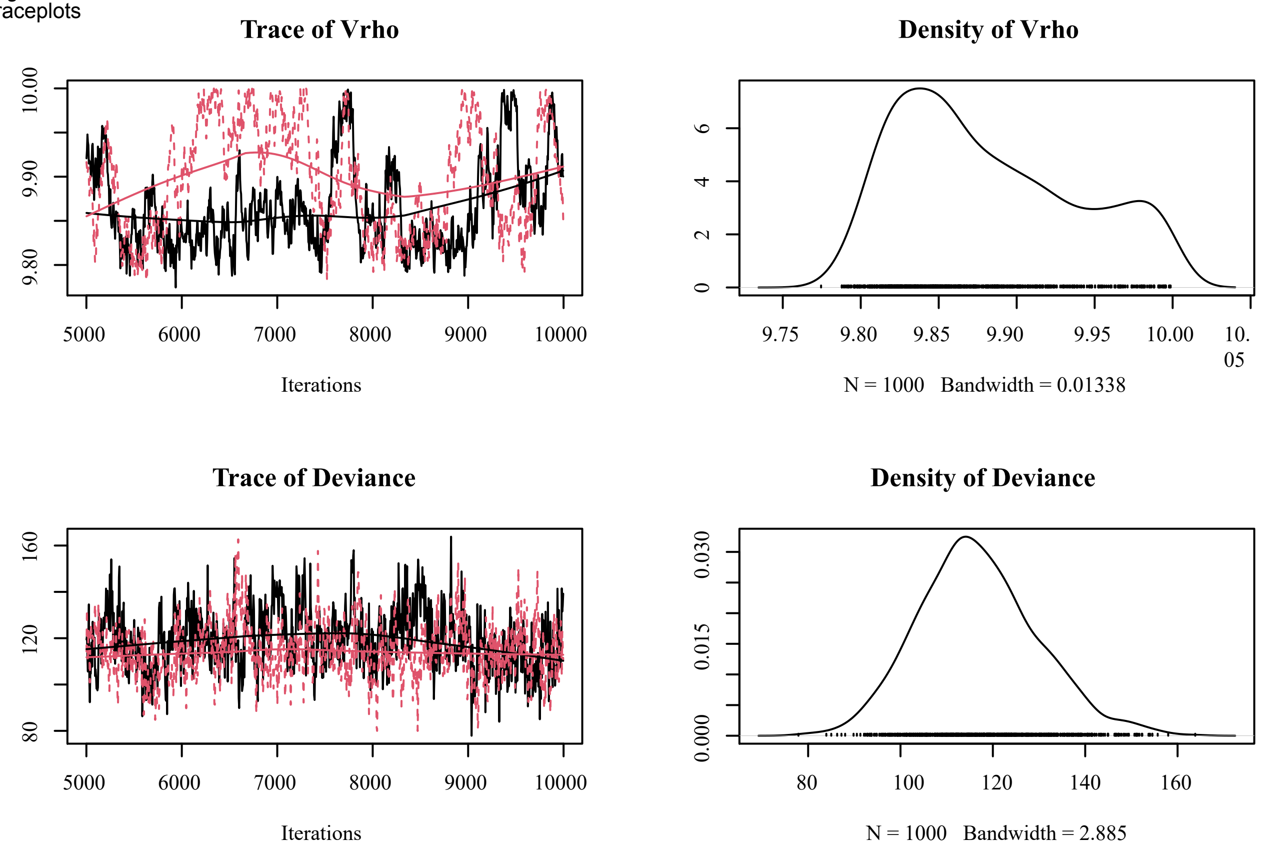

Henipavirus ZIB iCAR mcmc traceplot

Filoviridae- Binomial model

Iterations = 5001:9996 Thinning
interval = 5 Number of chains = 2
Sample size per chain = 1000

1. Empirical mean and standard deviation for each variable,
plus standard error of the mean:

Mean SD Naive SE Time-series SE
beta.(Intercept) -5.0585 0.38275 0.008558 0.035358
beta.layer.1 2.8126 0.63396 0.014176 0.091549
beta.layer.2 -1.2756 0.43896 0.009815 0.044785
beta.layer 1.5379 0.28266 0.006321 0.020456
beta.lc -0.4537 0.23741 0.005309 0.016624 1.5273
beta.alt 0.42220 0.009441 0.043218
beta. lc_ modi 1.2101 0.24662 0.005515 0.019175
beta.pop_den 1.4288 0.52485 0.011736 0.028831

Vrho 9.9117 0.06608 0.001478 0.009067 Deviance
268.5234 18.59034 0.415693 1.368309

2. Quantiles for each variable:

2.5% 25% 50% 75% 97.5%
beta.(Intercept) -5.9281 -5.2883 -5.0267 -4.8015 -4.377118
beta.layer.1 1.6322 2.3601 2.8213 3.2370 4.093664
beta.layer.2 -2.1452 -1.5521 -1.2730 -0.9835 -0.416579
beta.layer 0.9906 1.3403 1.5413 1.7226 2.083984 beta.lc
-0.9343 -0.6152 -0.4509 -0.2934 -0.008502 beta.alt 0.7510
1.2272 1.5100 1.8304 2.359139 beta.lc_modi 0.7452 1.0401
1.2039 1.3657 1.720882 beta.pop_den 0.4723 1.0645 1.3920
1.7538 2.541129

Vrho 9.7524 9.8724 9.9292 9.9623 9.995419
Deviance 234.5247 255.2666 268.5496 280.7599 307.406395

Potential scale reduction factors:

Point est. Upper C.I.

beta.(Intercept) 1.08 1.11
beta.layer.1 1.05 1.13
beta.layer.2 1.10 1.18
beta.layer 1.04 1.16
beta.lc
beta.alt 1.01 1.03
beta.lc_modi 1.04 1.17
beta.pop_den 1.02 1.10
Vrho Deviance 1.03 1.12
1.03 1.15
1.02 1.08

Multivariate psrf **1.01**

Filoviridae- ZIB model

Iterations = 5001:9996 Thinning
interval = 5 Number of chains = 2
Sample size per chain = 1000

1. Empirical mean and standard deviation for each variable,
plus standard error of the mean:

Mean SD Naive SE Time-series SE
beta.(Intercept) -2.4360 0.2005 0.004483 0.014305
beta.layer.1 3.0160 0.3328 0.007443 0.027527
beta.layer.2 -1.4385 0.2119 0.004739 0.010294
beta.layer 1.4636 0.1627 0.003638 0.007255
beta.lc -0.5116 0.1397 0.003124 0.005707 1.8564
beta.alt 0.2289 0.005119 0.016787
beta. lc_ modi 0.9691 0.1217 0.002721 0.004821
beta. pop_ den 1.4301 0.3510 0.007849 0.011363
Deviance 839.2431 3.9822 0.089046 0.202869

2. Quantiles for each variable:

2.5% 25% 50% 75% 97.5%
beta.(Intercept) -2.8509 -2.5694 -2.4249 -2.2968 -2.0594
beta.layer.1 2.4301 2.7740 3.0153 3.2328 3.6853
beta.layer.2 -1.8613 -1.5798 -1.4316 -1.2940 -1.0319
beta.layer 1.1559 1.3522 1.4624 1.5733 1.7776
beta.lc -0.7829 -0.6050 -0.5106 -0.4143 -0.2414 beta.alt
1.4303 1.7023 1.8535 2.0087 2.3234 beta.lc_modi 0.7307
0.8872 0.9675 1.0515 1.2153 beta.pop_den 0.7658 1.1862
1.4161 1.6625 2.1407

Deviance 833.5560 836.3873 838.5348 841.3827 848.7955

Potential scale reduction factors:

Point est. Upper C.I.
beta.(Intercept) 1.01 1.04
beta.layer.1 1.02 1.09
beta.layer.2 1.00 1.01
beta.layer 1.00 1.00
beta.lc beta.alt 1.00 1.00
beta.lc_modi
beta.pop_den 1.01 1.06
Deviance 1.00 1.01
1.00 1.00
1.01 1.02

Multivariate psrf

1.02

Filoviridae- Binomial iCAR model

Iterations = 5001:9996 Thinning
interval = 5 Number of chains = 2
Sample size per chain = 1000

1. Empirical mean and standard deviation for each variable,
plus standard error of the mean:

Mean SD Naive SE Time-series SE
beta.(Intercept) -68.584 20.47129 0.4578 6.285113
beta.layer.1 184.727 76.96106 1.7209 20.970875
beta.layer.2 -161.274 66.52704 1.4876 17.530246
beta.layer 146.552 56.67100 1.2672 14.934569
beta.lc 26.221 21.29192 0.4761 3.294789
beta.alt 86.114 34.51012 0.7717 5.069096
beta.lc_modi 20.336 9.84002 0.2200 0.985933
beta.pop_den 270.768 183.23397 4.0972 53.637638
gamma.(Intercept) 1.062 0.08945 0.0020 0.002459
Deviance 764.375 4.91207 0.1098 0.291679

2. Quantiles for each variable:

2.5% 25% 50% 75% 97.5%
beta.(Intercept) -122.6578 -77.441 -67.79 -55.346 -36.732
beta.layer.1 74.0126 127.148 162.59 241.215 321.694
beta.layer.2 -278.9760 -212.217 -143.69 -110.954 -64.301
beta.layer 61.0214 105.083 132.25 201.442 243.409
beta.lc -1.9518 10.361 17.84 41.800 69.142 beta.alt
33.5277 58.772 81.14 115.079 152.069
beta.lc_modi 6.2491 12.448 18.53 27.078 42.176 72.5900
beta.pop_den 129.690 186.50 394.954 634.447
gamma.(Intercept) 0.8874 1.003 1.06 1.119 1.241
Deviance 756.7407 760.864 763.77 767.172 775.737

Potential scale reduction factors:

Point est. Upper C.I.
beta.(Intercept) 1.42 2.38
beta.layer.1 3.17 8.29
beta.layer.2 3.15 8.13
beta.layer beta.lc
beta.alt 3.20 7.61
beta.lc_modi 2.51 8.71
beta.pop_den 2.84 6.25
2.16 4.73
2.90 12.15
gamma.(Intercept) 1.00 1.01
Deviance 1.06 1.23

Multivariate psrf **2.17**

Filoviridae- ZIB iCAR model

Iterations = 5001:9996 Thinning interval = 5 Number of chains = 2
Sample size per chain = 1000

1. Empirical mean and standard deviation for each variable, plus standard error of the mean:

	Mean	SD	Naive SE	Time-series SE
beta.(Intercept)	-5.0058	0.34385	0.007689	0.02988
beta.layer.1	2.5048	0.54371	0.012158	0.06129
beta.layer.2	-1.2656	0.42426	0.009487	0.03501
beta.layer	1.4961	0.26798	0.005992	0.02517
beta.lc	-0.6177	0.23187	0.005185	0.01600
beta.alt	1.3166	0.38944	0.008708	0.04137
beta.lc_modi	1.1634	0.23215	0.005191	0.01679
beta.pop_den	1.3672	0.51480	0.011511	0.02824
gamma.(Intercept)	814.8946	620.53149	13.875506	19.42589
Vrho	9.9018	0.07377	0.001649	0.01198
Deviance	267.1366	19.03855	0.425715	1.65344

2. Quantiles for each variable:

	2.5%	25%	50%	75%	97.5%
beta.(Intercept)	-5.6722	-5.2418	-5.0128	-4.7700	-4.3141
beta.layer.1	1.4060	2.1420	2.4936	2.8745	3.6247
beta.layer.2	-2.1314	-1.5475	-1.2646	-0.9791	-0.4775
beta.layer	1.0052	1.3065	1.4921	1.6650	2.0475
beta.lc	-1.0441	-0.7742	-0.6215	-0.4535	-0.1562
beta.alt	0.5948	1.0561	1.3028	1.5745	2.1356
beta.lc_modi	0.7507	0.9962	1.1450	1.3280	1.6362
beta.pop_den	0.4781	1.0035	1.3313	1.7039	2.4967
gamma.(Intercept)	37.1924	339.1505	694.1825	1159.6324	2390.2706
Vrho	9.7477	9.8476	9.9203	9.9646	9.9957
Deviance	231.4583	254.5566	266.4477	279.7336	305.0297

Potential scale reduction factors:

	Point est.	Upper C.I.
beta.(Intercept)	1.12	1.43
beta.layer.1	1.03	1.12
beta.layer.2	1.05	1.22
beta.layer	1.00	1.01
beta.lc	1.02	1.09
beta.alt	1.02	1.02
beta.lc_modi	1.01	1.04
beta.pop_den	1.01	1.03
gamma.(Intercept)	1.00	1.00
Vrho	1.05	1.18

Deviance	1.00	1.00

Multivariate psrf

1.12

	**Model**	**Deviance**	**Perc**
1	NULL	1891.4073	0
2	env	764.3752	69
3	binomial	839.2431	65
4	binomial.icar	268.5234	100
5	ZIB.icar	267.1366	100

**"masTSS"**	**"OA"**	**"prob"**
**0.99**	**1**	**0.66**

Coronaviridae- Binomial model

Iterations = 5001:9996 Thinning
interval = 5 Number of chains = 2
Sample size per chain = 1000

1. Empirical mean and standard deviation for each variable,
   plus standard error of the mean:

	Mean	SD	Naive SE	Time-series SE
beta.(Intercept)	-2.1638	0.2205	0.004929	0.015419
beta.layer.1	3.7979	0.8500	0.019007	0.141365
beta.layer.2	-0.6954	0.6879	0.015382	0.106804
beta.layer	-0.7493	0.2248	0.005027	0.028991
beta.lc	-0.3097	0.1591	0.003557	0.006269 2.3602
beta.alt		0.2213	0.004948	0.025580
beta. lc_ modi	2.5148	0.2299	0.005142	0.011551
beta. pop_ den	2.5332	0.3239	0.007244	0.011723
Deviance 629.1162		4.1161	0.092039	0.156460

2. Quantiles for each variable:

	2.5%	25%	50%	75%
beta.(Intercept)	-2.6155	-2.3058	-2.1630	-2.0255
beta.layer.1	2.0819	3.2633	3.8563	4.3605
beta.layer.2	-1.9724	-1.1275	-0.7602	-0.2885
beta.layer	-1.1868	-0.9021	-0.7542	-0.5926
beta.lc	-0.6217	-0.4195	-0.3057	-0.1974 1.9256
beta.alt		2.2091	2.3555	2.5068
beta.lc_modi	2.0629	2.3564	2.5175	2.6632
beta.pop_den	1.9309	2.3045	2.5273	2.7513
Deviance	623.3048	626.1436	628.5594	631.3158

	97.5%
beta.(Intercept)	-1.73494
beta.layer.1	5.34437
beta.layer.2	0.76732
beta.layer	-0.31073
beta.lc	-0.01345
beta.alt	2.77599
beta.lc_modi	3.00822
beta.pop_den	3.19233 Deviance
638.75865	

Potential scale reduction factors:

	Point est.	Upper C.I.
beta.(Intercept)	1.02	1.09
beta.layer.1	1.11	1.42
beta.layer.2	1.09	1.35
beta.layer	1.06	1.24

beta.lc	1.00	1.01
beta.alt	1.04	1.18
beta.lc_modi	1.01	1.07
beta.pop_den	1.00	1.00
Deviance	1.01	1.04

Multivariate psrf

1.07

Coronaviridae- ZIB model

Iterations = 10001:19991 Thinning
interval = 10 Number of chains = 2
Sample size per chain = 1000

1. Empirical mean and standard deviation for each variable,
plus standard error of the mean:

	Mean	SD	Naive SE	Time-series SE
beta.(Intercept)	-224.637	88.0989	1.969952	5.998e+01
beta.layer.1	255.155	125.6389	2.809370	6.025e+ 01
beta.layer.2	124.343	98.5342	2.203292	3.987e+ 01
beta.layer	-23.198	19.6178	0.438668	5.455e+00
beta.lc	1.894	8.3197	0.186035	1.131e+00
beta.alt	253.489	95.1583	2.127805	5.831e+01
beta.lc_modi	425.926	161.5253	3.611816	1.114e+02
beta.pop_den	29.792	45.2351	1.011489	8.569e+00
gamma.(Intercept)	1.582	0.1125	0.002515	2.656e-03
Deviance	548.472	5.8761	0.131393	6.678e-01

2. Quantiles for each variable:

	2.5%	25%	50%	75%
beta.(Intercept)	-414.5339	-260.954	-224.749	-166.357
beta.layer.1	52.8548	158.580	259.036	348.230
beta.layer.2	0.6359	48.964	92.950	180.589
beta.layer	-63.0630	-37.165	-21.297	-6.601
beta.lc	-15.3978	-3.038	1.023	7.661
beta.alt	97.7189	193.783	260.491	300.421
beta.lc_modi	166.9442	333.225	436.017	495.776
beta.pop_den	-12.3879	1.657	11.688	38.266
gamma.(Intercept)	1.3712	1.508	1.581	1.658
Deviance	537.3869	544.428	547.923	552.011

beta.(Intercept) -89.949
beta.layer.1 485.966
beta.layer.2 386.439
beta.layer 4.337
beta.lc17.872
beta.alt445.758
beta.lc_modi 759.543
beta.pop_den 164.533 gamma.
(Intercept) 1.802 Deviance
561.017

Potential scale reduction factors:

	Point est.	Upper C.I.
beta.(Intercept)	1.25	1.30

beta.layer.1	1.29	1.98
beta.layer.2	1.14	1.47
beta.layer	1.25	1.83
beta.lc	1.11	1.36
beta.alt	1.26	1.82
beta.lc_modi	1.25	1.54
beta.pop_den	1.42	3.32
gamma.(Intercept)	1.01	1.04
Deviance	1.06	1.24

Multivariate psrf

1.38

Coronaviridae- Binomial iCAR model

Iterations = 5001:9996 Thinning
interval = 5 Number of chains = 2
Sample size per chain = 1000

1. Empirical mean and standard deviation for each variable,
   plus standard error of the mean:

Mean SD Naive SE Time-series SE
beta.(Intercept) -5.8993 0.60388 0.013503 0.054930
beta. layer. 1 3.1738 1.99638 0.044640 0.388036
beta. layer. 2 -0.1431 1.72627 0.038600 0.302624
beta. layer -1.4755 0.57547 0.012868 0.069770
beta.lc -0.1733 0.42810 0.009573 0.022948 2.0431
beta.alt 0.58802 0.013149 0.055892
beta. lc_ modi 2.5623 0.58561 0.013095 0.040325
beta. pop_ den 3.3864 0.90664 0.020273 0.080053
Vrho9.9211 0.04686 0.001048 0.007787
Deviance 63.0917 10.20057 0.228092 0.805057

2. Quantiles for each variable:

2.5% 25% 50% 75% 97.5%beta.(Intercept)
-7.057 -6.2937 -5.9007 -5.4734 -4.7317 beta.layer.1 -1.037
1.8136 3.3688 4.6736 6.4875 beta.layer.2 -3.173 -1.4631
-0.1816 1.0283 3.2976 beta.layer -2.615 -1.8632 -1.4828
-1.0505 -0.4358 beta.lc -1.059 -0.4522 -0.1680 0.1204
0.6530 beta.alt 0.837 1.6606 2.0643 2.4616 3.1318
beta.lc_modi 1.497 2.1540 2.5237 2.9314 3.7982
beta.pop_den 1.745 2.7541 3.3471 3.9601 5.1832

Vrho9.839 9.8821 9.9220 9.9619 9.9956
Deviance 44.653 55.9676 62.6950 69.8488 83.9747

Potential scale reduction factors:

Point est. Upper C.I.
beta.(Intercept) 1.09 1.34
beta.layer.1 1.01 1.02
beta.layer.2 1.01 1.02
beta.layer 1.10 1.38
beta.lc 1.01 1.03
beta.alt 1.04 1.16
beta.lc_modi 1.22 1.73
beta.pop_den 1.44 2.30
Vrho Deviance 1.01 1.06
1.05 1.18

Multivariate psrf 1.4

Coronaviridae- ZIB iCAR model

Iterations = 10001:19991 Thinning
interval = 10 Number of chains = 2
Sample size per chain = 1000

1. Empirical mean and standard deviation for each variable,
   plus standard error of the mean:

Mean SD Naive SE beta.
(Intercept) -6.257 0.62276 0.013925 beta.layer.1
4.312 2.63169 0.058846

beta.layer.2 -0.990 2.19117 0.048996

beta.layer -1.849 0.72472 0.016205 beta.lc
-0.262 0.44891 0.010038
beta.alt 2.206 0.65840 0.014722
beta.lc_modi 2.522 0.56494 0.012632 beta.pop_den
3.755 0.83355 0.018639 gamma.(Intercept) 776.973
587.09236 13.127784

Vrho Deviance 9.919 0.04903 0.001096
59.005 10.23167 0.228787
Time-series SE

beta.(Intercept) 0.044082
beta.layer.1 0.532657
beta.layer.2 0.382394
beta.layer beta.lc 0.093402
beta.alt 0.023349
beta.lc_modi 0.099156
beta.pop_den

0.030517
0.059244

gamma.(Intercept) 14.200550
Vrho 0.006272
Deviance 0.721394

2. Quantiles for each variable:

	2.5%	25%	50%	75%
beta.(Intercept)	-7.5764	-6.6587	-6.2157	-5.81767
beta.layer.1	-1.0942	2.7539	4.3463	5.92397
beta.layer.2	-5.0734	-2.4187	-0.9744	0.33959
beta.layer	-3.2070	-2.3339	-1.8875	-1.36643
beta.lc	-1.1423	-0.5646	-0.2654	0.02459 0.9472
beta.alt	1.7687	2.2031	2.63256	
beta.lc_modi	1.4749	2.1512	2.5048	2.90040
beta.pop_den	2.1531	3.1863	3.7472	4.30956
gamma.(Intercept)	38.1536	320.4527	639.2747	1123.56527
Vrho	9.8351	9.8755	9.9217	9.96113
Deviance	40.7212	51.9276	58.3373	65.39818

	97.5%
beta.(Intercept)	-5.1221
beta.layer.1	9.3579
beta.layer.2	3.5601
beta.layer	-0.3798

beta.lc 0.6421
beta.alt 3.5145
beta.lc_modi 3.6880
beta.pop_den 5.4344
gamma.(Intercept) 2115.2218
Vrho 9.9968
Deviance 81.1050

Potential scale reduction factors:

Point est. Upper C.I.
beta.(Intercept) 1.03 1.11
beta.layer.1 1.05 1.05
beta.layer.2 1.05 1.07
beta.layer beta.lc 1.02 1.10
beta.alt
beta.lc_modi 1.00 1.00
beta.pop_den 1.01 1.02
1.03 1.15
1.01 1.03
gamma.(Intercept) 1.00 1.00
Vrho Deviance 1.00 1.02
Multivariate psrf 1.00 1.00

1.17

	Model	Deviance	Perc
1	NULL	1889.17448	0
2	env	548.47170	74
3	binomial	629.11621	69
4	binomial.icar	63.09174	100
5	ZIB.icar	67.01016	100

"masTSS"	"OA"	"prob"
1	1	0.65

Henipavirus- Binomial model

Iterations = 5001:9996 Thinning
interval = 5 Number of chains = 2
Sample size per chain = 1000

1. Empirical mean and standard deviation for each variable,
plus standard error of the mean:

Mean SD Naive SE Time-series SE
beta.(Intercept) -7.42157 0.91838 0.020536 0.148278
beta.layer.1 -2.72411 1.60108 0.035801 0.270781
beta.layer.2 1.65097 1.30463 0.029172 0.215200
beta.layer 2.23412 0.39907 0.008923 0.046655
beta.lc 0.21787 0.28018 0.006265 0.016682
beta.alt -7.12888 1.92968 0.043149 0.308019
beta. lc_ modi 2.01717 0.38696 0.008653 0.026624
beta.pop_den 0.00701 0.15813 0.003536 0.007293 Vrho
9.87936 0.05526 0.001236 0.008422 Deviance 113.44166
12.32776 0.275657 0.945002

2. Quantiles for each variable:

2.5% 25% 50% 75% 97.5%
beta.(Intercept) -9.4726 -8.00068 -7.29651 -6.8059 -5.8840
beta.layer.1 -5.7679 -3.82144 -2.80242 -1.6734 0.6095
beta.layer.2 -1.1103 0.82618 1.65468 2.5389 4.1226
beta.layer 1.4294 1.96110 2.23344 2.5053 3.0142 -0.3397
beta.lc 0.03295 0.21572 0.4036 0.7808
beta.alt -11.3713 -8.22921 -7.05425 -5.7307 -3.9039
beta.lc_modi 1.2865 1.75444 2.03407 2.2744 2.7566
beta.pop_den -0.3128 -0.09765 0.01136 0.1141 0.2983
Vrho 9.7971 9.83454 9.86940 9.9186 9.9928
Deviance 90.6080 104.71929 112.90177 122.1287 138.1282

Potential scale reduction factors:

Point est. Upper C.I.
beta.(Intercept) 1.13 1.46
beta.layer.1 1.08 1.31
beta.layer.2 1.11 1.42
beta.layer 1.00 1.01
beta.lc 1.06 1.25
beta.alt 1.10 1.36
beta.lc_modi 1.33 2.03
beta.pop_den 1.02 1.10
Vrho Deviance 1.10 1.37
1.09 1.33

Multivariate psrf **1.57**

Henipavirus- ZIB model

Iterations = 5001:9996 Thinning
interval = 5 Number of chains = 2
Sample size per chain = 1000

1. Empirical mean and standard deviation for each variable, plus standard error of the mean:

	Mean	SD	Naive SE	Time-series SE
beta.(Intercept)	-6.1898	0.7317	0.016362	0.133905
beta.layer.1	-1.7241	0.6138	0.013725	0.060312
beta.layer.2	0.9176	0.5074	0.011346	0.042359
beta.layer	1.3295	0.1863	0.004165	0.012484
beta.lc	0.2903	0.1405	0.003141	0.005599
beta.alt	-11.7266	1.5390	0.034413	0.301332
beta. lc_ modi	1.2574	0.1581	0.003536	0.005924
beta. pop_ den	0.1164	0.1256	0.002808	0.003849
Deviance	635.2810	4.0968	0.091607	0.197628

2. Quantiles for each variable:

	2.5%	25%	50%	75%	97.5%
beta.(Intercept)	-7.77453	-6.59967	-6.1525	-5.7043	-4.9017
beta.layer.1	-2.91079	-2.14856	-1.7303	-1.3177	-0.5201
beta.layer.2	-0.08586	0.59115	0.9290	1.2549	1.9092
beta.layer	0.96104	1.20545	1.3335	1.4542	1.6975
beta.lc	0.01660	0.19248	0.2918	0.3849	0.5634
beta.alt	-15.08253	-12.66530	-11.6315	-10.6695	-9.0201
beta.lc_modi	0.94251	1.15159	1.2567	1.3619	1.5712
beta.pop_den	-0.09433	0.02814	0.1078	0.1918	0.3981
Deviance	629.32335	632.25033	634.6044	637.5753	645.0544

Potential scale reduction factors:

	Point est.	Upper C.I.
beta.(Intercept)	1.02	1.11
beta.layer.1	1.02	1.08
beta.layer.2	1.02	1.07
beta.layer	1.01	1.05
beta.lc	1.00	1.02
beta.alt	1.02	1.08
beta.lc_modi	1.00	1.00
beta.pop_den	1.00	1.00
Deviance	1.00	1.00

Multivariate psrf

1.04

Henipavirus- Binomial iCAR model

Iterations = 5001:9996 Thinning
interval = 5 Number of chains = 2
Sample size per chain = 1000

1. Empirical mean and standard deviation for each variable,
plus standard error of the mean:

	Mean	SD	Naive SE	Time-series SE
beta.(Intercept)	-5.7849	0.8117	0.018151	0.157069
beta.layer.1	-1.2758	0.6525	0.014590	0.067635
beta.layer.2	0.2252	0.6628	0.014820	0.084433
beta.layer	1.2112	0.2052	0.004588	0.017493
beta.lc	0.3060	0.1564	0.003498	0.006503
beta.alt	-11.6670	1.3888	0.031054	0.238654
beta.lc_modi	0.7398	0.3380	0.007558	0.045304
beta.pop_den	1.7751	1.2008	0.026851	0.197678
gamma.(Intercept)	6.9297	10.3305	0.230997	2.579533
Deviance	627.4858	4.7735	0.106739	0.475115

2. Quantiles for each variable:

	2.5%	25%	50%	75%	97.5%
beta.(Intercept)	-7.27209	-6.3586	-5.8325	-5.2327	-4.25214
beta.layer.1	-2.56459	-1.7053	-1.2951	-0.8435	0.06333
beta.layer.2	-1.14806	-0.1876	0.2455	0.7096	1.40811
beta.layer	0.79719	1.0828	1.2055	1.3445	1.60444
beta.lc	0.01016	0.2008	0.3048	0.4091	0.61377
beta.alt	-14.60397	-12.5680	-11.6711	-10.6569	-9.26813
beta.lc_modi	0.03019	0.5245	0.7775	0.9734	1.32638
beta.pop_den	0.10213	0.9022	1.3065	2.6855	4.41259
gamma.(Intercept)	2.69122	3.4291	4.0987	5.2802	47.80220
Deviance	620.68101	624.0309	626.6045	630.1071	639.03774

Potential scale reduction factors:

	Point est.	Upper C.I.
beta.(Intercept)	1.01	1.04
beta.layer.1	1.01	1.03
beta.layer.2	1.02	1.08
beta.layer beta.lc	1.00	1.01
beta.alt		
beta.lc_modi	1.01	1.03
beta.pop_den	1.01	1.01
	1.03	1.14
	1.02	1.10
gamma.(Intercept)	1.36	3.62
Deviance	1.04	1.12

Multivariate psrf **1.08**

Henipavirus- ZIB iCAR model

Iterations = 5001:9996 Thinning
interval = 5 Number of chains = 2
Sample size per chain = 1000

1. Empirical mean and standard deviation for each variable,
plus standard error of the mean:

Mean SD Naive SE Time-series SE
beta.(Intercept) -8.271041 1.10538 0.024717 0.245798
beta.layer.1 -3.651963 1.37054 0.030646 0.196258
beta.layer.2 2.353747 1.13634 0.025409 0.176039
beta.layer 2.413694 0.41139 0.009199 0.040010
beta.lc0.291016 0.26851 0.006004 0.015301
beta.alt -9.052617 2.42205 0.054159 0.534230
beta.lc_modi 2.135840 0.36923 0.008256 0.025867
beta.pop_den 0.003795 0.15525 0.003472 0.006192
gamma.(Intercept) 817.584802 628.33554 14.050010 18.905331 Vrho
9.881421 0.05772 0.001291 0.009663 Deviance 116.763581
12.93415 0.289216 0.828173

2. Quantiles for each variable:

2.5%25% 50% 75%
beta.(Intercept) -11.53154 -8.83573 -8.194535 -7.5847
beta.layer.1 -6.26383 -4.49145 -3.666831 -2.8802
beta.layer.2 -0.02205 1.66728 2.354603 3.0980
beta.layer 1.61763 2.13063 2.427338 2.7051 -0.23010
beta.lc 0.11038 0.289858 0.4652
beta.alt -16.27392 -10.31546 -8.840097 -7.5964
beta.lc_modi 1.41220 1.88832 2.125123 2.3858
beta.pop_den -0.30820 -0.09714 0.003661 0.1105
gamma.(Intercept) 51.19336 321.06399 676.766416 1198.1010
Vrho Deviance 9.79906 9.83378 9.867952 9.9227
93.46029 107.92418 115.857036 124.5996

97.5%
beta.(Intercept) -6.3486
beta.layer.1 - 0.6572
beta.layer.2 4.5281
beta.layer 3.1192
beta.lc 0.8251
beta.alt -4.9500
beta.lc_modi 2.8822
beta.pop_den 0.3041
gamma.(Intercept) 2297.7514
Vrho 9.9944
Deviance 144.9087

Potential scale reduction factors:

	Point est.	Upper C.I.
beta.(Intercept)	1.10	1.10
beta.layer.1	1.11	1.25
beta.layer.2	1.06	1.15
beta.layer		
beta.lc	1.20	1.38
beta.alt	1.01	1.01
beta.lc_modi	1.10	1.14
beta.pop_den	1.08	1.20
	1.02	1.08
gamma.(Intercept)	1.00	1.00
Vrho Deviance	1.18	1.33
**Multivariate psrf**	1.07	1.27

1.18

	**Model**	**Deviance**	**Perc**
1	NULL	1862.7433	0
2	env	627.4858	71
3	binomial	635.2810	70
4	binomial.icar	113.4417	100
5	ZIB.icar	116.7636	100

**"masTSS"**	**"OA"**	**"prob"**
0.99	1	0.48

Figure S2: True Skill Statistic (TSS) plots

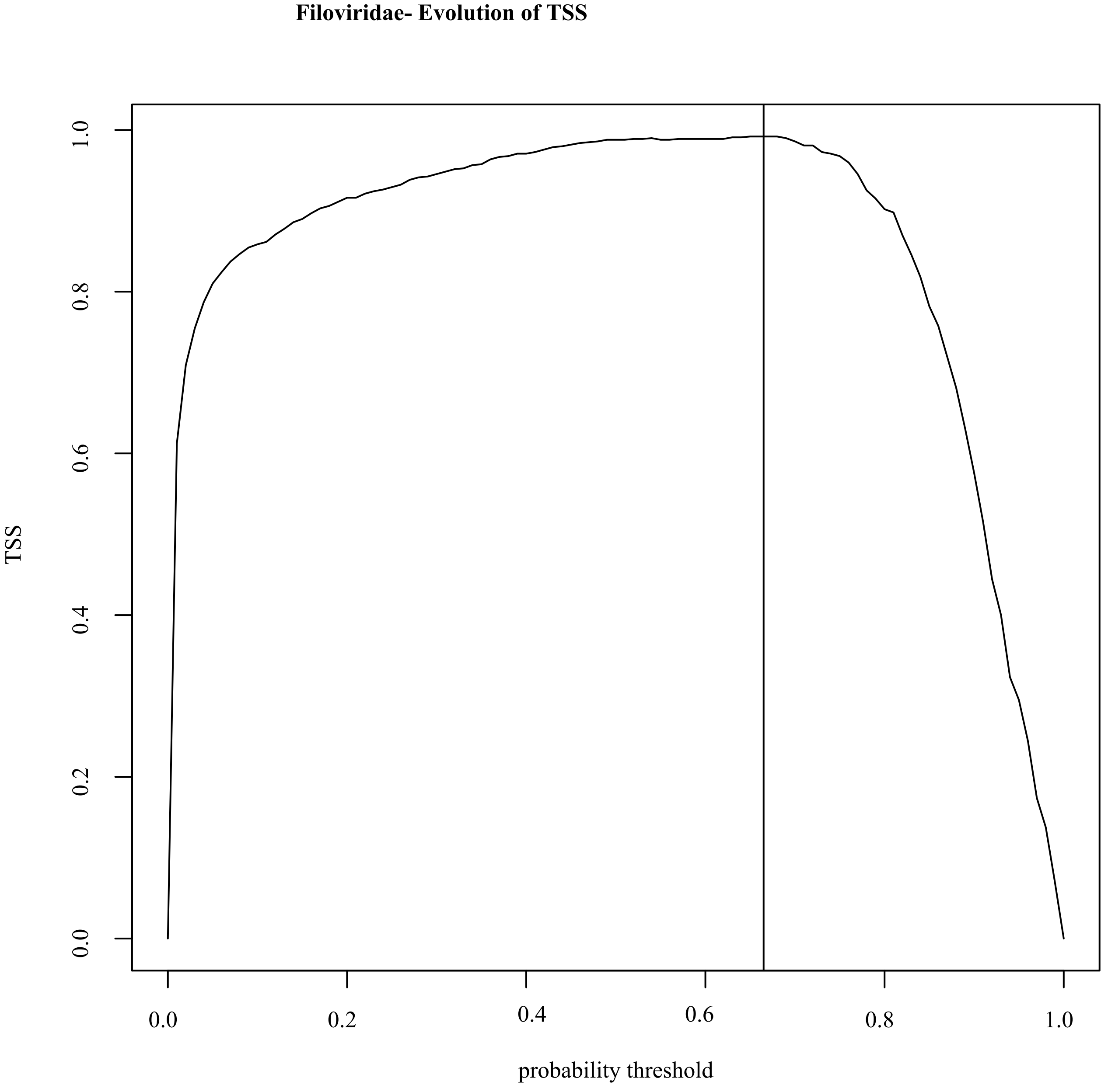

Figure S2: True Skill Statistic (TSS) plots

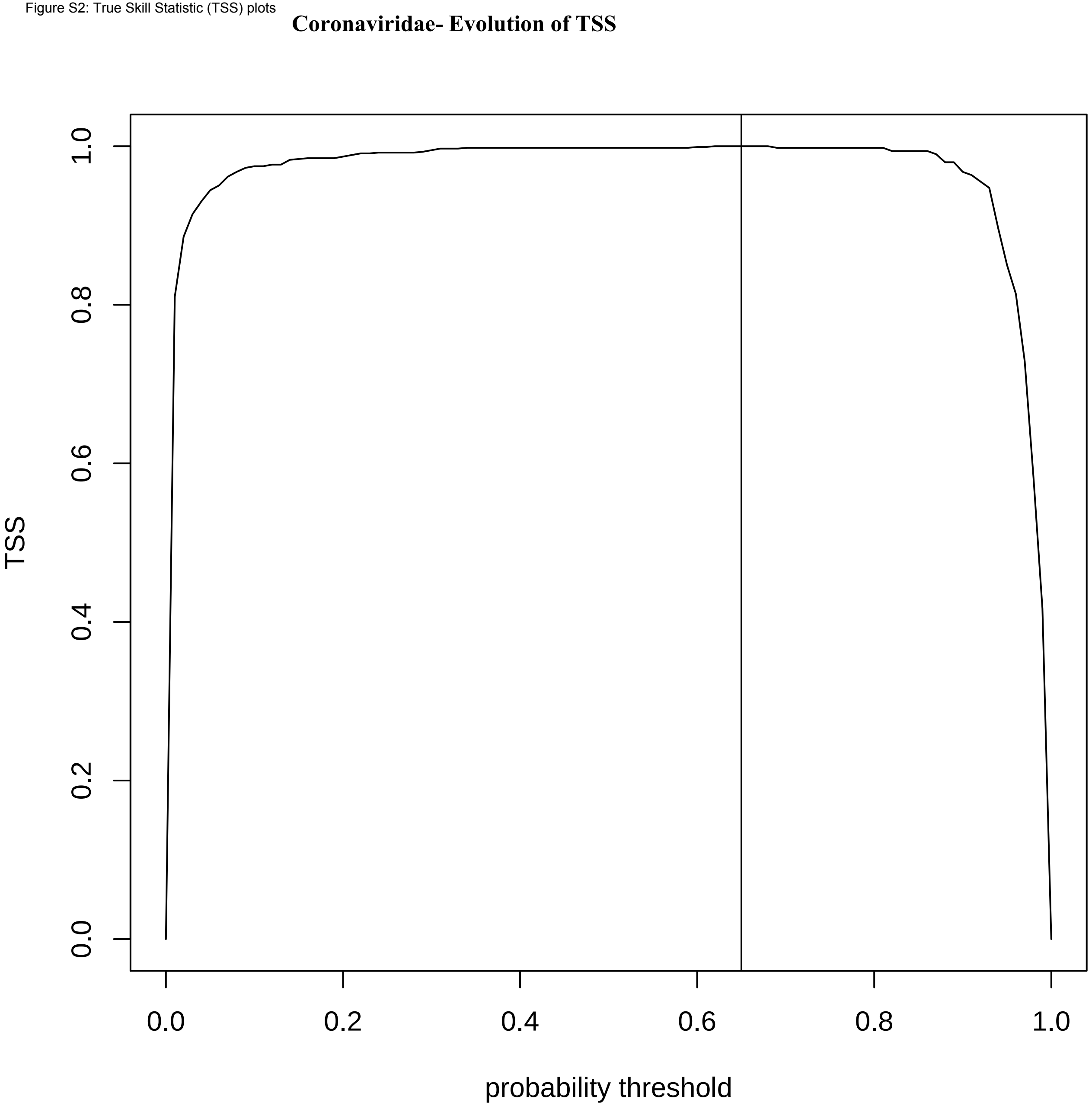

Figure S2: True Skill Statistic (TSS) plots

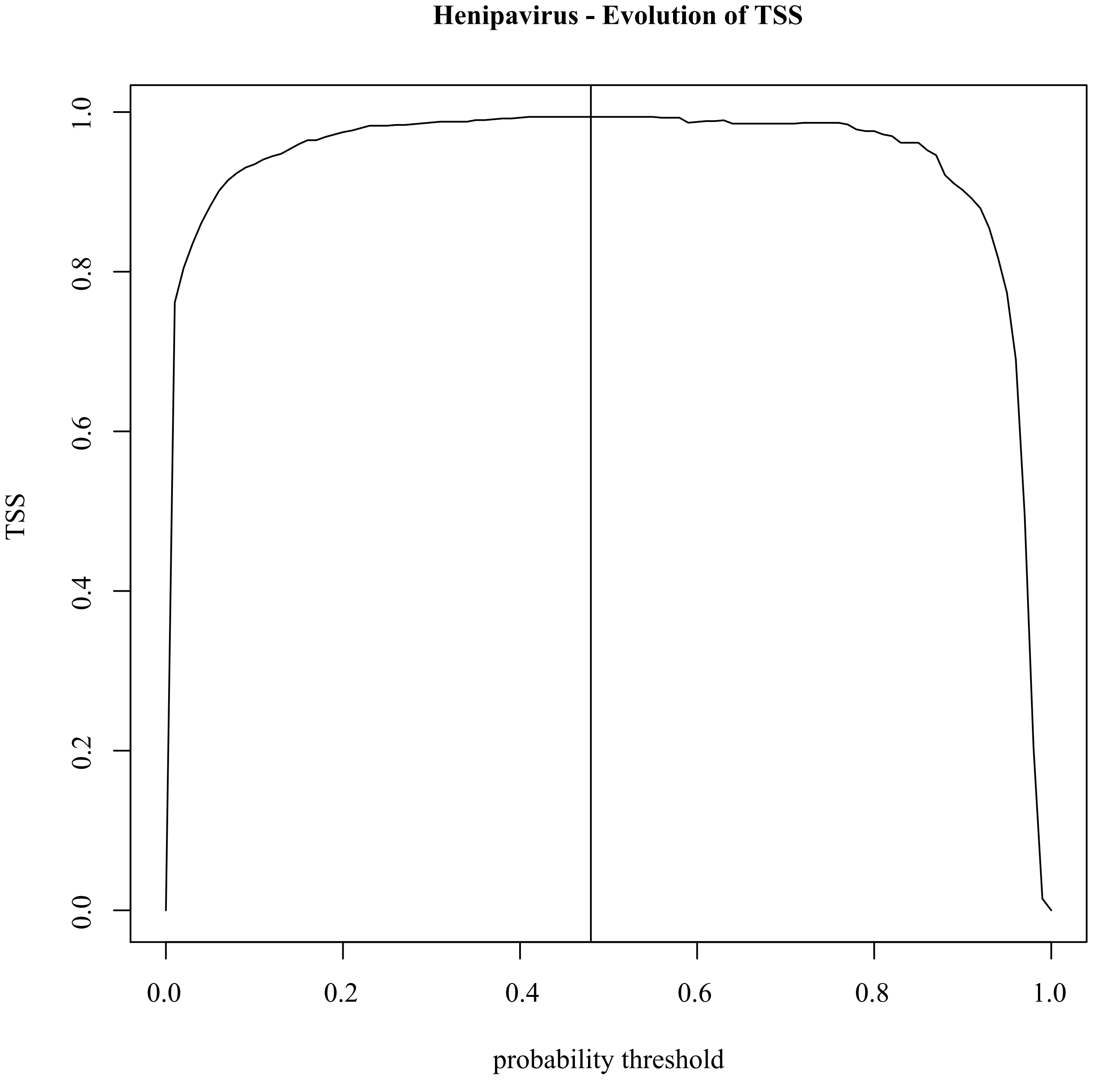

Figure S3: ZIB
iCAR random effects

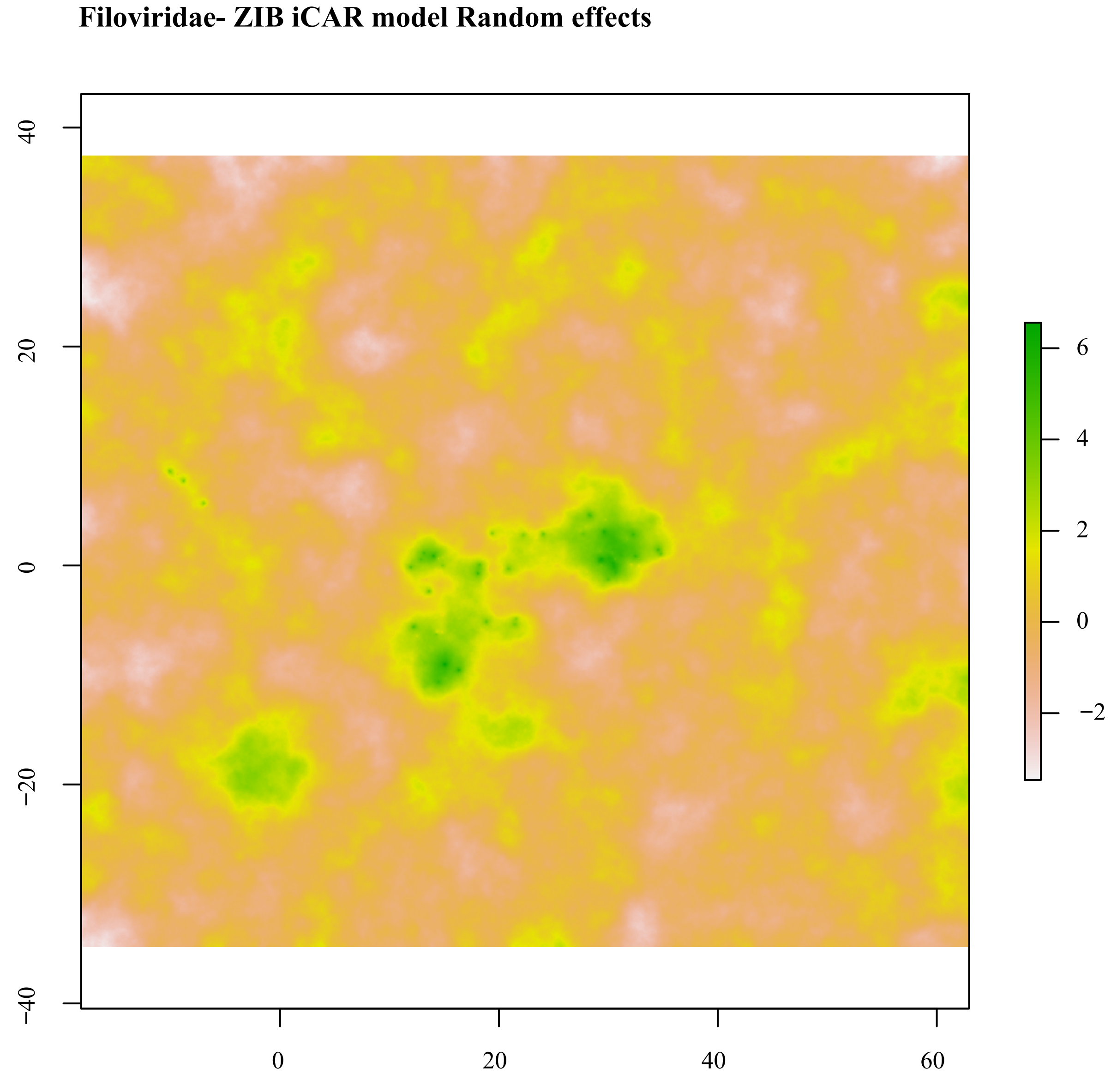

Figure S3: ZIB
iCAR random effects

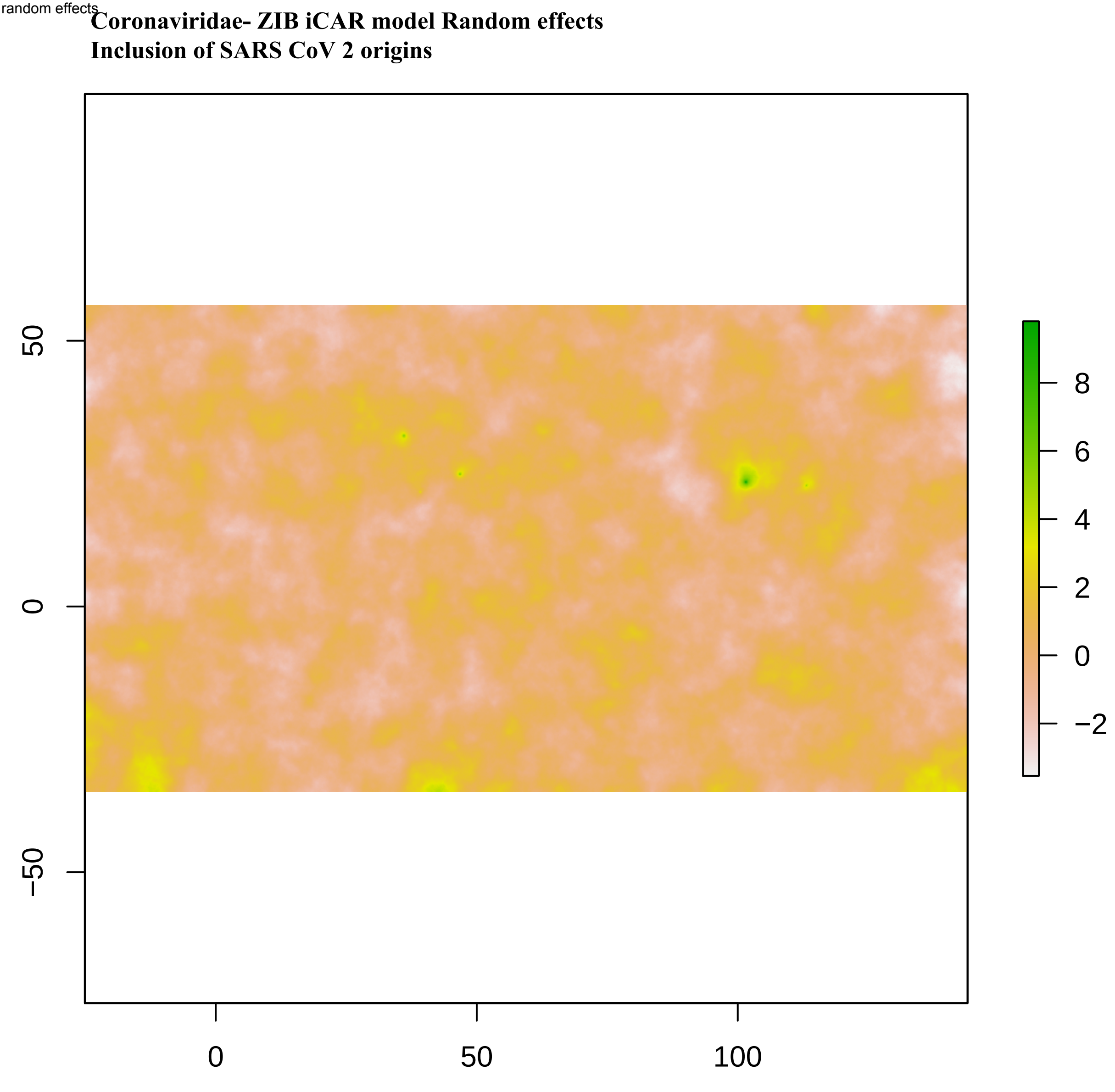

Figure S3: ZIB
iCAR random effects

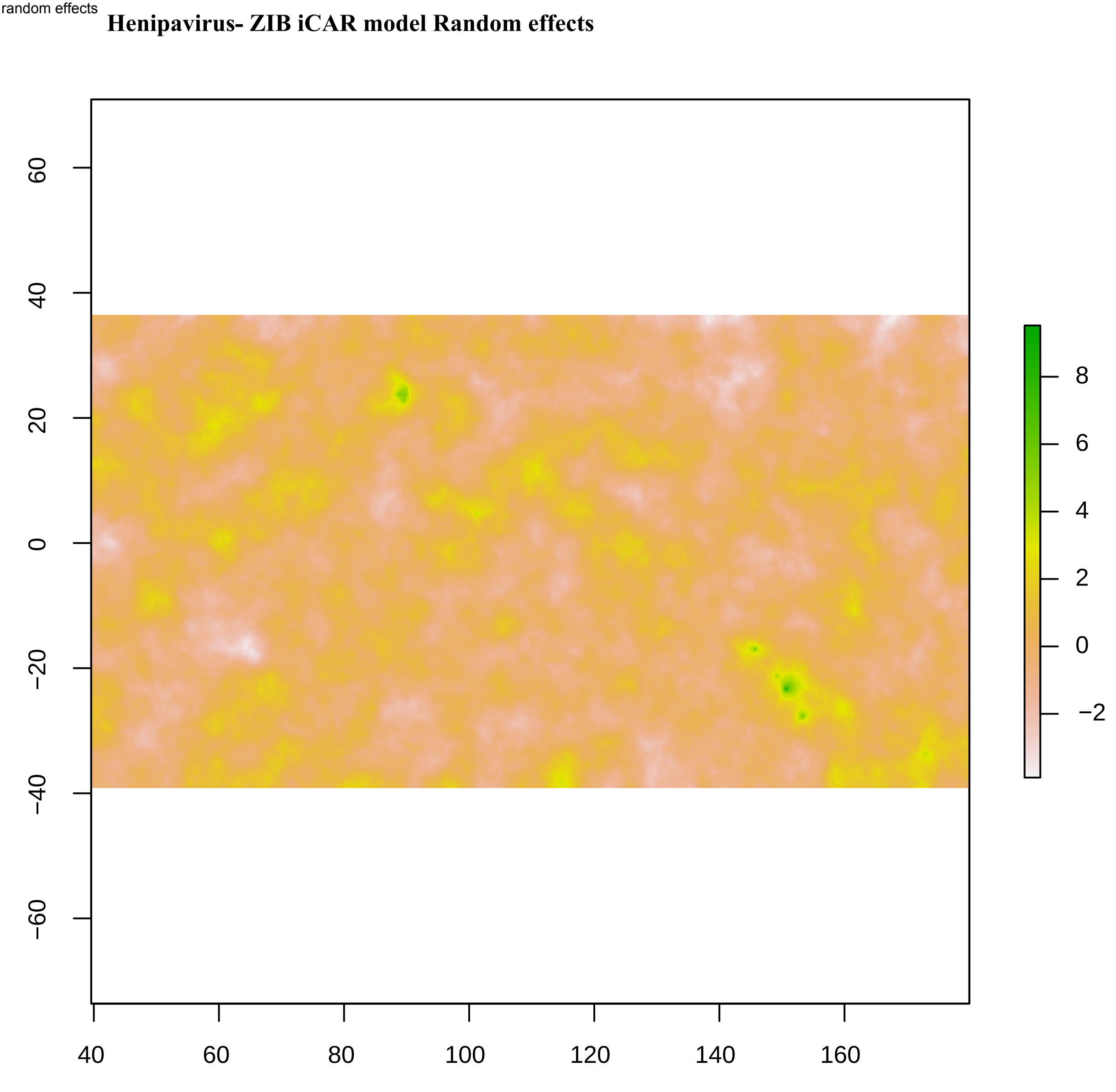

Figure S4: Significant hotspots based on TSS

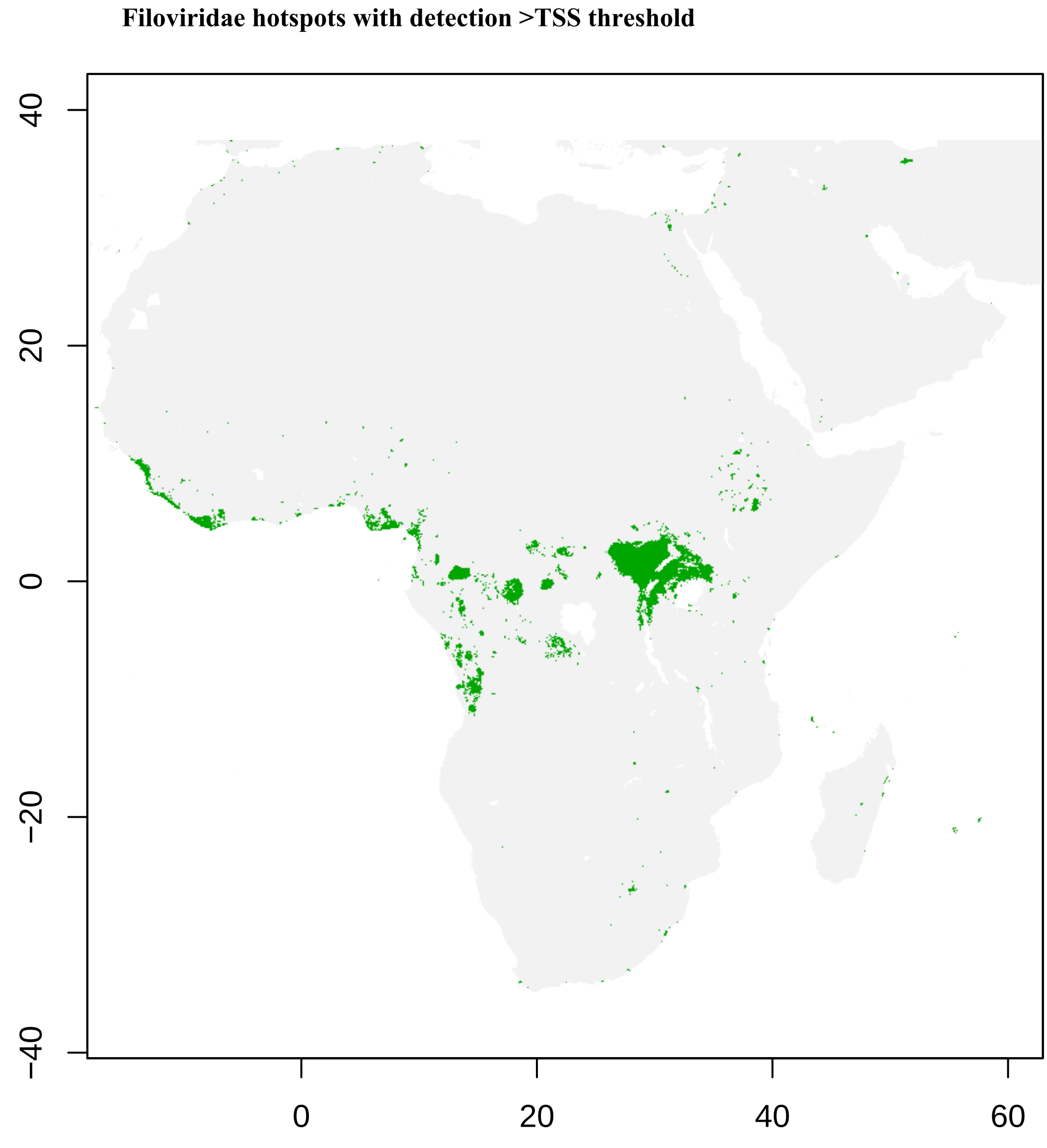

Figure S4: Significant hotspots based on TSS

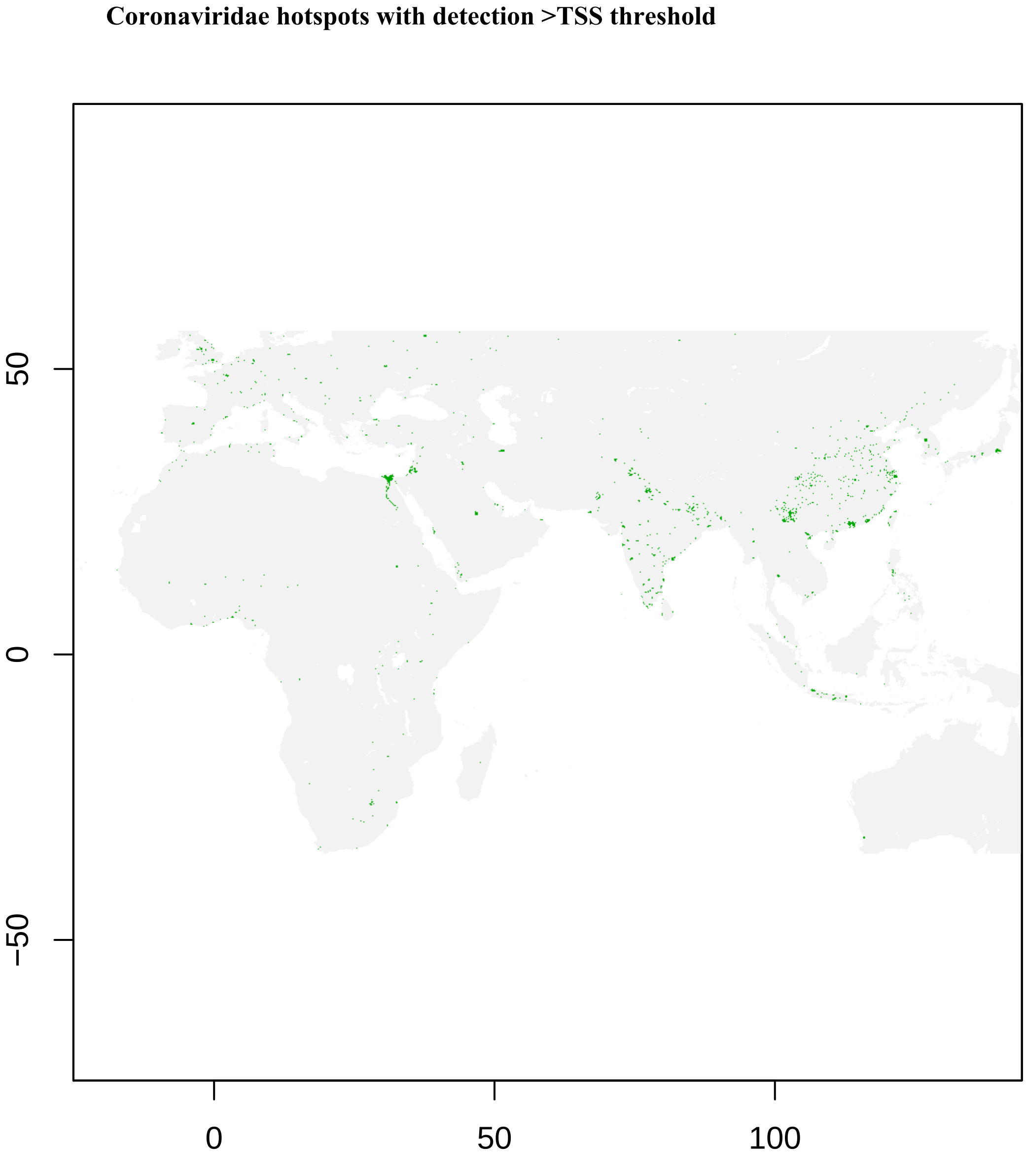

Figure S4: Significant hotspots based on TSS

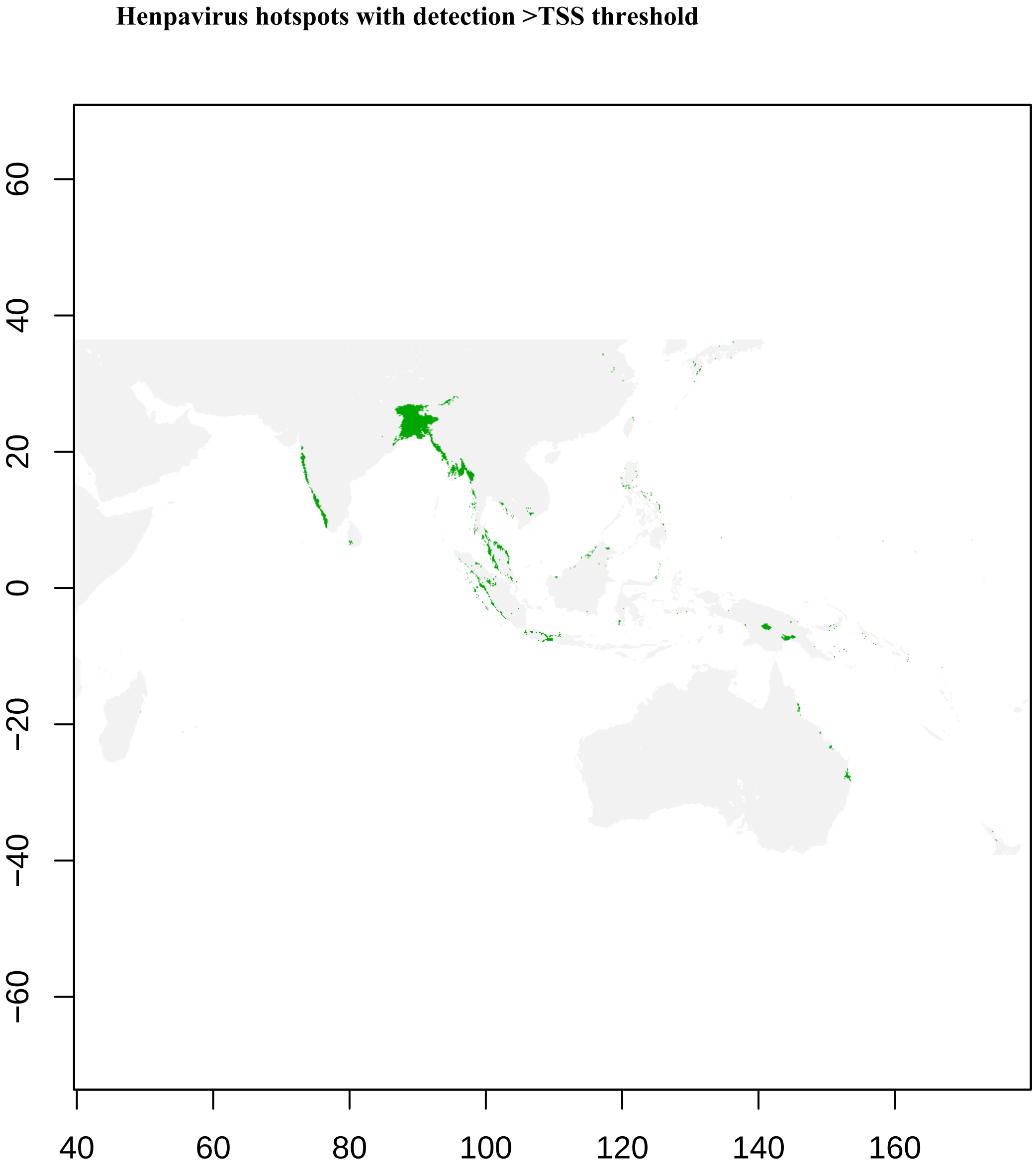

Supplementary Table 1: Reservoirs of diseases analyzed in the study

Virus	Reservoir	Binomial
MERS	serotine bat	Neoromicia_zuluensis
MERS	Egyptian tomb bat	Taphozous_perforatus
MERS	bat	Rhinopoma_microphyllum
MERS	bat	Pipistrellus_kuhlii
MERS	bat	Eptesicus_bottae
MERS	bat	Eidolon_helvum
MERS	bat	Rousettus_aegyptiacus
Henipavirus	fruit bats/ flying foxes	Pteropus_anetianus
Henipavirus	fruit bats/ flying foxes	Pteropus_aruensis
Henipavirus	fruit bats/ flying foxes	Pteropus_caniceps
Henipavirus	fruit bats/ flying foxes	Pteropus_capistratus
Henipavirus	fruit bats/ flying foxes	Pteropus_chrysoproctus
Henipavirus	fruit bats/ flying foxes	Pteropus_cognatus
Henipavirus	fruit bats/ flying foxes	Pteropus_conspicillatus
Henipavirus	fruit bats/ flying foxes	Pteropus_dasymallus
Henipavirus	fruit bats/ flying foxes	Pteropus_faunulus
Henipavirus	fruit bats/ flying foxes	Pteropus_fundatus
Henipavirus	fruit bats/ flying foxes	Pteropus_giganteus
Henipavirus	fruit bats/ flying foxes	Pteropus_gilliardorum
Henipavirus	fruit bats/ flying foxes	Pteropus_griseus
Henipavirus	fruit bats/ flying foxes	Pteropus_howensis
Henipavirus	fruit bats/ flying foxes	Pteropus_hypomelanus

Henipavirus	fruit bats/ flying foxes	Pteropus_intermedius
Henipavirus	fruit bats/ flying foxes	Pteropus_keyensis
Henipavirus	fruit bats/ flying foxes	Pteropus_leucopterus
Henipavirus	fruit bats/ flying foxes	Pteropus_livingstonii
Henipavirus	fruit bats/ flying foxes	Pteropus_lombocensis
Henipavirus	fruit bats/ flying foxes	Pteropus_loochoensis
Henipavirus	fruit bats/ flying foxes	Pteropus_lylei
Henipavirus	fruit bats/ flying foxes	Pteropus_macrotis
Henipavirus	fruit bats/ flying foxes	Pteropus_mahaganus
Henipavirus	fruit bats/ flying foxes	Pteropus_mariannus
Henipavirus	fruit bats/ flying foxes	Pteropus_melanopogon
Henipavirus	fruit bats/ flying foxes	Pteropus_melanotus
Henipavirus	fruit bats/ flying foxes	Pteropus_molossinus
Henipavirus	fruit bats/ flying foxes	Pteropus_neohibernicus
Henipavirus	fruit bats/ flying foxes	Pteropus_niger
Henipavirus	fruit bats/ flying foxes	Pteropus_nitendiensis
Henipavirus	fruit bats/ flying foxes	Pteropus_ocularis
Henipavirus	fruit bats/ flying foxes	Pteropus_ornatus
Henipavirus	fruit bats/ flying foxes	Pteropus_pelewensis
Henipavirus	fruit bats/ flying foxes	Pteropus_personatus
Henipavirus	fruit bats/ flying foxes	Pteropus_pohlei
Henipavirus	fruit bats/ flying foxes	Pteropus_poliocephalus
Henipavirus	fruit bats/ flying foxes	Pteropus_pselaphon
Henipavirus	fruit bats/ flying foxes	Pteropus_pumilus

Henipavirus	fruit bats/ flying foxes	Pteropus_rayneri
Henipavirus	fruit bats/ flying foxes	Pteropus_rennelli
Henipavirus	fruit bats/ flying foxes	Pteropus_rodricensis
Henipavirus	fruit bats/ flying foxes	Pteropus_rufus
Henipavirus	fruit bats/ flying foxes	Pteropus_samoensis
Henipavirus	fruit bats/ flying foxes	Pteropus_scapulatus
Henipavirus	fruit bats/ flying foxes	Pteropus_seychellensis
Henipavirus	fruit bats/ flying foxes	Pteropus_speciosus
Henipavirus	fruit bats/ flying foxes	Pteropus_temminckii
Henipavirus	fruit bats/ flying foxes	Pteropus_tonganus
Henipavirus	fruit bats/ flying foxes	Pteropus_tuberculatus
Henipavirus	fruit bats/ flying foxes	Pteropus_ualanus
Henipavirus	fruit bats/ flying foxes	Pteropus_vampyrus
Henipavirus	fruit bats/ flying foxes	Pteropus_vetulus
Henipavirus	fruit bats/ flying foxes	Pteropus_voeltzkowi
Henipavirus	fruit bats/ flying foxes	Pteropus_woodfordi
EVD	Greater Long-fingered bat	Miniopterus_inflatus
EVD	Franquet's epauletted fruit bat	Epomops_franqueti
EVD	hammer-headed fruit bat	Hypsignathus_monstrosus
EVD	little collared fruit bats	Myonycteris_torquata
MVD	Egyptian fruit bat	Rousettus_aegyptiacus
SARS	Greater horseshoe bat	Rhinolophus_ferrumequinum
SARS	civet	Paguma_larvata

Coronaviridae- Binomial model
Inclusion of SARS CoV-2 origin

Iterations = 5001:9996 Thinning
interval = 5 Number of chains = 2
Sample size per chain = 1000

1. Empirical mean and standard deviation for each variable, plus standard error of the mean:

	Mean	SD	Naive SE	Time-series SE	
beta.(Intercept)	-4.7390	0.28041	0.0062701	0.034669	
beta.layer.1	3.5341	0.97653	0.0218358	0.169654	
beta.layer.2	-2.4537	0.86143	0.0192622	0.146552	
beta.layer	-0.9982	0.45390	0.0101494	0.058780	0.4306
beta.lc	0.27230	0.0060888	0.015987	0.7419	0.33981
beta.alt	0.0075984	0.032927			
beta. lc_ modi	1.2297	0.25588	0.0057216	0.016796	
beta. pop_ den	0.6959	0.38004	0.0084979	0.018124	
Vrho	9.9363	0.04228	0.0009454	0.006303	
Deviance	167.5602	20.00579	0.4473430	4.263603	

2. Quantiles for each variable:

	2.5%	25%	50%	75%	97.5%
beta.(Intercept)	-5.29025	-4.9219	-4.7412	-4.5516	-4.1893
beta.layer.1	1.95791	2.8337	3.4249	4.0775	5.8057
beta.layer.2	-4.54382	-3.0043	-2.3280	-1.8164	-1.1100

beta.layer -1.94214 -1.2997 -0.9614 -0.6568 -0.1935 beta.lc
-0.06632 0.2348 0.4243 0.6151 0.9767 beta.alt 0.04202
0.5225 0.7425 0.9846 1.3649 beta.lc_modi 0.75666 1.0526
1.2249 1.3973 1.7489 beta.pop_den 0.03395 0.4101 0.6681
0.9602 1.4821

Vrho	9.84410	9.9070	9.9430	9.9710	9.9968
Deviance	134.46400	153.4041	165.3311	179.1117	213.7345

Potential scale reduction factors:

Point est. Upper C.I.

beta.(Intercept) 1.16 1.56
beta.layer.1 1.15 1.52
beta.layer.2 1.10 1.37
beta.layer 1.24 1.78
beta.lc 1.31 1.98
beta.alt 1.01 1.04
beta.lc_modi 1.01 1.03
beta.pop_den 1.00 1.01
Vrho Deviance
1.01 1.01
1.27 1.95

Multivariate psrf 1.69

Coronaviridae- ZIB model
Inclusion of SARS CoV-2 origin

Iterations = 5001:9996 Thinning
interval = 5 Number of chains = 2
Sample size per chain = 1000

1. Empirical mean and standard deviation for each variable,
plus standard error of the mean:

Mean SD Naive SE Time-series SE
beta.(Intercept) -1.0375 0.08718 0.001949 0.003691
beta.layer.1 2.5893 0.38003 0.008498 0.058296
beta.layer.2 -1.7417 0.33506 0.007492 0.048560
beta.layer -0.6705 0.15486 0.003463 0.015303 0.3154
beta.lc 0.10546 0.002358 0.003524 0.9007 0.11034
beta.alt 0.002467 0.008849
beta.lc_modi
beta.pop_den 1.2216 0.10344 0.002313 0.003691
Deviance 0.8019 0.24268 0.005427 0.007636
1285.5064 4.31098 0.096397 0.279522

2. Quantiles for each variable:

2.5%25% 50% 75%
beta.(Intercept) -1.2049 -1.0953 -1.0379 -0.9791
beta.layer.1 1.8054 2.3529 2.5974 2.8567
beta.layer.2 -2.3346 -1.9802 -1.7525 -1.5269
beta.layer -0.9784 -0.7766 -0.6667 -0.5633
beta.lc beta.alt 0.1132 0.2431 0.3184 0.3861
beta.lc_modi -0.6775 0.8264 0.9014 0.9764
1.0218 1.1541 1.2215 1.2889
beta.pop_den 0.3647 0.6365 0.7873 0.9641
Deviance 1279.4800 1282.4205 1284.7211 1287.7234
97.5%beta.
(Intercept) -0.8671
beta.layer.1 3.2889
beta.layer.2 -1.0756
beta.layer -0.3737
beta.lc 0.5219
beta.alt 1.1200
beta.lc_modi
beta.pop_den 1.4294
Deviance 1.2899
1296.0396

Potential scale reduction factors:

Point est. Upper C.I.
beta.(Intercept) 1.01 1.02
beta.layer.1 1.08 1.19
beta.layer.2 1.07 1.16
beta.layer 1.04 1.11

beta.lc	1.00	1.02
beta.alt	1.01	1.05
beta.lc_modi	1.00	1.01
beta.pop_den	1.00	1.00
Deviance	1.02	1.06

Multivariate psrf

1.03

Coronaviridae- Binomial iCAR model
Inclusion of SARS CoV-2 origin

Iterations = 5001:9996 Thinning
interval = 5 Number of chains = 2
Sample size per chain = 1000

1. Empirical mean and standard deviation for each variable,
plus standard error of the mean:

Mean SD Naive SE Time-series SE
beta.(Intercept) 1.3221 0.5269 0.011782 0.083936
beta.layer.1 1.9397 0.5659 0.012655 0.094587
beta.layer.2 -0.9727 0.5200 0.011628 0.089489 -0.6421
beta.layer 0.2343 0.005239 0.027425 0.2138 0.1361
beta.lc 0.003044 0.006156 1.0704 0.1692 0.003782
beta.alt 0.012079
beta.lc_modi
beta.pop_den 1.3130 0.2429 0.005431 0.012950
10.2581 2.1242 0.047498 0.333385
gamma.(Intercept) 1.3278 0.2088 0.004668 0.015005
Deviance 1238.1997 4.4458 0.099410 0.267020

2. Quantiles for each variable:

2.5% 25% 50% 75%
beta.(Intercept) 0.40655 0.9159 1.2808 1.7086
beta.layer.1 1.00704 1.5414 1.8677 2.2640
beta.layer.2 -2.20851 -1.2713 -0.9190 -0.6073
beta.layer -1.12968 -0.7879 -0.6301 -0.4853
beta.lc -0.04953 0.1216 0.2082 0.3038
beta.alt 0.77241 0.9495 1.0595 1.1823
beta.lc_modi 0.90714 1.1365 1.2941 1.4625
beta.pop_den 6.63048 8.5868 10.2018 11.8183
gamma.(Intercept) 0.94351 1.1814 1.3156 1.4658
Deviance 1231.78006 1234.8944 1237.4213 1240.6048
97.5%
beta.(Intercept) 2.3746
beta.layer.1 3.2386
beta.layer.2 -0.0914

beta.layer - 0.2172
beta.lc 0.4888
beta.alt 1.4167
beta.lc_modi 1.8656
beta.pop_den 14.4870
gamma.(Intercept) 1.7759
Deviance 1248.8583

Potential scale reduction factors:

Point est. Upper C.I.
beta.(Intercept) 1.04 1.19

beta.layer.1	1.10	1.37
beta.layer.2	1.07	1.27
beta.layer	1.04	1.18
beta.lc	1.00	1.00
beta.alt	1.09	1.33
beta.lc_modi	1.00	1.01
beta.pop_den	1.04	1.18
gamma.(Intercept)	1.04	1.15
Deviance	1.01	1.03

Multivariate psrf

1.15

Coronaviridae- ZIB iCAR model
Inclusion of SARS CoV-2 origin

Iterations = 5001:9996 Thinning
interval = 5 Number of chains = 2
Sample size per chain = 1000

1. Empirical mean and standard deviation for each variable,
plus standard error of the mean:

Mean SD Naive SE Time-series SE
beta.(Intercept) -4.5484 0.27529 6.156e-03 0.019618
beta.layer.1 3.4225 0.87309 1.952e- 02 0.147034
beta.layer.2 -2.5347 0.77196 1.726e-02 0.129692
beta.layer - 0.9393 0.34825 7.787e-03 0.033309
beta.lc 0.3537 0.23934 5.352e-03 0.013159 0.7239
beta.alt 0.32173 7.194e-03 0.028046
beta.lc_modi 1.2559 0.22885 5.117e- 03 0.011594
beta.pop_den 0.5832 0.36399 8.139e- 03 0.018234
gamma.(Intercept) 796.8551 592.53081 1.325e+01 18.721051 Vrho
9.9046 0.04366 9.762e-04 0.006487 Deviance 171.2733 16.67269
3.728e-01 1.387722

2. Quantiles for each variable:

2.5% 25% 50% 75% 97.5%
beta.(Intercept) -5.05525 -4.7544 -4.5593 -4.3546 -4.0053 beta.layer.1
1.59953 2.8593 3.4294 3.9959 5.0928
beta.layer.2 -3.96861 -3.0312 -2.5386 -2.0806 -0.9069
beta.layer -1.63615 -1.1788 -0.9465 -0.7072 -0.2656
beta.lc -0.11484 0.1942 0.3636 0.5142 0.8201
beta.alt 0.11748 0.5173 0.7243 0.9253 1.4216
beta.lc_modi 0.81031 1.1000 1.2535 1.4090 1.7086
beta.pop_den -0.04103 0.3189 0.5609 0.8082 1.3255 gamma.(Intercept)
32.10191 337.8567 680.9352 1127.1751 2216.3527 Vrho 9.83245 9.8710
9.9007 9.9356 9.9891
Deviance 140.04840 159.1466 170.8898 182.8275 204.5376

Potential scale reduction factors:

	Point est.	Upper C.I.
beta.(Intercept)	1.02	1.09
beta.layer.1	1.05	1.08
beta.layer.2	1.15	1.53
beta.layer	1.02	1.03
beta.lc	1.09	1.36
beta.alt	1.06	1.24
beta.lc_modi	1.05	1.21
beta.pop_den	1.01	1.02
gamma.(Intercept)	1.00	1.00
Vrho	1.00	1.00

Deviance	1.03	1.07

Multivariate psrf

1.15

	Model	Deviance	Perc
1	NULL	1896.61687	0
2	env	571.05392	73
3	binomial	587.76539	72
4	binomial.icar	77.34814	100
5	ZIB.icar	74.03859	100

"masTSS"	"OA"	"prob"
1	1	0.44

Coronaviridae- ZIB iCAR model Random effects

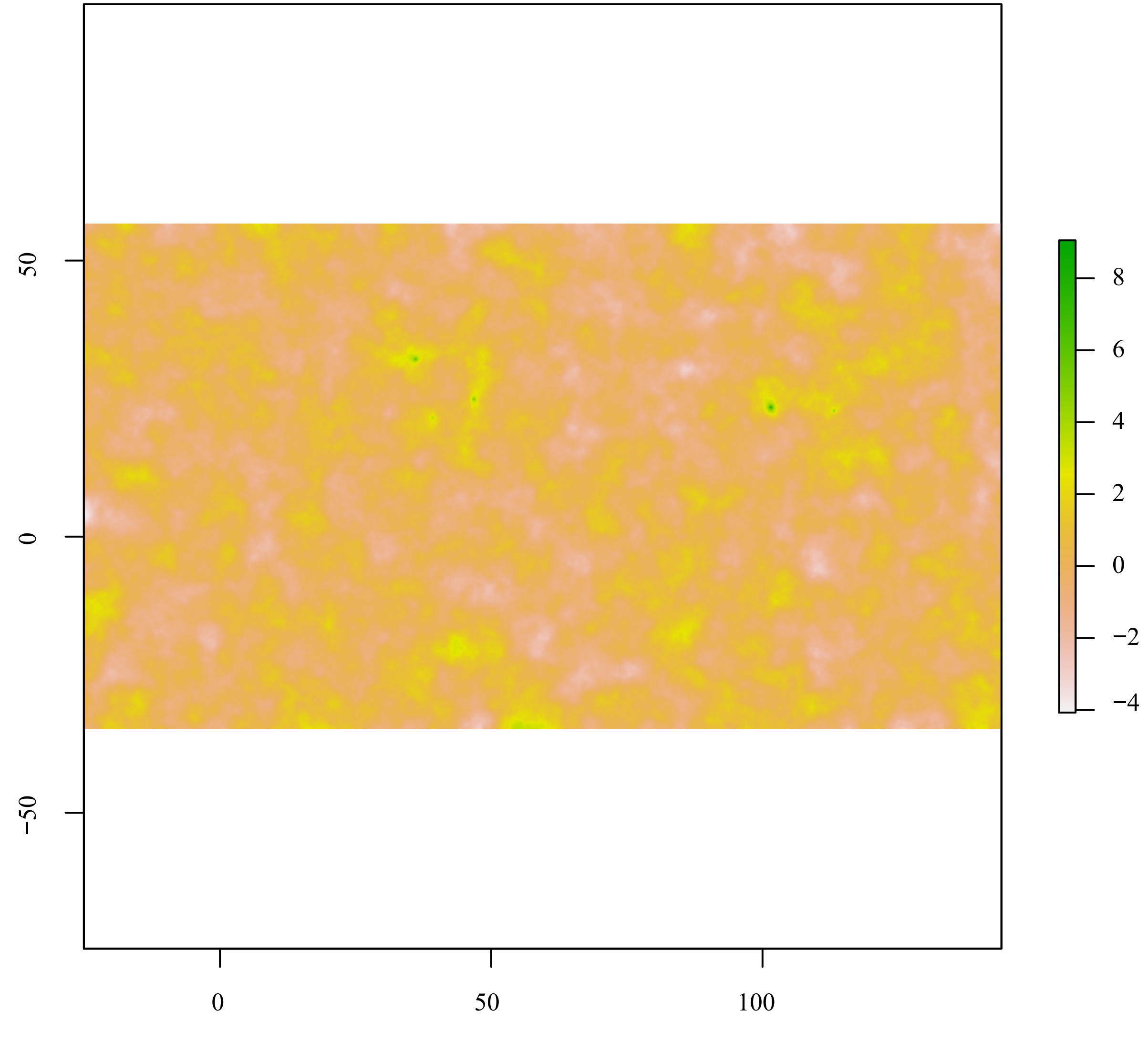

Coronaviridae hotspots with detection >TSS threshold
Inclusion of SARS CoV-2 origins

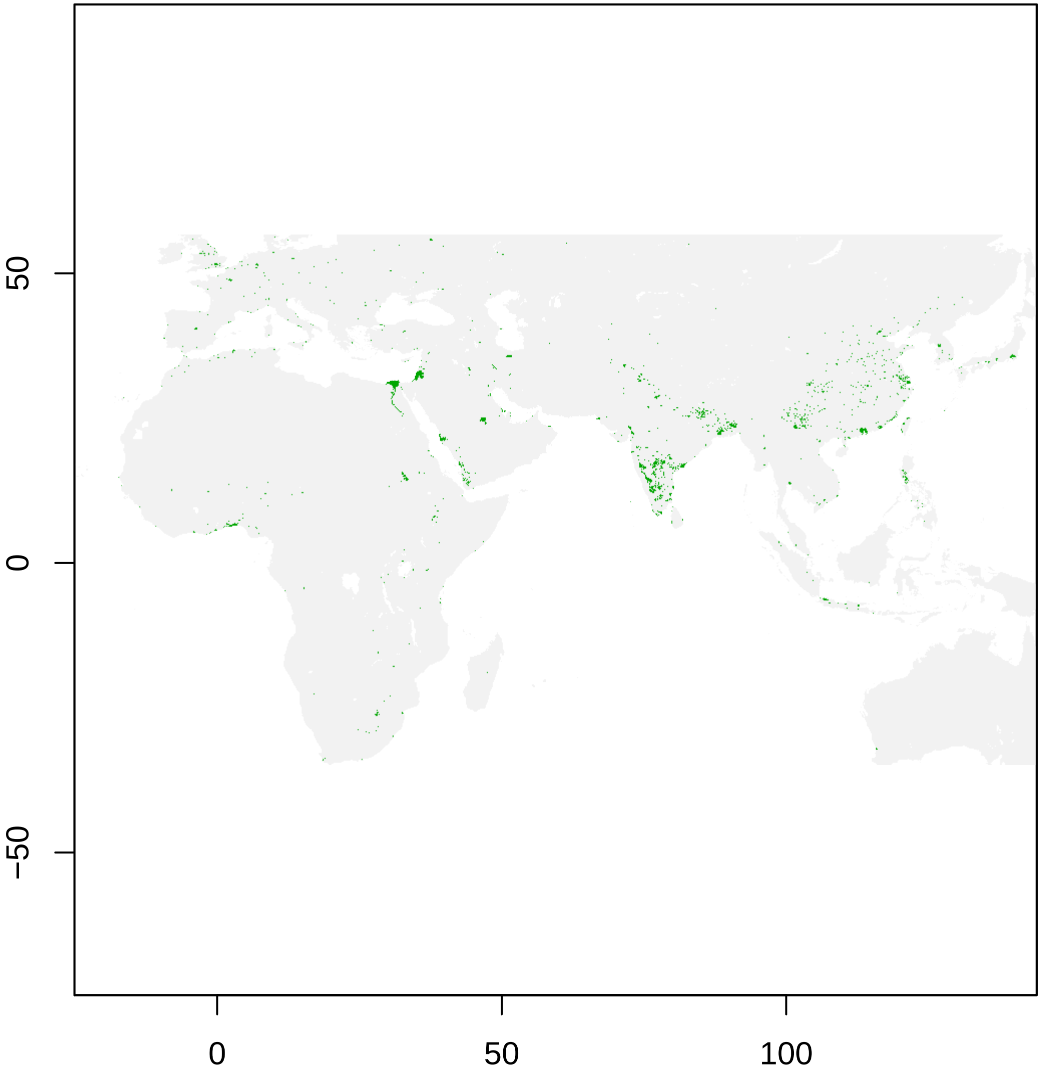

Supplement: Supplementary file 1 [file tropicalmed-07-00124-s001.zip › tropicalmed-1776466-supplementary.pdf]
